# Draft genome of a biparental beetle species, *Lethrus apterus*

**DOI:** 10.1186/s12864-021-07627-w

**Published:** 2021-04-26

**Authors:** Nikoletta A. Nagy, Rita Rácz, Oliver Rimington, Szilárd Póliska, Pablo Orozco-terWengel, Michael W. Bruford, Zoltán Barta

**Affiliations:** 1 MTA-DE Behavioural Ecology Research Group, Department of Evolutionary Zoology, University of Debrecen, Egyetem tér 1, Debrecen, H-4032 Hungary; 2 Department of Evolutionary Zoology and Human Biology, University of Debrecen, Debrecen, Hungary; 3 School of Biosciences, Cardiff University, Cardiff, UK; 4 Genomic Medicine and Bioinformatic Core Facility, Department of Biochemistry and Molecular Biology, Faculty of Medicine, University of Debrecen, Debrecen, Hungary

**Keywords:** Genome assembly, Parental behaviour, Coleoptera, Geotrupidae

## Abstract

**Background:**

The lack of an understanding about the genomic architecture underpinning parental behaviour in subsocial insects displaying simple parental behaviours prevents the development of a full understanding about the evolutionary origin of sociality. *Lethrus apterus* is one of the few insect species that has biparental care. Division of labour can be observed between parents during the reproductive period in order to provide food and protection for their offspring.

**Results:**

Here, we report the draft genome of *L. apterus*, the first genome in the family Geotrupidae. The final assembly consisted of 286.93 Mbp in 66,933 scaffolds. Completeness analysis found the assembly contained 93.5% of the Endopterygota core BUSCO gene set. Ab initio gene prediction resulted in 25,385 coding genes, whereas homology-based analyses predicted 22,551 protein coding genes. After merging, 20,734 were found during functional annotation. Compared to other publicly available beetle genomes, 23,528 genes among the predicted genes were assigned to orthogroups of which 1664 were in species-specific groups. Additionally, reproduction related genes were found among the predicted genes based on which a reduction in the number of odorant- and pheromone-binding proteins was detected.

**Conclusions:**

These genes can be used in further comparative and functional genomic researches which can advance our understanding of the genetic basis and hence the evolution of parental behaviour.

**Supplementary Information:**

The online version contains supplementary material available at 10.1186/s12864-021-07627-w.

## Background

Sociality among insects is highly diverse ranging from simple interactions to complex hierarchical societies [34]. However, the social behaviours and their genetical background have been investigated mainly in eusocial species with well-ordered colonies (e.g. [67, 81]). Therefore, the literature lacks studies on the molecular basis of social behaviour in simpler societies such as subsocial species [34]. Among these insects, diverse forms of parental care occur, including guarding for the eggs, food provision, protecting the freshly hatched offspring, and even biparental care with division of labour between the parents [43]. These behaviours appeared independently in 13 different insect orders, including 15 families of Coleoptera such as Scarabaeidae and Silphidae. Insect species with parental care are feasible subjects of sociogenomics aiming to understand the interaction between behaviour and genes regulating parental behaviour [63]. Therefore, knowing the mechanistic principles of parental care among subsocial insects could lead to a better understanding of social evolution [15]. Another pathway to inferring the origin of sociality is via comparative analysis of genomes of many organisms with analogous parental behaviours [13].

Beetles have evolved an extraordinary variety of life history strategies [50], including very diverse social and reproductive behaviours, from aggregation through creating nests to biparental care [9]. Despite of the fact that beetles represent the most diverse animal order, only a handful of coleopteran genomes have been published to date, including model species like the red flour beetle (*Tribolium castaneum*), a burying beetle, *Nicrophorus vespilloides* and important pests like the Colorado potato beetle (*Leptinotarsa decemlineata*) and the small hive beetle (*Aethina tumida*) [49]. However, of the sequenced beetle species, only *N. vespilloides* and *Onthophagus taurus* have parental care.

**Fig. 1:**
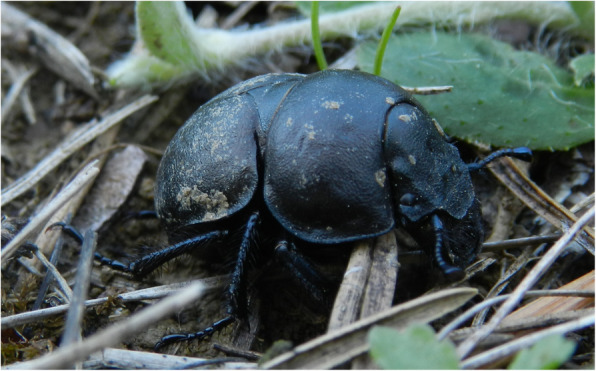
*Lethrus apterus* adult female (Susa, Hungary). Photo: Nikoletta A. Nagy

*Lethrus apterus* Laxmann 1770 (Coleoptera: Geotrupidae; Fig. 1) is one of the few insect species that has biparental care [12]. These beetles are only active during their breeding season, which lasts from early March to the beginning of June, and outside of this period the adults spend their time in a diapause in the soil [19]. At the beginning of their breeding season, adults choose mates with whom they excavate underground nests [64]. After this, a division of labour can be observed with females collecting leaves for each offspring in separate underground chambers while males guard the entrance of the nest from intruders, e.g. other *L. apterus* individuals or predators [65]. At the end of the reproductive period, adults dig themselves into the soil while the hatching larvae consume the stored leaves [33].

In this study, we report the draft de novo genome of *L. apterus* which is the first published genome in the family Geotrupidae. We performed functional annotation, and searched directly for potential parental behaviour regulator genes in the genome. Additionally, we investigated the single nucleotide polymorphism variants distribution based on samples from eight populations in Hungary.

## Results and discussion

### Assembly quality and completeness

**Fig. 2:**
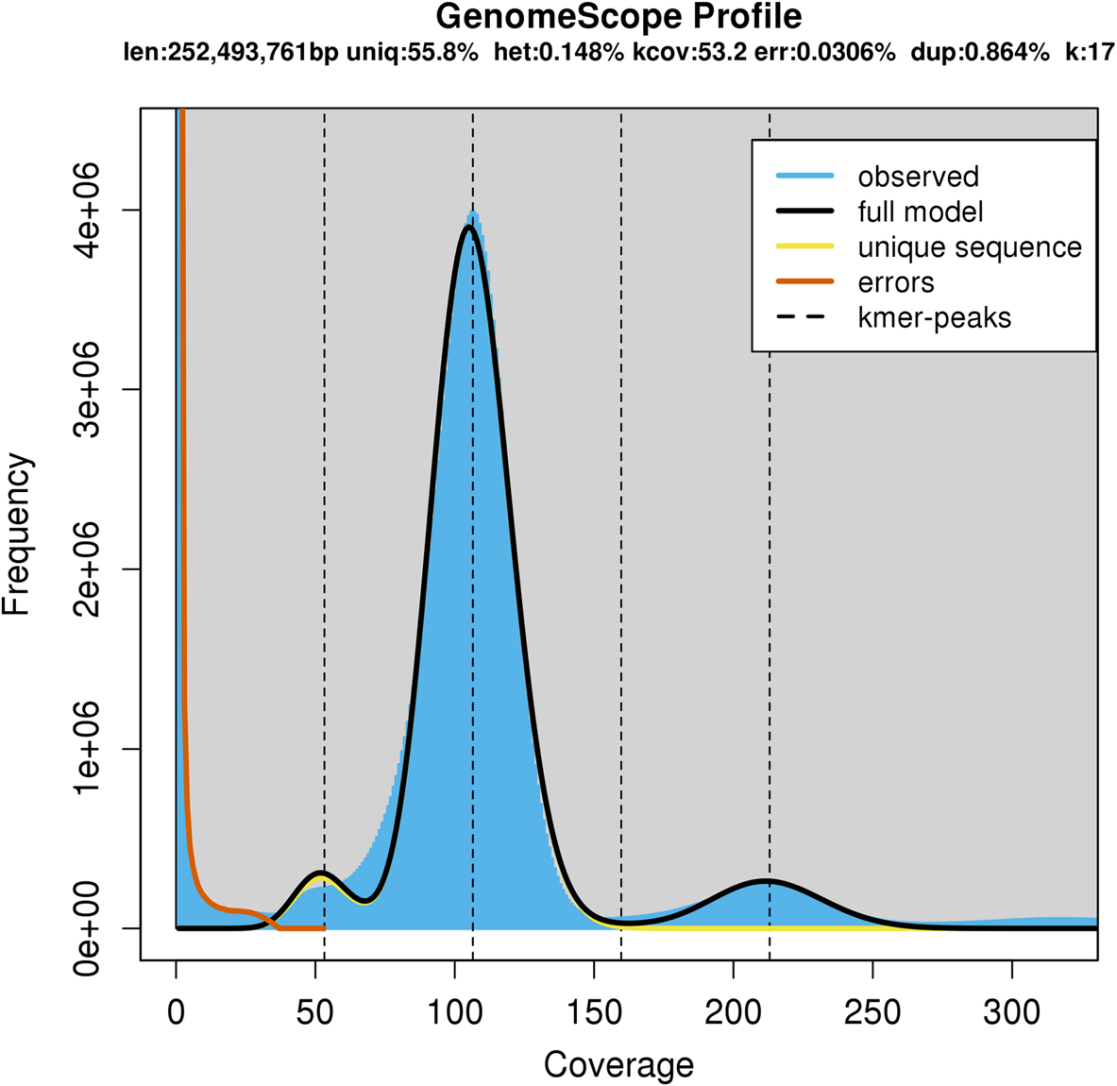
Genome and read characteristics produced by GenomeScope. Len: haploid genome length; uniq: overall length of unique (i.e. not repetitive) sequences; het: heterozygosity rate; kcov: mean k-mer coverage for heterozygous sequences; err: error rate of reads; dup: read duplication rate; k: k-mer length

The final assembly of *Lethrus apterus* comprised 66,933 scaffolds with an N50 value of 8902 bp (Table 1). The total length of the genome was estimated to be 286.93 Mbp, comparable with other beetle genomes published to date, and with the estimated size by GenomeScope (252.49 Mbp, Fig. 2). The GC content of the final assembly is 31.66%, similar to other beetle genomes. GenomeScope results showed a relatively low heterozygosity rate (0.148%) and low percentage of unique sequences (55.8%) which was probably caused due to the combined dataset (see Materials and methods) (Fig. 2). An additional peak was formed by the high frequency (0.864%) of duplicated k-mers which predicts high proportion of repeats in the genome [80]. High ratio of repeat regions together with short reads sequencing can lead to fragmented genome assemblies as repeats are often longer than the reads [54]. Therefore, low contiguity of our assembly, even after merging assemblies generated by diverse applications, is likely due to the high repeat sequence content to which the lack of a closely related reference genome contributed as well. Nevertheless, the *L. apterus* assembly has a high gene completeness, since 93.5% of BUSCOs from the Endopterygota database were detected (Table 2).

**Table 1:** Descriptive statistics of different assemblies produced during analyses

Statistics	MEGAHIT	MSG	SOAP	GAM	GAM_501
Number of scaffolds	146216	128507	207501	127406	66933
Longest scaffold (kbp)	99.34	115.32	125.01	114.98	114.98
Total length (Mbp)	306.62	307.02	230.73	307.78	286.93
N50 (bp)	7043	8046	5406	8140	8902
GC content (%)	31.81	31.71	38.62	31.63	31.66

MEGAHIT: assembly produced by MEGAHIT; MSG: assembly produced by the MEGAHIT-SSPACE-GapFiller pipeline; SOAP: assembly produced by SOAPdenovo2; GAM: assembly produced by merging assemblies MSG and SOAP; GAM_501: the GAM assembly with contaminant and short contigs removed (for details see Materials and Methods)

**Table 2:** Completeness of the different assemblies assessed by BUSCO

BUSCOs	MSG	(%)	SOAP	(%)	GAM	(%)	GAM_501	(%)	Predicted genes	(%)
Complete	1763	83.0	1906	89.8	1761	82.9	1985	93.5	1927	90.7
Single-copy	1749	82.3	1896	89.3	1746	82.2	1969	92.7	1128	53.1
Duplicated	14	0.7	10	0.5	15	0.7	16	0.8	799	37.6
Fragmented	110	5.2	131	6.2	112	5.3	91	4.3	92	4.3
Missing	251	11.8	87	4.0	251	11.8	48	2.2	105	5.0
Total	2124	100.0	2124	100.0	2124	100.0	2124	100.0	2124	100.0

Column headers are explained in legend of Table 1

**Fig. 3:**
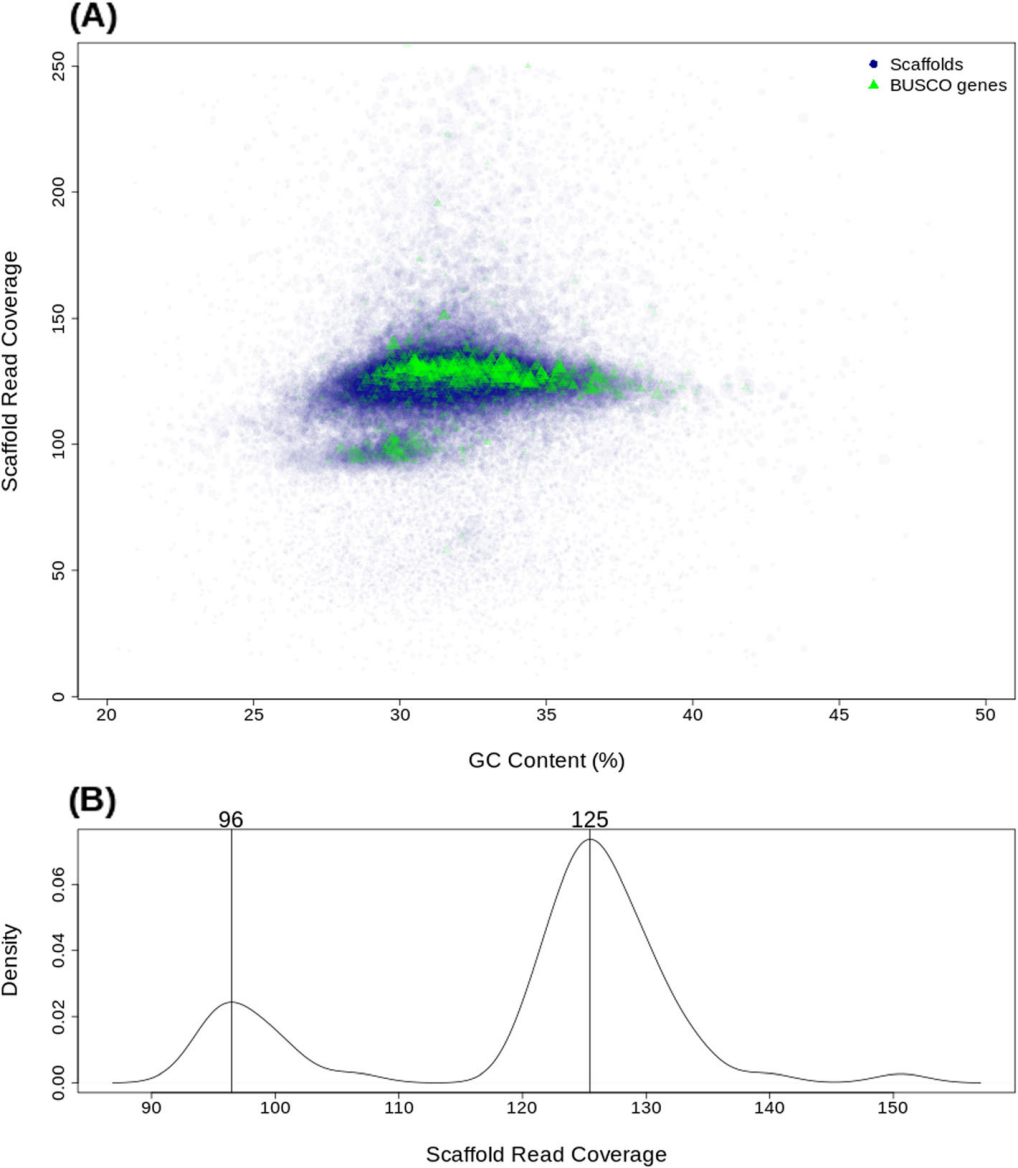
Read coverage distribution. **a** The distribution of scaffolds in the GC content – read coverage state space. Blue symbols mark scaffolds, size of the symbol is proportional to the length of the scaffold. Green symbols show BUSCO genes, size of the symbol is proportional to the length of the scaffold containing the gene. **b** Density plot of read coverage of scaffolds of size longer than 5 kbp. The numbers above the peaks show the corresponding coverage values

**Fig. 4:**
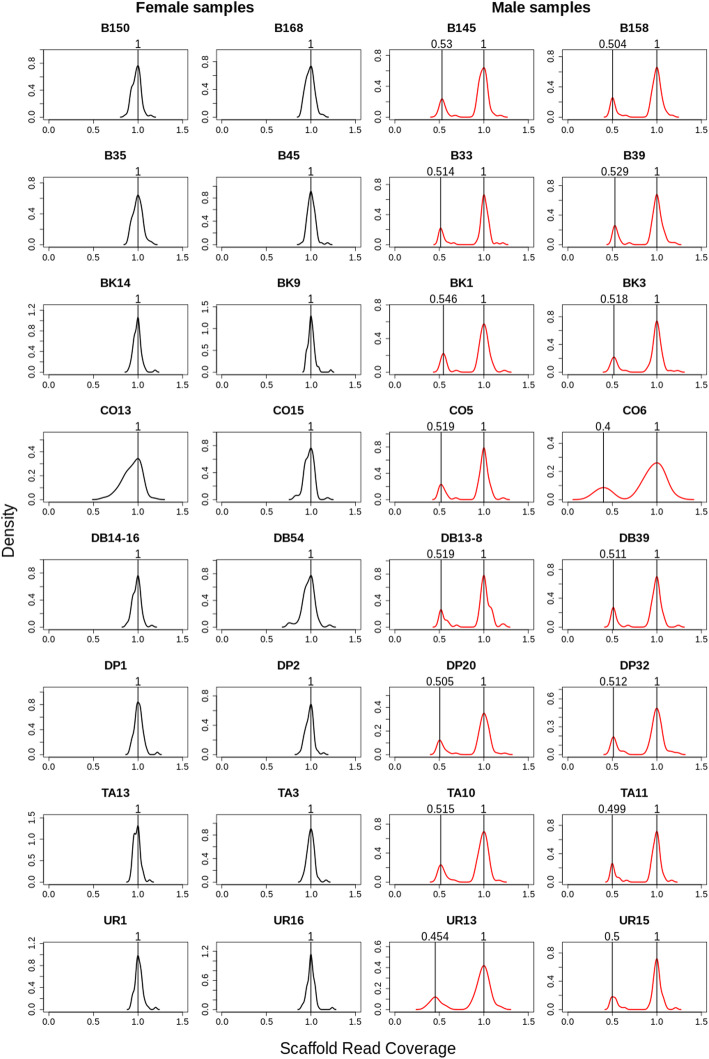
Density plots of read coverage of scaffolds of size longer than 5 kbp. Female samples are shown with black whereas males are shown with red lines. The coverage values are rescaled so that the coverage value with maximum density is one. Numbers at the top of the plot show the coverage values of the peak densities. The relative coverage was 0.504 ± 0.03 in the lower coverage group of males

The distribution of contigs analysed for their coverage and GC content state space resulted in scaffolds separated into two groups according to their read coverage (Fig. 3). Both groups contained genes identified by the BUSCO analysis. The quotient of the coverage of the two groups was 3/4 and the proportion of males and females in the combined sample (see Materials and methods) was 1:1, suggesting that the group of scaffolds with lower coverage could reflect sequences from the chromosomes. This is further supported by Fig. 4 showing that the lower coverage group of scaffolds are only present in males. This suggests an XY or X0 sex determination system in *Lethrus apterus*.

### Genome annotation

RepeatMasker was used to identify repetitive elements in the assembly of *Lethrus apterus*. Results showed that a high proportion (36.44% of bases) of the genome contains interspersed repeats, most of which (71.46%) could not be classified as known repeats (Table 3). The most abundant repeats were A-rich sequences with low complexity. Only three of the next 10 repeat classes showed significant matches with the NCBI nt database. One matched with an inverted repeat in the pannier region of the harlequin ladybeetle (*Harmonia axyridis*), the other two had hits with uncharacterised genome regions of the mountain pine beetle (*Dendroctonus ponderosae*) and the ringlet butterfly (*Aphantopus hyperantus*).

**Table 3:** Repetitive elements found by RepeatMasker

Element type	Num	LO (Mbp)	PS
Total interspersed repeats	421620	104.58	36.44%
SINEs	0	0	0.00%
LINEs	42401	11.19	3.90%
LTR elements	2279	1.00	0.35%
DNA elements	60124	17.66	6.15%
Unclassified	316816	74.73	26.04%
Small RNA	0	0	0.00%
Satellites	0	0	0.00%
Simple repeats	77082	3.23	1.12%
Low complexity	17892	0.88	0.31%

Num: number of elements; LO: length occupied in mega base-pairs; PS: percentage of element type with regard to the assembled genome sequence

Ab initio gene prediction resulted in 25,385 sequences whereas homology based prediction found 22,551. After merging and filtering the two gene sets, 34,392 remained from which 20,734 were functionally annotated by InterProScan or Diamond using different databases. The annotated gene set had a 1425 bp mean CDS length, 475 amino acids mean protein length and contained 5.10 exons and 4.10 introns per gene on average.

Based on the functional annotation, the potential sex chromosome related genes coded mostly proteins necessary for cell maintenance, including housekeeping genes and mitochondrial proteins, however, some transposons and retrotransposons were found. In addition, proteins involved in the innate immune responses, circadian rhythm and memory were also identified.

### Annotation of reproductive behaviour related genes

Based on a literature search, 23 candidate genes were found in 21 research articles (Table 4). Of these, 19 genes were found in coleopteran species and were stored in NCBI. All 19 candidate genes had significant hits with the predicted genes of *Lethrus apterus*, sequences of the hits can be found as Additional file 1. Compared to the other examined beetle species, *L. apterus* had lower number of hits of odorant-binding and pheromone-binding proteins. These molecules play a significant role in recognition of the signals of the environment, such as food resources or recognition of conspecifics [21]. The loss of these genes may be a great starting point of a research on the evolution of olfactory perception among dung beetles, however, we should note that the low number of hits could be caused by the fragmented genome hence further investigation would require the involvement of other data, such as RNA sequencing. In addition, two of the 19 genes, namely troponin C, and octopamine receptor were found among genes located on potentially sex chromosomes and thus may serve as targets for future research on gene regulation of reproductive behaviour.

**Table 4:** Candidate genes involved in reproductive behaviour among coleopterans

Gene name (reference)	Hits in Tc	Hits in Nv	Hits in Ot	Hits in Av	Hits in La
*Fruitless* [87]	33	28	34	26	28
*Sex peptide receptor* [25, 87]	29	10	14	10	7
*Apolipophorin-III* [5, 59]	1	2	8	1	3
*Octopamine receptor* [14]	64	57	61	49	64
*Insulin receptor substrate* [84]	12	2	5	0	3
*Krüppel homolog* [84]	3	4	2	1	2
*Target of rapamycin* [84]	1	1	1	1	1
*Odorant binding protein* [32, 62, 85]	55	66	54	40	20
*Glucose oxidase* [62]	–	–	–	–	–
*Alpha-glucosidase precursor* [62]	–	–	–	–	–
*Troponin C* [62]	33	37	35	19	29
*Vitellogenin* [66]	5	7	8	4	6
*Vitellogenin receptor* [66]	11	12	17	11	13
*Juvenile hormone acid o-methyltransferase* [83]	4	8	30	1	13
*Malvolio* [51]	4	5	5	2	5
*Neuropeptide F* [78]	1	2	3	1	2
*Methyl geranate* [78]	–	–	–	–	–
*Odorant receptor* [85, 90]	266	51	83	259	63
*Pheromone-binding protein* [68]	36	52	40	33	13
*Cryptochrome* [86]	2	2	1	1	1
*Sex peptide* [2]	–	–	–	–	–
*Accessory gland protein* [16, 60]	3	6	1	2	1
*Insulin-like peptide* [82]	7	4	13	5	4

Column 2 includes the number of genes found on NCBI Protein database. Columns 2–6 represent the number of Diamond hits in the coleopteran proteomes. *Tc* Tribolium castaneum. *Nv* Nicrophorus vespilloides, *Ot* Onthjophagus taurus, *Av* Asbolus verrucosus, *La* Lethrus apterus

Fragmented genome assembly can lead to over-prediction of paralogous genes, especially in case of gene families with high number of similar members [44]. The results of the candidate gene search, however, showed that the number of hits in genes of *Lethrus apterus* and the related species were similar, suggesting that the low contiguity of the assembly did not influence the gene prediction.

### Comparison with other coleopteran species

**Fig. 5:**
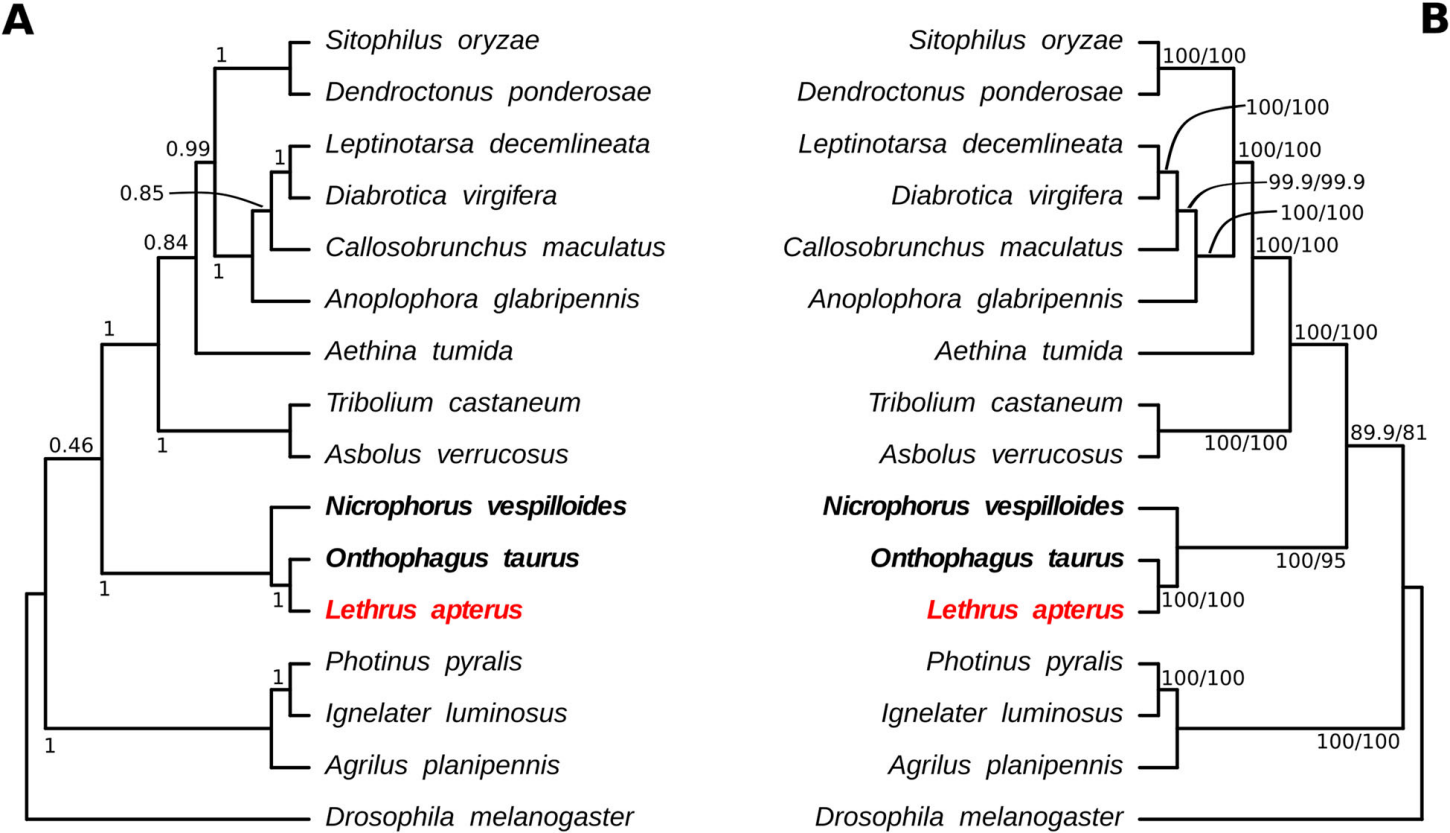
Phylogenetic relationships of 14 coleopteran species rooted with *Drosophila melanogaster* as outgroup. Tree was constructed based on 37 common single-copy protein sequences. Species with parental care are highlighted in bold and *Lethrus apterus* is additionally highlighted in red. **a** Phylogram generated with coalescent based estimation, support values are local posterior probabilities. **b** Concatenation-based phylogenetic tree, support values are ultrafast bootstrap/aLRT

Fourteen coleopteran proteomes available on NCBI and predicted *Lethrus apterus* genes were used to perform comparison of orthologous genes by Orthofinder. Based on the results, 357,992 genes (95.9% of the total number of genes) were assigned to 23,528 orthogroups. All species were present in 4754 orthogroups of which 44 included only single-copy genes. 9618 orthogroups were species-specific from which 607 (consisting of 1664 genes) were specific to *L. apterus*. Of the predicted genes, 436 were not assigned to any orthogroups. Finally, 42.1% of the orthogroups contained *L. apterus* genes. Our phylogenetic results are in line with those relationships described in [88, 89]. Based on the species trees reconstructed with two independent methods, *Nicrophorus vespilloides* appeared to be the sister taxa of *Lethrus apterus + Onthophagus taurus.* Monophyly of these groups received a high statistical support in all of our analyses (Fig. 5). This branching not only marks the divergence of Staphylinoidea and Scarabeoidea, but also separates those only three species in our dataset that have biparental care. Further studies are now needed to more precisely decipher the origin of biparental care among beetles.

### Variant calling

The three approaches used (see Materials and methods) produced a large number of variant loci; samtools: 2768768 (9.65 SNPs/kbp), GATK: 2804771 (9.77 SNPs/kbp), and freebayes: 3384895 (11.79 SNPs/kbp), respectively. After the filtering steps, only 237,835 SNP loci identified by all three methods remained. From these loci 12.65% were missing, however, different samples had different portion of missing variants, some had as low as 1%, while other had as high as 31%. The average number of missing variants per locus was 4.0.

From the 237,835 variants, 22,593 (9.5% of the total) were found in exonic regions, 24,231 (9.9%) in intronic regions and 191,797 (80.6%) in intergenic regions. Seven hundred eighty-six variants were reported in both exonic and intronic regions which suggests e.g. nested or reverse coding genes. The mean variant density was 0.83 SNPs/kbp varying between coding (0.62 SNPs/kbp), intron (0.71 SNPs/kbp) and intergenic regions (0.85 SNPs/kbp).

Based on the principal component analysis, the samples forming our Susa dataset were grouping together which supports our theory that there should be low variation between these populations (Figure S1). One interesting finding was that one population, namely the Debrecen population was separated from all of the other populations. This clustering can serve as great basis of future studies thusthese variant loci can serve as a resource for future population genomics analysis in *Lethrus apterus*.

## Conclusion

We have reported the genome of *Lethrus apterus*, the first genome in the family Geotrupidae. Although the assembly is highly fragmented, probably due to the repetitive nature of the genome, it has a high level of gene completeness. This beetle species is a good model for division of labour during parental care. All genes related to reproductive and parental behaviours published so far were located in the final genome assembly, therefore, its use for future investigation for the genetic architecture of parental care among insects should be pursued. Further, potential sex chromosome related genes were identified which can be useful for uncovering the genetic basis of the division of labour between the parents.

## Materials and methods

### Sampling and DNA extraction

In the spring of 2016, 32 individuals were collected from eight locations (2 males and 2 females from each location) across Hungary. Samples were taken from thorax muscles from each individual. Each tissue sample was ground in a clean mortar using pestle and liquid nitrogen and when grinding was finished 430 μl of extraction buffer was added. According to Gilbert et al. [23], the buffer contained 3 mM CaCl2, 2% sodium dodecyl sulphate (SDS), 40 mM dithiotreitol (DTT), 250 μg/ml proteinase K, 100 mM Tris buffer pH 8 and 100 mM NaCl (final concentrations). The solution was suspended into a clean 1.5 ml microcentrifuge tube and 50 μl RNase was added. Suspensions were vortexed and centrifuged for 30 s at 400 G. Samples were incubated overnight at 56 °C until no tissue clumps were visible. After the mixtures were cooled down at room temperature, 0.5 volume of 7.5 M ammonium-acetate was added to each sample and vortexed, and then put in a − 20 °C freezer for 10–15 min. After this, 1 volume of chloroform:isoamylalcohol 24:1 was added to each sample and the tubes were vortexed for 2 s. Samples were centrifuged for 5 min at 18000 G and the supernatant was carefully transferred to a clean tube. This step was repeated once more. Two volumes of ice cold 96% ethanol were added and samples were mixed by inverting, then incubated on − 20 °C overnight. Tubes were centrifuged at 18000 G for 10 min and the liquid phase was carefully removed from the pellets, then 500 μl 70% ethanol was added. After inverting, samples were centrifuged at 5000 G for 3 min, then the pellets were dried at room temperature. Finally, 50–100 μl of 10 mM Tris-EDTA (pH = 9.0) buffer was added to dissolve the pellets.

### DNA library preparation and sequencing

The sequencing library was prepared using 300 ng of DNA fragmented with a Bioruptor sonicator (Diagenode) with high mode 10 cycles setting (30 s on/30 s off). For the library preparation the TruSeq® Nano DNA LT Sample Preparation Kit (Illumina) was used following the manufacturer’s protocol. The individually barcoded libraries were diluted to 10 nM and two pools of 16 samples each were prepared for sequencing. Paired-end sequencing of 125 bp reads was performed on a HiSeq2500 machine at the EMBL GeneCore Facility (Heidelberg).

Individual sequence coverage was on average low (~10x), therefore, the reads of 12 individuals collected in neighbouring regions (Susa, Uraj and Ózd) were combined to achieve a higher coverage that would facilitate genome reconstruction. The base of the sample choice was their close spatial proximity, hence less variation was expected between them. Hereafter, we refer to this dataset of combined samples as the Susa dataset.

### Quality control and filtering

The read quality of the Susa dataset was checked with FastQC (v0.11.5, [3]). The proportion of bases with a base quality of 30 or higher was above 90% in all samples. Trimmomatic (v0.36, [8]) was used to eliminate any adapter sequences from the reads, with at least 97% of the reads passing the trimming process. Next, the Susa reads were combined into two FASTQ files, one for the forward and one for the reverse sequence, and because these reads derived from different individuals, Musket (v1.1, [41]) was used to reduce the variation introduced by combining samples. After this correction, the quality of the dataset was checked with SGA-PreQC (v0.10.15, [72]). Results showed 20% were PCR duplicates, high mean quality score (>Phred 35), low sequencing error rate (< 5 × 10^− 5^), a peak of 51-mer distribution at count 70, and also predicted high repeat branch frequency similar to the human and oyster genomes. Additionally, GenomeScope (v1.0 [80];), which works very well on short reads, was run on the 17-mer count histogram of the Susa dataset after Musket produced using Jellyfish (v2.2.6 [47];) to check heterozygosity and repeat region contant.

### Genome assembly

MEGAHIT (v1.1.1, [38]) was used (minimum k-mer size 51, maximum k-mer size 111, k-mer step size 20) to generate the initial assembly of the Susa reads and contig length was increased through running three cycles of SSPACE (v3.0, [6, 7]) alternating with GapFiller (v1.10, [7, 54]). Another assembly of the Susa reads was run using SOAPdenovo2 (v2.04, [46]) with the default k-mer size. These two assemblies were then merged with gam-nsg (v1.1b, [79]) to improve contig sizes. The final assembly was obtained by removing contaminant and duplicated contigs as reported by GenBank Contamination Screen and the potentially poor quality contigs shorter than 501 bp as short contigs can bias downstream analyses, e.g. mislead the gene prediction methods and the variant calling.

To assess the completeness of our assemblies, BUSCO (v4.1.2, [71]) was run to identify the proportion of the Endopterygota gene set that was found in the *Lethrus* genome. For this, the *Tribolium castaneum* gene set (tribolium2012) was used for algorithm training. The distribution of coverage and GC along the contigs was also analysed in R statistical environment (v3.5.2 [61];).

### Genome annotation

The distribution of repetitive sequences was analysed with RepeatModeler (version open-1.0.8, [73]) and RepeatMasker (version open-4.0.7, [74]). First, a de novo repeat library was built using RepeatModeler. RepeatMasker was used for repeat analysis based on RepeatModeler output and Repbase (version 20170127).

For gene prediction in the soft masked genome assembly, ab initio and homology-based prediction methods were combined with the BRAKER pipeline (v2.1.5 [26, 27];). Ab initio prediction was carried out using Augustus (v3.3.3 [75, 76];). For homology-based prediction, hints for GeneMark-EP (GeneMark-ES Suite v4.57_lic [45];) were generated with ProtHint (v2.4.0 [10];) based on the endopterygota_db10. Augustus was then trained with these sequence hints with default settings and used for prediction. This was followed with another round of training and prediction based on the first iteration results and GeneMark hints. Predicted coding sequences containing premature stop codons and shorter than 50 amino acids were filtered out.

Before annotation, the ab initio and homology-based predicted genes were merged using CD-HIT (v4.7 [42];) to cluster genes with the exact same amino acid sequences with the parameters ‘-c 1 -G 0 -aL 1.0 -aS 1.0’. The merged gene dataset was functionally annotated on the public server at usegalaxy.eu [1] using InterProScan (v5.36–75.0 [29];) with Pfam [18], PANTHER [52], ProDom [69], PROSITE [70], SMART [37], SUPERFAMILY [57], TIGRFAM [24] and PRINTS [4] databases. Additionally, Diamond (v2.0.6.144 [11];) against Uniprot (downloaded on 6th May 2020; UniProt Consortium 2019 [77]) database with an e-value of 10^− 5^ was used to further annotate the predicted genes.

### Analysis of reproductive behaviour related genes

To identify genes potentially involved in parental behaviour, a literature search was carried out on 12th May 2020 on Web of Science using the following “Topic” keywords: (insect* AND (“reproductive behavior” OR “reproductive behaviour” OR “parental care”) AND (gene OR genes)). This resulted in 96 articles from which a list of candidate genes was extracted on the basis of the genes being identified as playing a role in insect reproductive behaviour. NCBI was searched and filtered for complete and reliable protein sequences (not partial, predicted, putative, hypothetical, unknown or uncharacterised) of coleopteran species. These sequences were used as query for searching homologous proteins among the predicted genes of *Lethrus apterus* using Diamond (in sensitive mode, with e-value set to 10^− 5^ and query coverage to 40%). Besides, the same search was run against the proteomes of the following species: *Tribolium castaneum* which has a well annotated genome; *Nicrophorus vespilloides* that is a model species for parental care among insects; *Onthophagus taurus*, the most closely related species with an available genome; *Asbolus verrucosus* which has a highly fragmented genome, therefore can be a scale for the accuracy of our result from the assembly of *L. apterus*.

### Orthology and phylogenetic analysis

Orthofinder (v2.3.12 [20];) was used to find orthologs and species-specific genes and also create phylogenetic tree to reveal the phylogenetic relationship of *Lethrus apterus* to the other beetle species. For this purpose, proteomes of all available coleopteran species (*Aethina tumida*, *Agrilus planipennis*, *Anoplophora glabripennis*, *Asbolus verrucosus*, *Callosobruchus maculatus*, *Dendroctonus ponderosae*, *Diabrotica virgifera*, *Ignelater luminosus*, *Leptinotarsa decemlineata*, *Nicrophorus vespilloides*, *Onthophagus taurus*, *Photinus piralis*, *Sitophilus oryzae* and *Tribolium castaneum*) and *Drosophila melanogaster* as an outgroup were downloaded from NCBI. For inferring the species tree, orthogroups containing all species with only single-copy genes were used. Amino acid sequences by orthogroups were first aligned with MUSCLE (v3.8.1551 [17];) using default parameters. A concatenation-based phylogenetic tree was estimated using IQ-TREE (v1.6.9 [55];) by specifying the gene-based partitioning scheme (i.e. allowing a different substitution model for each gene). Branch support was tested using 1000 ultrafast bootstrap replications (−bb 1000) and by a likelihood-ratio test (−alrt 1000). To further strengthen our results a coalescent based estimation of the species phylogeny was also applied as implemented in ASTRAL-III (v5.7.3 [88, 89];). Maximum likelihood gene trees were inferred individually for each alignment also by using IQ-TREE (v1.6.9 [55];). Branch support values were assessed by 1000 ultrafast bootstrap replications (−bb 1000). Branches with a lower bootstrap support than 70 were collapsed into polytomies using nw_ed from Newick Utilities (v1.6 [30];). Using these 37 individual gene trees as input, coalescence-based species tree was estimated with ASTRAL-III (v5.7.3 [88, 89];). In all runs of IQ-TREE the best scoring substitution matrix was simultaneously estimated by ModelFinder Plus (−MFP [31];) and the best fitting substitution model was used to reconstruct the resulting phylogenetic tree. The cophyloplot function of the R package “ape” (v5.4 [58];) was used for visualisation.

### Variant identification

Cleaned reads for each individual sample were aligned to the final assembled genome using BWA mem (v0.7.15-r1140, [39]). The resulting sam files were converted to sorted and indexed bam files with samtools (v1.2, [40]). Since base correction might cause bias in the number of variants, three different methods were used for variant calling which was followed by rigorous filtering steps. The first set of variants were called with the samtools mpileup – bcftools call pipeline (bcftools v1.3, [40]). Variants were also identified with GATK (v3.6.0.g89b7209, [48]) and freebayes (v1.1.0–3-h961e5f3, [22]). To run GATK, picard was used to mark duplicates, create a sequence directory of the reference genome, add to read group information and build a bam index. Then GATK Haplotypecaller was run for each sample separately and the results were combined with GenotypeGVCFs. For freebayes, all samples were called simultaneously.

Freebayes produced the highest number of polymorphisms (see Results), therefore, we filtered them to remove loci which had at least one variant with a missing genotype, a minimum alternate count of less than two, a minimum coverage of less than four or a maximum coverage higher than 25. After this filtering, loci which had variants with Phred based genotype likelihood less than 20 were marked as missing, i.e. they were removed from the data of the given sample. From these loci only those which were also called by GATK and samtools were retained. From the filtered loci found by all three methods, those positioned in low complexity (identified using dustmasker v1.0.0, [53]) or repetitive regions were removed. From the remaining variants, only SNPs were retained.

Variants were located in the structurally annotated genome using the R packages “GenomicFeatures” (v1.34.8 [36];), “rtracklayer” (v1.42.2 [35];) and “VariantAnnotation” (v1.28.13 [56];). After this, multiallelic SNPs were filtered out and the genetic variation between populations were investigated with principal component analysis using R package “adegenet” (v2.1.3 [28];).

## Supplementary Information

**Additional file 1. **Amino acid sequences of genes that are potentially involved in the regulation of reproductive behaviours in *Lethrus apterus*.

**Additional file 2: Figure S1.** Principal component analysis plot of samples from eight populations of *Lethrus apterus*. The first two components (PCs) are plotted and the sample names are included. Eigenvalues are shown in the bottom right corner. The red ellipse includes the samples forming the Susa dataset.

**Additional file 3: Table S1.** Information on the NCBI accession numbers of the raw reads for each sample.

## Data Availability

Raw read sequences were submitted to NCBI SRA database. A summary table can be found in Table S1. The final assembly of the draft genome has been deposited at GenBank under the accession JAFFZR000000000. The version described in this paper is version JAFFZR010000000. Annotation files (GFF3, amino acid and nucleotide sequences) were uploaded to Zenodo (https://zenodo.org/record/4540796). Protein sequences of the 14 beetle species (*Aethina tumida*, *Agrilus planipennis*, *Anoplophora glabripennis*, *Asbolus verrucosus*, *Callosobruchus maculatus*, *Dendroctonus ponderosae*, *Diabrotica virgifera*, *Ignelater luminosus*, *Leptinotarsa decemlineata*, *Nicrophorus vespilloides*, *Onthophagus taurus*, *Photinus piralis*, *Sitophilus oryzae* and *Tribolium castaneum*) and *Drosophila melanogaster* were downloaded from NCBI GenBank with the following accession numbers: GCF_001937115.1, GCF_000699045.2, GCF_000390285.2, GCA_004193795.1, GCA_900659725.1, GCF_000355655.1, GCF_003013835.1, GCA_011009095.1, GCF_000500325.1, GCF_001412225.1, GCF_000648695.1, GCF_008802855.1, GCF_002938485.1, GCF_000002335.3, GCF_000001215.4.

## References

[1] Afgan E, Baker D, Batut B, Van Den Beek M, Bouvier D, Čech M, et al. The Galaxy platform for accessible, reproducible and collaborative biomedical analyses: 2018 update. Nucleic Acids Res. 2018;46(W1):W537-W544. https://doi.org/10.1093/nar/gky379. 10.1093/nar/gky379 PMC6030816 29790989

[2] Aigaki T, Fleischmann I, Chen PS, Kubli E (1991). Ectopic expression of sex peptide alters reproductive behavior of female *D. melanogaster*. Neuron..

[3] Andrews S. FastQC: A quality control tool for high throughput sequence data. https://www.bioinformatics.babraham.ac.uk/projects/fastqc/. Accessed 19 Dec 2020.

[4] Attwood TK, Coletta A, Muirhead G, Pavlopoulou A, Philippou PB, Popov I, Romá-Mateo C, Theodosiou A, Mitchell AL. The PRINTS database: a fine-grained protein sequence annotation and analysis resource—its status in 2012. Database. 2012;2012:bas019. 10.1093/database/bas019 PMC3326521 22508994

[5] Benowitz KM, McKinney EC, Roy-Zokan EM, Cunningham CB, Moore AJ (2017). The role of lipid metabolism during parental care in two species of burying beetle (*Nicrophorus spp.*). Anim Behav.

[6] Boetzer M, Henkel CV, Jansen HJ, Butler D, Pirovano W (2011). Scaffolding pre-assembled contigs using SSPACE. Bioinformatics..

[7] Boetzer M, Pirovano W (2012). Toward almost closed genomes with GapFiller. Genome Biol.

[8] Bolger AM, Lohse M, Usadel B (2014). Trimmomatic: a flexible trimmer for Illumina sequence data. Bioinformatics..

[9] Brandmayr P (1992). Short review of the presocial evolution in Coleoptera. Ethol Ecol Evol.

[10] Bruna T, Lomsadze A, Borodovsky M (2020). GeneMark-EP and-EP+: automatic eukaryotic gene prediction supported by spliced aligned proteins. NAR Genom Bioinform.

[11] Buchfink B, Xie C, Huson DH (2015). Fast and sensitive protein alignment using DIAMOND. Nat Methods.

[12] Clutton-Brock TH (1991). Forms of parental care. The evolution of parental care.

[13] Cunningham CB (2020). Functional genomics of parental care of insects. Horm Behav.

[14] Cunningham CB, Douthit MK, Moore AJ (2014). Octopaminergic gene expression and flexible social behaviour in the subsocial burying beetle *Nicrophorus vespilloides*. Insect Mol Biol.

[15] Cunningham CB, Ji L, Wiberg RAW, Shelton J, McKinney EC, Parker DJ, Meagher RB, Benowitz KM, Roy-Zokan EM, Ritchie MG, Brown SJ (2015). The genome and methylome of a beetle with complex social behavior, *Nicrophorus vespilloides* (Coleoptera: Silphidae). Genome Biol Evol.

[16] Dottorini T, Nicolaides L, Ranson H, Rogers DW, Crisanti A, Catteruccia F (2007). A genome-wide analysis in Anopheles gambiae mosquitoes reveals 46 male accessory gland genes, possible modulators of female behavior. PNAS..

[17] Edgar RC (2004). MUSCLE: multiple sequence alignment with high accuracy and high throughput. Nucleic Acids Res.

[18] El-Gebali S, Mistry J, Bateman A, Eddy SR, Luciani A, Potter SC, Queshi M, Richardson LJ, Salazar GA, Smart A, Sonnhammer ELL, Hirsh L, Paladin L, Piovesan D, Tosatto SCE, Finn RD (2019). The Pfam protein families database in 2019. Nucleic Acids Res.

[19] Emich G (1884). Die metamorphose des *Lethrus apterus*. Matematische und Naturwissenschaftliche Berichte aus Ungarn.

[20] Emms DM, Kelly S (2019). OrthoFinder: phylogenetic orthology inference for comparative genomics. Genome Biol.

[21] Fan J, Francis F, Liu Y, Chen JL, Cheng DF (2011). An overview of odorant-binding protein functions in insect peripheral olfactory reception. Genet Mol Res.

[22] Garrison E, Marth G. Haplotype-based variant detection from short-read sequencing. ArXiv. 2012;1207:3907.

[23] Gilbert MTP, Moore W, Melchior L, Worobey M (2007). DNA extraction from dry museum beetles without conferring external morphological damage. PLoS One.

[24] Haft DH, Selengut JD, Richter RA, Harkins D, Basu MK, Beck E (2012). TIGRFAMs and genome properties in 2013. Nucleic Acids Res.

[25] Hanin O, Azrielli A, Zakin V, Applebaum S, Rafaeli A (2011). Identification and differential expression of a sex-peptide receptor in *Helicoverpa armigera*. Insect Biochem Mol Biol.

[26] Hoff KJ, Lange S, Lomsadze A, Borodovsky M, Stanke M (2016). BRAKER1: unsupervised RNA-Seq-based genome annotation with GeneMark-ET and AUGUSTUS. Bioinformatics..

[27] Hoff KJ, Lomsadze A, Borodovsky M, Stanke M, Kollmar M (2019). Whole-genome annotation with BRAKER. Gene prediction.

[28] Jombart T (2008). Adegenet: a R package for the multivariate analysis of genetic markers. Bioinformatics..

[29] Jones P, Binns D, Chang HY, Fraser M, Li W, McAnulla C, McWilliam H, Maslen J, Mitchell A, Nuka G, Pesseat S, Quinn AF, Sangrador-Vegas A, Scheremetjew M, Yong S, Lopez R, Hunter S (2014). InterProScan 5: genome-scale protein function classification. Bioinformatics..

[30] Junier T, Zdobnov EM (2010). The Newick utilities: high-throughput phylogenetic tree processing in the UNIX shell. Bioinformatics..

[31] Kalyaanamoorthy S, Minh BQ, Wong TK, von Haeseler A, Jermiin LS (2017). ModelFinder: fast model selection for accurate phylogenetic estimates. Nat Methods.

[32] Kim IH, Pham V, Jablonka W, Goodman WG, Ribeiro JM, Andersen JF (2017). A mosquito hemolymph odorant-binding protein family member specifically binds juvenile hormone. J Biol Chem.

[33] Kosztolányi A, Nagy N, Kovács T, Barta Z (2015). Predominant female care in the beetle *Lethrus apterus* with supposedly biparental care. Entomol Sci.

[34] Kronauer DJ, Libbrecht R (2018). Back to the roots: the importance of using simple insect societies to understand the molecular basis of complex social life. Curr Opin Insect Sci.

[35] Lawrence M, Gentleman R, Carey V (2009). Rtracklayer: an R package for interfacing with genome browsers. Bioinformatics..

[36] Lawrence M, Huber W, Pages H, Aboyoun P, Carlson M, Gentleman R, Morgan MT, Carey VJ (2013). Software for computing and annotating genomic ranges. PLoS Comput Biol.

[37] Letunic I, Bork P (2018). 20 years of the SMART protein domain annotation resource. Nucleic Acids Res.

[38] Li D, Liu CM, Luo R, Sadakane K, Lam TW (2015). MEGAHIT: an ultra-fast single-node solution for large and complex metagenomics assembly via succinct de Bruijn graph. Bioinformatics..

[39] Li H. Aligning sequence reads, clone sequences and assembly contigs with BWA-MEM. ArXiv. 2013;1303:3997.

[40] Li H, Handsaker B, Wysoker A, Fennell T, Ruan J, Homer N, Marth G, Abecasis G, Durbin R (2009). 1000 genome project data processing subgroup. The sequence alignment/map format and SAMtools. Bioinformatics..

[41] Liu Y, Schröder J, Schmidt B (2013). Musket: a multistage k-mer spectrum-based error corrector for Illumina sequence data. Bioinformatics..

[42] Fu L, Niu B, Zhu Z, Wu S, Li W. CD-HIT: accelerated for clustering the next-generation sequencing data. Bioinformatics. 2012;28(23):3150–2. 10.1093/bioinformatics/bts565. 10.1093/bioinformatics/bts565 PMC3516142 23060610

[43] Gilbert JD, Manica A (2015). The evolution of parental care in insects: a test of current hypotheses. Evolution..

[44] Indrischek H, Wieseke N, Stadler PF, Prohaska SJ (2016). The paralog-to-contig assignment problem: high quality gene models from fragmented assemblies. Algorithms Mol Biol.

[45] Lomsadze A, Ter-Hovhannisyan V, Chernoff YO, Borodovsky M (2005). (2005). Gene identification in novel eukaryotic genomes by self-training algorithm. Nucleic Acids Res.

[46] Luo R, Liu B, Xie Y, Li Z, Huang W, Yuan J, He G, Chen Y, Pan Q, Liu Y, Tang J, Wu G, Zhang H, Shi Y, Liu Y, Yu C, Wang B, Lu Y, Han C, Cheung DW, Yiu S, Peng S, Xiaoqian Z, Liu G, Liao X, Li Y, Yang H, Wang J, Lam T, Wang J (2012). SOAPdenovo2: an empirically improved memory-efficient short-read de novo assembler. Gigascience..

[47] Marçais G, Kingsford C (2011). A fast, lock-free approach for efficient parallel counting of occurrences of k-mers. Bioinformatics..

[48] McKenna A, Hanna M, Banks E, Sivachenko A, Cibulskis K, Kernytsky A, Garimella K, Altshuler D, Gabriel S, Daly M, DePristo MA (2010). The genome analysis toolkit: a MapReduce framework for analyzing next-generation DNA sequencing data. Genome Res.

[49] McKenna DD (2018). Beetle genomes in the 21st century: prospects, progress and priorities. Curr Opin Insect Sci.

[50] McKenna DD, Farrell BD, Hedges SB, Kumar S (2009). Beetles (Coleoptera). The Timetree of life.

[51] Mehlferber EC, Benowitz KM, Roy-Zokan EM, McKinney EC, Cunningham CB, Moore AJ (2017). Duplication and Sub/Neofunctionalization of Malvolio, an Insect Homolog of Nramp, in the subsocial beetle *Nicrophorus vespilloides*. G3.

[52] Mi H, Muruganujan A, Ebert D, Huang X, Thomas PD (2019). PANTHER version 14: more genomes, a new PANTHER GO-slim and improvements in enrichment analysis tools. Nucleic Acids Res.

[53] Morgulis A, Gertz EM, Schäffer AA, Agarwala R (2006). A fast and symmetric DUST implementation to mask low-complexity DNA sequences. J Comput Biol.

[54] Nadalin F, Vezzi F, Policriti A (2012). GapFiller: a de novo assembly approach to fill the gap within paired reads. BMC Bioinformatics.

[55] Nguyen LT, Schmidt HA, Von Haeseler A, Minh BQ (2015). IQ-TREE: a fast and effective stochastic algorithm for estimating maximum-likelihood phylogenies. Mol Biol Evol.

[56] Obenchain V, Lawrence M, Carey V, Gogarten S, Shannon P, Morgan M (2014). VariantAnnotation: a bioconductor package for exploration and annotation of genetic variants. Bioinformatics..

[57] Pandurangan AP, Stahlhacke J, Oates ME, Smithers B, Gough J (2019). The SUPERFAMILY 2.0 database: a significant proteome update and a new webserver. Nucleic Acids Res.

[58] Paradis E, Claude J, Strimmer K (2004). APE: analyses of phylogenetics and evolution in R language. Bioinformatics..

[59] Park YC, Yoo JS, Jun SS, Kim TH, Kim JK, Choe JC, Kim HB (2012). Identification of differentially expressed genes from adult cockroach females exhibiting maternal care behavior. J Asia Pac Entomol.

[60] Ram KR, Wolfner MF (2007). Sustained post-mating response in *Drosophila melanogaster* requires multiple seminal fluid proteins. PLoS Genet.

[61] R Core Team (2018). R: A language and environment for statistical computing.

[62] Rehan SM, Berens AJ, Toth AL (2014). At the brink of eusociality: transcriptomic correlates of worker behaviour in a small carpenter bee. BMC Evol Biol.

[63] Robinson GE, Grozinger CM, Whitfield CW (2005). Sociogenomics: social life in molecular terms. Nat Rev Genet.

[64] Rosa ME, Barta Z, Fülöp A, Székely T, Kosztolányi A (2017). The effects of adult sex ratio and density on parental care in *Lethrus apterus* (Coleoptera, Geotrupidae). Anim Behav.

[65] Rosa ME, Barta Z, Kosztolányi A (2018). Willingness to initiate a fight but not contest behaviour depends on intruder size in *Lethrus apterus* (Geotrupidae). Behav Process.

[66] Roy-Zokan EM, Cunningham CB, Hebb LE, McKinney EC, Moore AJ (2015). Vitellogenin and vitellogenin receptor gene expression is associated with male and female parenting in a subsocial insect. Proc R Soc B Biol Sci.

[67] Rubin BE, Jones BM, Hunt BG, Kocher SD (2019). Rate variation in the evolution of non-coding DNA associated with social evolution in bees. Philos Trans R Soc B.

[68] Senthilkumar R, Srinivasan R (2019). Sex-specific spatial and temporal gene expressions of pheromone biosynthesis activating neuropeptide (PBAN) and binding proteins (PBP/OBP) in *Spoladea recurvalis*. Sci Rep.

[69] Servant F, Bru C, Carrere S, Courcelle E, Gouzy J, Peyruc D, Kahn D (2002). ProDom: automated clustering of homologous domains. Brief Bioinform.

[70] Sigrist CJ, de Castro E, Cerutti L, Cuche BA, Hulo N, Bridge A, Bougueleret L, Xenarios I (2012). New and continuing developments at PROSITE. Nucleic Acids Res.

[71] Simão FA, Waterhouse RM, Ioannidis P, Kriventseva EV, Zdobnov EM (2015). BUSCO: assessing genome assembly and annotation completeness with single-copy orthologs. Bioinformatics..

[72] Simpson JT (2014). Exploring genome characteristics and sequence quality without a reference. Bioinformatics..

[73] Smit AFA, Hubley R (2008). RepeatModeler Open-1.0.

[74] Smit AFA, Hubley R, Green P (2013). RepeatMasker Open-4.0.

[75] Stanke M, Diekhans M, Baertsch R, Haussler D (2008). Using native and syntenically mapped cDNA alignments to improve de novo gene finding. Bioinformatics..

[76] Stanke M, Schöffmann O, Morgenstern B, Waack S (2006). Gene prediction in eukaryotes with a generalized hidden Markov model that uses hints from external sources. BMC Bioinformatics.

[77] The UniProt Consortium. UniProt: a worldwide hub of protein knowledge. Nucleic Acids Res. 2019;47(D1):D506-D515. https://doi.org/10.1093/nar/gky1049. 10.1093/nar/gky1049 PMC6323992 30395287

[78] Trumbo ST (2018). Juvenile hormone and parental care in subsocial insects: implications for the role of juvenile hormone in the evolution of sociality. Curr Opin Insect Sci.

[79] Vicedomini R, Vezzi F, Scalabrin S, Arvestad L, Policriti A (2013). GAM-NGS: genomic assemblies merger for next generation sequencing. BMC Bioinformatics.

[80] Vurture GW, Sedlazeck FJ, Nattestad M, Underwood CJ, Fang H, Gurtowski J, Schatz MC (2017). GenomeScope: fast reference-free genome profiling from short reads. Bioinformatics..

[81] Warner MR, Mikheyev AS, Linksvayer TA (2017). Genomic signature of kin selection in an ant with obligately sterile workers. Mol Biol Evol.

[82] Wigby S, Slack C, Grönke S, Martinez P, Calboli FC, Chapman T, Partridge L (2011). Insulin signalling regulates remating in female *Drosophila*. Proc R Soc B Biol Sci.

[83] Wijesekera TP, Saurabh S, Dauwalder B (2016). Juvenile hormone is required in adult males for *Drosophila* courtship. PLoS One.

[84] Woodard SH, Bloch GM, Band MR, Robinson GE (2014). Molecular heterochrony and the evolution of sociality in bumblebees (*Bombus terrestris*). Proc R Soc B Biol Sci.

[85] Wu ZZ, Qu MQ, Pu XH, Cui Y, Xiao WY, Zhao HX, Bin SY, Lin JT (2017). Transcriptome sequencing of *Tessaratoma papillosa* antennae to identify and analyze expression patterns of putative olfaction genes. Sci Rep.

[86] Xu J, Gao B, Shi MR, Yu H, Huang LY, Chen P, Li YH (2019). Copulation exerts significant effects on mRNA expression of Cryptochrome genes in a moth. J Insect Sci.

[87] Yapici N, Kim YJ, Ribeiro C, Dickson BJ (2008). A receptor that mediates the post-mating switch in *Drosophila* reproductive behaviour. Nature..

[88] Zhang C, Rabiee M, Sayyari E, Mirarab S (2018). ASTRAL-III: polynomial time species tree reconstruction from partially resolved gene trees. BMC Bioinformatics.

[89] Zhang SQ, Che LH, Li Y, Liang D, Pang H, Ślipiński A, Zhang P (2018). Evolutionary history of Coleoptera revealed by extensive sampling of genes and species. Nat Commun.

[90] Zhao HX, Xiao WY, Ji CH, Ren Q, Xia XS, Zhang XF, Huang WZ (2019). Candidate chemosensory genes identified from the greater wax moth, *Galleria mellonella*, through a transcriptomic analysis. Sci Rep.

