# Supplementary material for: Draft genome of a biparental beetle species, Lethrus apterus
Source: BMC Genomics. 2021 Apr 26;22:301. doi: 10.1186/s12864-021-07627-w (PMC8074431; doi:10.1186/s12864-021-07627-w)
Supplement: Supplementary file 1 — Additional file 1. Amino acid sequences of genes that are potentially involved in the regulation of reproductive behaviours in Lethrus apterus. [file 12864_2021_7627_MOESM1_ESM.pdf]

Fruitless

>abi333.t1_1
MGSEHYCLRWNNHQSNLLGVFSQLLRDESLVDVTLACSEGHSIRAHKVVLSACSSYFQTLFVDHPS
RHPIVILKDVRFSELRTLIEFMYKGEVNVEYCQLSALLKTAESLKVKGLAEMTNLGPSRDYKQEAE
PEQTEPTELVSKRQEDREHVPTPPKLCRSPERPSSVEPARSESPTEECKPSDMSMHNNGNNVSSCS
ILGTSAACNRLSSPINNEPLPGPSNMPPVQQVPLRLKYIIYSCDLKDVVTTLGVFNHSFD
>abi573.t1_1
MESAQQQQFCLKWNSFGSNLATSLSNLFKCESLADVTLFCDGVTFKAHKLILAACSKHLADLFETA
PLHQNLLVILDGTSAANMAALLEFMYKGEVHVSQDALSSFLKAAECLQVKGLSIEHEKLAVAHSSG
RVEKHPPLSEEAERPSPYSPMSPYMHPPHYRPYDQKPLDRRPLRSPNEMLQESVRASVLRDGSKAG
HPPSPGPMSLAYRPSSSSSCSLPTQQEADAPPEHRFDTEPAATALICPEPSSKQHCDPGCPEDLRM
KMEPIVQDEPPSSTASGNGISSSPSNGPIRHISGYENDRDKTDLIPNTLWNSVTNKLSHKSSSVST
PDGKKLKCPFCERLYGYETNLRAHIRQRHQGIRVPCPFCSRTFTRNNTVRRHIAREHKTELSLKAF
QQSQQVHNHNPVACMCLWRVRAGTVRLPHQVALVIECKQPSYQWYDSTQEFITQ
>abi1431.t1_1
MSVVDDQQFCLRWNNFQANITSQFETLRDDEDFTDVTIACDGQRLQAHKVVLSACNFYINNKSDAF
QSNPCPHPIIFMRDVEARHVVALMEFMYAGEVNVAQAHLSAFLRTAESLKIRGLTDTSPSEPFKGR
EDDTVFLNPQPSSSKHYLSPSFEKHISNKSQISSTSTVTSSQDISYSSDKPSTPPAKRQCKSERSE
SELSRLRQLSALAEDSITVNPFALQPDIRNSTLEISKSPIHPKLEYLSDDDRDESANFYPSPDATE
LAGGMEIIPQISNIMLKENSIETGTGTDSKKLHSLDPRPCEECGRVYSNLSNLRQHMKLIHYPTYV
QCTICQKDFKTDLYLRRHMLSSHEQSKVGLNEKYVNRPQNRGPIRLRRRRNSDNWLQFDLPISDKS
ICPQSDFSSRQRDGREGQLINRLVKLGEGIEIYEDQLRSVKWSDYRKLTRGLATILFSPAELATCS
VTGQRWSRAGSGERPVKPALDRAKVQAIISYVATRFPMVEISRIKQVLAYKCKENSTALKMKAVRY
YGDQPRDLDKN
>abi3941.t1_1
MDQQFCLRWNNHPTNLTDVLLSLLRRQALCDVTLACDGEMLRAHQTILSACSPYFEMLLIKNAHPH
PIIYLKDVNYVEMKALLDFMYKGEVNVSQNLLPMFLKTAEALQIRGLTDNNTLNSKSENAMETARR
ERPAEQESPPHEKRKRKSSSNCDTPSSNQDRSNDSQALSSYKATILRKLNPVVSVDKESNAEDMMR
VSSPLVKQELPHDENPHIDSYHCMSEALQT
>abi4795.t1_1
MNSLNYRYNTHPAQFQPGIASPAPANMSSQRFCLRWNNHQSNLLSVFDQLLHDESFVDVTLAVEGK
LLKAHKMVLSACSPYFQTLFVNHPDKHPIVILKDVPYNDMKSLLDFMYRGEVSVDQDRLTAFLRVA
ESLRIKGLTEVNEEKCDSITSSLTQQNQHLNSNLSQLQRLQPPTKRFNSLNMLGNALLQPKRKRGR
PRKLSGSSNGTPGADDFDRTDNLVQGSPEMLEVKMGMDGFTGNASDTSSGKNDRDNEMEETSVKEE
TEPIAGTSKEHNFNSSIAKSNENEFPPSDDKEKSPNVRCIDFNSSLHEEYNLPYQELLTSDTEVEE
NDNDLQEIDANYETLDIKVDAKTFIENMMDNNSNSNTPKKRKNSSCYDFEEYGNYEIILDDSSFDF
RPTKYEKSNRKISDPDKSARDFCVREKDNLYRCTVCDRVYTHISNFCRHYMTSHRLDVKMFSCPVC
MKDFTRKDNMLAHLKIIHKQQS
>abi4912.t1_1
MTSFRPVSSLTFAFGRYVNIIVLMSSWCFQGTQMTAAKMTMQQYCLKWNNHQPNFISVFSSLLNSE
SLVDVTLSAEGRHLQAHKLVLSACSSYFQTLFTINPCQHPIVILKDVKFTDLKVMVDFMYYGEVNV
SQEQLPHILKTAEMLKIKGLAEVPVESSISRHNSSLERTELVTPGESNWVSEPGQRQSMTPSPCPL
SPGSRRKRLRKSSTGSGSGSTERTSEEQVGNEIMLTSPSGTLATVIKPDPGGYVTDTGTTHRIKQH
SSDSDVHRSSSHDSVDSEATHQITLQIPQQQDINISHNVESSQTMEMPGPSSSTLQVAGFQWGIVD
HHTYPSHYVSCQTNVMHNDMQYVTANNQNPSPTNQPDVKPALNHITNSPPQIMQKRKRSLNPQADE
NFVRALEAVRFGGIGFCKAARMYGVNNRTLWLEYKKRGYPISRLSIKNRKPENTQQQQIQQPPSPP
NQQPQENEVVVSTVNNPIAMISSAFLEGRPVEIAPVLQRSRFFDNGIMNGQPMNLQGLNFEPM
>abi12596.t1_1
MVVDDQYSLNWGNHTHHIKKSFDSLWRDNDFVDVTLCCQGKKLKAHRMILSACSSYFHDVLRDNPC
AHPIVILRDVEYETMAGILQFMYKGEVQVPTENFSSFLKTAELLEVSGLVTPDSCSGPLNDETKDV
LKNVPKDKGKDVKKELIKDTEPNEEKLLASDDELERSKPAPASKKLERKKLLEAKKRQSESSIEEK
KKPEPKKRHSDSDDSQQKKKKPEEEITPEDNGPDEEDDQEMIDIMDPVNVQKLSNYQALLSDASYL
TKEANPECECPVCAQKFASIYYLKQHIPFHTGETKCQICGNVFSRKDLLRRHQASVHKTIVKSSTT
K
>abi12921.t1_1
MENEENLAGQDSTQHYNLKWTNYVNNILLVFAENLNNELLTDVTLTCEGKFIKAHKILLSACSSYF
HQLFQVYLVPNPLIILNGVRYNDLRQIIQYIYQGEIKVTEHDIDSILALAENLQIKGLCNIKFRNK
IEENLRKSVAKVSYHDKNENVSDDTMRNKRKRTMTPILSESKENEDSASGNSSEMPCNDRGHHNLP
LSVPHPDFHTVCILLLKYSTPLTKEMDNPKFGKERS

>abi13126.t1_1
MQHATGSPQQFCLRWNNYQSNLTNVFDQLLQSESFVDVTLACDGHSVKAHKMVLSACSPYFQSLFF
ENPCQHPIVIMRDIKWPELKAAVEFMYKGEINVSQEQIGPLLKVAESLKIRGLADVNGEQDVIGPP
GELTSTKTTAKLSSTPEWDNRQDGDNALRAKKRRRPSGERSSLSSPAESASTEVPEPPPSVEMSPA
TSATPLPPPGSQEPLPLTLPLQQPIPSHTSDDMEIKPGIAEMIREEERSTEYKNESALKISVPYLS
VKNTRKLDERETLRRVRSLRFSPPHRNCQMSSTRSLLVFIYLGHVNR
>abi15265.t1_1
MAAPPQQFCVRWNSYQSNLQNAFPKLLTSEHFVDVTLACENEMLKCHKVVLSACSTYFEKLLLDNP
CQHPIIFMKDMRFQEMQSLVDFMYKGEVNVTQDDLPSLLKSAEALQIRGLCGSDQLLNHQNFTNLR
TPSMVGTPVDKMQSSTPTQKPLEQNLKPIQKKEEKTDLPATESCLNPSSSECDSNDVPLKDEPESI
KEEIEEDSYYEGESELMDSFLDDEVPLNSIEGDDKTPDFAIANVSCQYDGSPDLNMRIGIHSGSVL
CGVLGLRKWQFDIWSYDVQLANHMESGGIPGRVHISESTLLCLNGAYEVEPGTGQERDCYLRDQDV
KTFLIKQVEPMRTRRRLASRPSIFSNKLWPEDDVISAASSPQSPATPASPPAPPSRERSDSGIQHS
TVDEENTTDWTPEIPFENLHHTLSGDLEEDIDSLNFSLNKQKYEIFHERKKPASFPLTTTEQVDEM
MDHSIEIESNKRMRNANVNPWTLRFKDQQMEHQVHI
>abi17081.t1_1
MLPQQYCLRWRYHHSNLQTMFSQLLEREAFCDVVLACEGRTIKAHKIVLSACSTYFDTILSQYEEK
DPILIMKDVKYVDIKCLVEFMYKGEINVDHCHLATLLKTAEELRIKGLAEVSWRDEECNNEGNTNG
IQSAALPQVSTVMESPKSGGESGNKRKRGRPPIDDYEQSPHQQSPHQQVFTPKIVSVTGNADETYS
NDAMSSSDHDLSIWEEETLGNDMEEANESEEPPVKVKTEMTNDDDIIMEDAVDPISTNEVKTEAVV
KILNSTHTKTNSTTTNTNSFNNSSSLTAALEKEWPDVIKMNDYLNTGRRQQFWEEPFTKRVMDAIK
TKNLEMKIAAELLGVSYGTLYGRYRDAYGCLKHPYRVRDFWTEQGPTDVLLKLKRKEITLFRAAEQ
LNVTPQTLSNYLISMSVLDNEANISGHSNPNETFDDGDSEEDTDSILPDVPSNSGLFKSAMTSNNS
TAATSSNSVLANCPDITIIKKEKLESATKVNNNSDHSDRQAGNSDQKGKLATDVLLKLKRKQITFD
GAVDILNVTPKSLSMYLEAISKMDDGKNISTDDMDDDVLQARAQMEQSVLVRCPDLTIVKTRRLEE
KSM
>abi17866.t1_1
MEGEQFSLCWNNFHNNLSSGFHTLLQDEDLVDVTLAAEGKFLKAHKTVLSVCSPYFKELFRMNPCK
HPIVILQDVSYSALHNLLQFMYQGEVSVSQEEIPTFMRVAETLRVKGLTDNSSNPELNGLDNSPMN
SPQTYGSLPDSKQFVKRRPIKKVLKPIQHLKPIESPRSMKSPPIARSSPYVPVKQPRLEEPAIPTP
EYGHSVVNPKQEPIDPNDDEFKDQSYSEHMDMSSMLDTTLGESSDSKSHLMSPSHKVPTDTSSPX
>abi18284.t1_1
MGGDTSPEQQYALRWNDFHSSILSSFRHLRDEEDFVDVTLACDGCSFTAHKVVLSACSPYFRRLLK
ANPCQHPIVILRDVQQKDMENLLRFMYNGEVHIGQEHLADFLKTAQMLQVRGLADVPAGSAGQKLT
VPEQKVSPIKSKNIEINLQNSSSLPWATDRSDALRDSGLSPPPLKRSRSTENSDSYIPDRVRSASR
GNIDMQESLLGQALEGGPTIHTNAKGNSDSPPRAQSTGDDSSSDTQMSDHERDPITPKTEPTDYPM
MDDHPFSTNGGLIDQSRTPSFPGALINLPGLPGLIPGPSSLHGGADSFEERHQMRMNRGNLKIESL
ASEIKL
>abi19029.t1_1
MGSTAQQYCLRWNNHRSNLLTVFDELLQNEAFTDVTLVCDGGGPIKCHRMVLAACSPYFQNLFTDL
PCKHPVVVLKDVKYCEIKAILEYMYRGEVNVAQDQLAALLKVAEALKVKGLVEENRTNEQNNMTRR
DDEPMEHSPSGITTSTTNTHSAVHNSSSNTSPPHSTNGVLYSKNPYNLYGKAPGIERNSRMNIPMW
AVPGIPLPHHPSPVPPQNHPHTAATAAAVMLSSCYEAASSDMSPLRRKKLSSLLMNRDTPILRTVL
GQGQADSSQPVSLVCHPDNHHENVNTNAPDERVIRHMKNEPSEDAQSPYTDLSMMEDEEKSKMGIP
SSSPQSYSGEMRSISSGIATYVPNQKPEWKRYKQYTRNDIMSAIEAVRNGMSALQAARKYGVPSRT
LYDKVKKLGITTSRPFKRGSNGSTACFPYGLSGTSSPYENEEHGMNNNSALLEASSFLQHALDGRG
GDEREALAAMAAAAAAHAAATGHSTSPPNHGMARSPSPSPSFIKYMRQSSLTPSPAPCSEHLHSET
NGSSERDRDDDDDQVEDLSIGRKQESRVIMPPMNHVSTIIKKEDIMKDTFKEDMRREVNSFHSACL
FNVAQGPAHVCYKFLMMGYVTNKTDR
>abi19176.t1_1
MEDDQQFCLRWNNHQSTLVAVFDTLLENGTLVDCTLAAEGKHLNAHKVVLSACSPYFESLLSQNYD
KHPIFILKDVKFQELKAMMDYMYRGEVNISQDQLGALLKAAESLQIKGLSDNRKGETETRKAAPPP
PPAKSPPPAATLPRVQGLTIEQRRREMEETREGSQSPGARKKKRIRRRSLEELDNHHDASNSSESH
HSSQTPAQNIPVTLPAASSKITTDVPEPIETKSEILTRHKPMESEVVPQVPIIKEKIETHSELMLE
PKSEYMDEMNEDSIEDLTLDDDDISNMEQMDDQGAGPSHGNMGEGSGQDTKIRRNSFPPDIIRLKR
GKCSDLSRIDLFLDVQKGPFRKYSKTAVRAALSAVLNYGVSGFSGFGGWHMGNQSQDEVFLAAQEA
VGAHRDSQGRIYDPFLKTLLLVCN
>abi19228.t1_1
MKSAFVFVSHDHATAAEEGGRRYEIGNYAKFLKNLHSEQIAKLALKNQHECDLLEDIRSFTIKRSA
IEKSYSEALLKISSAYLNKKIPNIPDIKLDGGEEKWNMWNVWRTVLEENEKLARARLAAVEVFQQQ

IADDAKVLRAHKLTTARKCVEQLAMVQKELQTCVQDVDKTKKFYFDEEHSASDVRDKARDIEEKLK
KKKGSFFQSITSLQKNSAKVSSKREQLEEKSTGARNDYILCLAAANAHQTRYFVVDLQLAMTTMES
NVYEKVAEYLSLMGRTELLTCSAMQNSFSTIRDQAKQLTREYNLQCCYLYYPVLKQHIQYEFEPCD
NDPISVVTAEQETAATTLSKEARRWATKIARENNNIRESVRKLQIYNALRESGQKIDPNDQNGPDL
ETKIEEMKQNIRRAETAKTKAEARIESLRLGGVNVEEWLQEAETLNVQDIQRSTSSLSMRTDASGA
GDHPSSDSFYDSDYAECEPSAHPIEKSDSDDVQRDDIQEVDGEWAMLEQERQRIEQLTADWDDPTQ
VDWGAEDGEHADEISRERSPSGPVLKCTALYSYTAQNPDELTIVENEQLEVVGEGDGDGWLRARNY
RGEEGYVPHNYLDVEREQSSTTPGLVQQFSFSSVDYTVDNEDEGLPQSETNQSPEQVSVIAAVPAE
VVENVSAWSFCCALYDYDGEGEEELTFEEGQVIKVLSKCAHSVDDGWWRGELEGRVGNFPSLVVEE
CDEYGEPLTNQWDETPPCSAPPVFTPPEIPEFLINADLDVDQSTPEKNDEGADTTPSQTTSALANF
AMELTRNQQQQYGTQFQEADVPSKLGSFRKLTNLIQPNLAPFKSLNVKSSTQRLQLTIYRDGREST
GDNSGGDFGLGVAQIVITAATPMIEEAEHPFPSSDETRTNHVEGELEEDARDSPDDETALEQTKDE
INRVVNVTTASVDDFDDRSINGDQADSGHFPVSSSTEGEGETTGPSTAENSISHAPPIEDNEPKQV
VGGRASIPDELEPHQLARLQDLKESNAIMLRFCYPVVYCYVCARDLNDFIFCFHINSRGRDEKKNR
TTKWMVWFYTFRPHDPKKKKISTRGLSGSGMFMHFRSWSQAPYQCKNNFTAFCDCSPSSFSAMSRS
TSPKDSQSDSSNSSPNPIDDLTLYGAPAPLRLKRSATVRRANTVITKREETSSVDDFVLPGDKYRR
SLDSTYRRSLDSISEYKTKISFSATGVHPTRHAGSVDSTTSDCTDYAFVDKNYTHTGSSYLSWIES
VNSEYFACSSSASTDVVDVDNKVGEWNNFWLNYNSPHNRYLSSHCISSHDDKTGDDVSECKSTCST
QREFNEKLASEHVTLTYEEVSEVVHCSQRITEILQRAVLKNENSDRSRNDSYSQHTNSYNDDLAKE
RVPFIDAQDLQKQKQMLKPPTQSTNCINVLLTSGVADILKRVISKRREVIEPPDDIPSTARSSFTD
CKMAATEQFSLRWNNFHSNLTSGFHDLLEAADMVDVTLAVDGHFLQAHKIVLSICSPYFKQLFKIN
PCKHPIVILKDITHGNMKDILEFMYLGEVNVLRENLPSFLRTAELLQVKGLTGDDSSDASSKKDDK
IETSCEADDNTDYTMDADTSLLTQYIPPVPSPPQQKQSQSAVNTKRVIKNSFSNTKRVKSEPCTPP
GKLKNAKISDVPLEPSQIDEDEEFIEVTTKLDQSYDKDDVIKKENYYDGNGTSNLVVGDSNSQDHG
RCR
>abi19687.t1_1
MVTSFRHLRDEKSFTDVTLACEGQTCKAHKMVLSACSPYFKSLLEENPSKHPIIILKDVAYSHLQA
ILEFMYAGEVNVSQEQLPAFLKTADRLKVKGLAEAPQAIKRE
>hom508.t1_1
MISFVIANSIINQRVDMESAQQQQFCLKWNSFGSNLATSLSNLFKCESLADVTLFCDGVTFKAHKL
ILAACSKHLADLFETAPLHQNLLVILDGTSAANMAALLEFMYKGEVHVSQDALSSFLKAAECLQVK
GLSIEHEKLAVAHSSGRVEKHPPLSEEAERPSPYSPMSPYMHPPHYRPYDQKPLDRRPLRSPNEML
QESVRASVLRDGSKAGHPPSPGPMSLAYRPSSSSSCSLPTQQEADAPPEHRFDTEPAATALICPEP
SSKQHCDPGCPEDLRMKMEPIVQDEPPSSTASGNGISSSPSNGPIRHISGYENDRDKTDLIPNTLW
NSVTNKLSHKSSSVSTPDGKKLKCPFCERLYGYETNLRAHIRQRHQGIRVPCPFCSRTFTRNNTVR
RHIAREHKTELSLKAFQQSQQVHNHNPVACMCLWRVRAGTVRLPHQVALVIECKQPSYQWYDSTQE
FITQ
>hom1282.t1_1
MSVVDDQQFCLRWNNFQANITSQFETLRDDEDFTDVTIACDGQRLQAHKVVLSACSPFFKELFKSN
PCPHPIIFMRDVEARHVVALMEFMYAGEVNVAQAHLSAFLRTAESLKIRGLTDTSPSEPFKGREDD
TVFLNPQPSSSKHYLSPSFEKHISNKSQISSTSTVTSSQDISYSSDKPSTPPAKRQCKSERSESEL
SRLRQLSALAEDSITVNPFALQPDIRNSTLEISKSPIHPKLEYLSDDDRDESANFYPSPDATELAG
GMEIIPQISNIMLKENSIETGTGTDSKKLHSLDPRPCEECGRVYSNLSNLRQHMKLIHYPTYVQCT
ICQKDFKTDLYLRRHMLSSHEQSKVGLNEKYVNRPQNRGPIRLRRRRNSDNWLQFDLPISDKSICP
QSDFSSRQRDGREGQLINRLVKLGEGIEIYEDQLRSVKWSDYRKLTRGLATILFSPAELATCSVTG
QRWSRAGSGERPVKPALDRAKVQAIISYVATRFPMVEISRIKQVLAYKCKENSTALKMKAVRYYG
>hom4246.t1_1
MSSQRFCLRWNNHQSNLLSVFDQLLHDESFVDVTLAVEGKLLKAHKMVLSACSPYFQTLFVNHPDK
HPIVILKDVPYNDMKSLLDFMYRGEVSVDQDRLTAFLRVAESLRIKGLTEVNEEKCDSITSSLTQQ
NQHLNSNLSQLQRLQPPTKRFNSLNMLGNALLQPKRKRGRPRKLSGSSNGTPGADDFDRTDNLVQG
SPEMLEVKMGMDGFTGNASDTSSGKNDRDNEMEETSVKEETEPIAGTSKEHNFNSSIAKSNENEFP
PSDDKEKSPNVRCIDFNSSLHEEYNLPYQELLTSDTEVEENDNDLQEIDANYETLDIKVDAKTFIE
NMMDNNSNSNTPKKRKNSSCYDFEEYGNYEIILDDSSFDFRPTKYEKSNRKISDPDKSARDFCVRE
KDNLYRCTVCDRVYTHISNFCRHYMTSHRLDVKMFSCPVCMKDFTRKDNMLAHLKIIHKQQS
>hom4357.t1_1
MTAAKMTMQQYCLKWNNHQPNFISVFSSLLNSESLVDVTLSAEGRHLQAHKLVLSACSSYFQTLFT
INPCQHPIVILKDVKFTDLKVMVDFMYYGEVNVSQEQLPHILKTAEMLKIKGLAEVPVESSISRHN
SSLERTELVTPGESNWVSEPGQRQSMTPSPCPLSPGSRRKRLRKSSTGSGSGSTERTSEEQVGNEI
MLTSPSGTLATVIKPDPGGYVTDTGTTHRIKQHSSDSDVHRSSSHDSVDSEATHQITLQIPQQQDI
NISHNVESSQTMEMPGPSSSTLQVAGFQWGIVDHHTYPSHYVSCQTNVMHNDMQYVTANNQNPSPT

NQPDVKPALNHITNSPPQIMQKRKRSLNPQADENFVRALEAVRFGGIGFCKAARMYGVNNRTLWLE
YKKRGYPISRLSIKNRKPENTQQQQIQQPPSPPNQQPQENEVVVSTVNNPIAMISSAFLEGRPVEI
APVLQRSRFFDNGIMNGQPMNLQGLNFEPM
>hom6368.t1_1
MAGEQFSLCWDNFHKNMSSGMHSLLESGDLVDVTLAVEGKYLKAHKMVLSVCSPYFRELFQNNPCK
HPIMFMKDVSYIAMSDLLTFMYQGEVQVNQENLATFIKTAEALQIKGLTGDGNGEISDVSEEIQIE
KEEMQLEKPTKRTIEVSAEISKSTQRPRKLSSGVKRPKLSTPVQDVDVSKEDAMTVTPSVQFKMEP
YESVDQTNIENIDDSQAETFGDETLDDSHMEDTEDYSMLEGEDPQVGTSGDGMGDSQEFTASKRPE
TKHKKLNSGIVVDGFVYHQNSTKNSTQMRYLVCSEYKRLVCRARAMLPKDGTSHELILTRGHNHPP
IWNAEEKAMFLRCLKDFVETSPSLSLRKAYESTQIL
>hom15864.t1_1
MEGEQFSLCWNNFHNNLSSGFHTLLQDEDLVDVTLAAEGKFLKAHKTVLSVCSPYFKELFRMNPCK
HPIVILQDVSYSALHNLLQFMYQGEVSVSQEEIPTFMRVAETLRVKGLTDNSSNPELNGLDNSPMN
SPQTYGSLPDSKQFVKRRPIKKVLKPIQHLKPIESPRSMKSPPIARSSPYVPVKQPRLEEPAIPTP
EYGHSVVNPKQEPIDPNDDEFKDQSYSEHMDMSSMLDTTLGESSDSKSHLMSPSHKVPTDTSSPGK
LIFFLIMVFVLA
>hom16266.t1_1
MGGDTSPEQQYALRWNDFHSSILSSFRHLRDEEDFVDVTLACDGCSFTAHKVVLSACSPYFRRLLK
ANPCQHPIVILRDVQQKDMENLLRFMYNGEVHIGQEHLADFLKTAQMLQVRGLADVPAGSAGQKLT
VPEQKVSPIKSKNIEINLQNSSSLPWATDRSDALRDSGLSPPPLKRSRSTENSDSYIPDRVRSASR
GNIDMQESLLGQALEGGPTIHTNAKGNSDSPPRAQSTGDDSSSDTQMSDHERDPITPKTEPTDYPM
MDDHPFSTNGGLIDQSRTPSFPGALINLPGRIDPFYVFYSLNVPHPFHLFYIYAEGI
>hom16927.t1_1
MGSTAQQYCLRWNNHRSNLLTVFDELLQNEAFTDVTLVCDGGGPIKCHRMVLAACSPYFQNLFTDL
PCKHPVVVLKDVKYCEIKAILEYMYRGEVNVAQDQLAALLKVAEALKVKGLVEENRTNEQNNMTRR
DDEPMEHSPSGITTSTTNTHSAVHNSSSNTSPPHSTNGVLYSKNPYNLYGKAPGIERNSRMNIPMW
AVPGIPLPHHPSPVPPQNHPHTAATAAAVMLSSCYEAASSDMSPLRRKKLSSLLMNRDTPILRTVL
GQGQADSSQPVSLVCHPDNHHENVNTNAPDERVIRHMKNEPSEDAQSPYTDLSMMEDEEKSKMGIP
SSSPQSYSGEMRSISSGIATYVPNQKPEWKRYKQYTRNDIMSAIEAVRNGMSALQAARKYGVPSRT
LYDKVKKLGITTSRPFKRGSNGSTACFPYGLSGTSSPYENEEHGMNNNSALLEASSFLQHALDGRG
GDEREALAAMAAAAAAHAAATGHSTSPPNHGMARSPSPSPSFIKYMRQSSLTPSPAPCSEHLHSET
NGSSERDRDDDDDQVEDLSIGRKQESRVIMPPMNHVSTIIKKEDIMKDTFKEDMRREVSVEEAE
>hom17053.t1_1
MEDDQQFCLRWNNHQSTLVAVFDTLLENGTLVDCTLAAEGKHLNAHKVVLSACSPYFESLLSQNYD
KHPIFILKDVKFQELKAMMDYMYRGEVNISQDQLGALLKAAESLQIKGLSDNRKGETETRKAAPPP
PPAKSPPPAATLPRVQGLTIEQRRREMEETREGSQSPGARKKKRIRRRSLEELDNHHDASNSSESH
HSSQTPAQNIPVTLPAASSKITTDVPEPIETKSEILTRHKPMESEVVPQVPIIKEKIETHSELMLE
PKSEYMDEMNEDSIEDLTLDDDDISNMEQMDDQGAGPSHGNMGEGSGQGFGGWHMGNQSQDEVFLA
AQEAVGAHRDSQGRIYDPFLKTLLLVCN
>hom17094.t1_1
MAATEQFSLRWNNFHSNLTSGFHDLLEAADMVDVTLAVDGHFLQAHKIVLSICSPYFKQLFKINPC
KHPIVILKDITHGNMKDILEFMYLGEVNVLRENLPSFLRTAELLQVKGLTGDDSSDASSKKDDKIE
TSCEADDNTDYTMDADTSLLTQYIPPVPSPPQQKQSQSAVNTKRVIKNSFSNTKRVKSEPCTPPGK
LKNAKISDVPLEPSQIDEDEEFIEVTTKLDQSYDKDDVIKKENYYDGNGTSNLVVGDSNSQDHGRC
R
>hom17496.t1_1
MADQQQFFLKWNDFQSNMVTSFRHLRDEKSFTDVTLACEGQTCKAHKMVLSACSPYFKSLLEENPS
KHPIIILKDVAYSHLQAILEFMYAGEVNVSQEQLPAFLKTADRLKVKGLAEAPQAIKRE

Sex peptide receptor

>abi2267.t1_1
MTNASIKLLNVSSVIYNETYYEPCTEHPPIVLYWFVTNGVVMNILGLFGIIGNIISMIILSRPQMR
SSINYFLIVLARIDTVLIITSILLFGLASVYPYTGYMYTFYYNVHPHITPWVYPLAMVAQIASAYT
TVMVSMDRFVAVCLPLRARSLCTYGRARIYVIGILVFSLLYNIPRWWEITIQSDWHPETNITVYCV
RPTPLRNNETYLIVYVNWCYLIFVYLIPFLSLTTLNMAIYRQPGEENVDQRNRATTLYLPYMKQDI
ERTLASWTTGTPVNTLLKTGETGKMVTHLPISPANSILLKMSRIAAVACFQKPNFAVSYTLFITGP
TRVRKANKERQRLSRVQRREIGLATMLLCVVIVFFACNFLSMMVNITECFPSLVDPIIYDHMVKTS
NLLVTINSSVNFLIYVTFGEKFKRLFLVLFCSHGFFSTSGGRDSPEGATTHDDSFISNGGGDRQSL
RLHRHNTNISRNGTTIRLNGTVRENRKNRSRASSPSPCVYYPANRTNKDTNAVAYTTQMSVPSTDW
ERMSTSSDF
>abi4258.t1_1
MNLDTQHLEDIKRFAQKYEDIVHKQENADLHQHWPQSSHRKSRAKCSQNETHINLQSSSLRDTYDQ
DGTNQKRSSATENIKPKMNTTASSILMTNVTNSATMSSATAAIKYCDLNEFSKIYRQVHGYTSVLV
CVFGSVANILNICVLTTKEMRWPTNFILTGLAVADLLVMLEYIPFASHVYFNPKSRLHASYFTYSW
AVYMIFHALFTQVCHFISCCLTVMLAIWRYIAITHPQNNRLWYTVSNVQSTIMAIVITYIFCPLVC
FPIFLSLNVITRTHLLDENNTLISAKQRKNFTGILHNGTLYTAELSGDFINISLWVYGVVIKLVPC
VLLTVLSTRLISALLETKKRRRNLLNNSGVPLQECKGKIVKTNKKHLEKEQQTDRTTRMLLAVLLL
FLITEFPQAILGLLSVIIGGAFVKQCYAPLX
>abi11982.t1_1
MDENNRSSNDVPIDGVPSPATILYANGTSLPNATDITYINVTQELPINYAVPMYGYVMPFLLLITI
IANTLIVVVLSKRHMRTPTNVVLMAMALCDMFTLLFPAPWLIYMYTFGNHYKPLWPISVCYIWFVM
HEVIPNMFHTASIWLTLALAVQRYIYVCHAPLARKFCTMPNVYKCLGYIMLTAALHQSTRLTEQDY
GERPTLSTFLKNVLPSFGENSKGNDNLLLSNQIIKLSAIAFICFRLIFHITLSYALDTIQLNKY
>abi20991.t1_1
MSSSVLVYPLFLEEEDLLLMKIEWNNETVYVCTRTTAAWVKNYITEDVYYMSYYMFRIICVHLLPC
IALVVLNVLLFRALKQAQKRRDILLAKKNQKTECKKLRDSNCTTLMLIVVVTVFLIVEIPLAVVTL
LHVLSSSIFSEFLDYHIANVLILFTNFFIILSYPINFAIYCGMSRQFRETFKELFIRGAVTNRNGG
SSRYSLVNGPRTCLRALRLKGSGGGSVRLLNHKALLGINSRNTINLNIMIGYIITDDHRIDHRGRL
GISFTIVIGTIHIVMVIT
>hom2024.t1_1
MTNASIKLLNVSSVIYNETYYEPCTEHPPIVLYWFVTNGVVMNILGLFGIIGNIISMIILSRPQMR
SSINYFLIVLARIDTVLIITSILLFGLASVYPYTGYMYTFYYNVHPHITPWVYPLAMVAQIASAYT
TVMVSMDRFVAVCLPLRARSLCTYGRARIYVIGILVFSLLYNIPRWWEITIQSDWHPETNITVYCV
RPTPLRNNETYLIVYVNWCYLIFVYLIPFLSLTTLNMAIYRQLASWTTGTPVNTLLKTGETGKMVT
HLPISPANSILLKMSRIAAVACFQKPNFAVSYTLFITGPTRVRKANKERQRLSRVQRREIGLATML
LCVVIVFFACNFLSMMVNITECFPSLVDPIIYDHMVKTSNLLVTINSSVNFLIYVTFGEKFKRLFL
VLFCSHGFFSTSGGRDSPEGATTHDDSFISNGGGDRQSLRLHRHNTNISRNGTTIRLNGTVRENRK
NRSRASSPSPCVYYPANRTNKDTNAVAYTTQMSVPSTDWERMSTSSDF
>hom3788.t1_1
MNTTASSILMTNVTNSATMSSATAAIKYCDLNEFSKIYRQVHGYTSVLVCVFGSVANILNICVLTT
KEMRWPTNFILTGLAVADLLVMLEYIPFASHVYFNPKSRLHASYFTYSWAVYMIFHALFTQVCHFI
SCCLTVMLAIWRYIAITHPQNNRLWYTVSNVQSTIMAIVITYIFCPLVCFPIFLSLNVITRTHLLD
ENNTLISAKQRKNFTGILHNGTLYTAELSGDFINISLWVYGVVIKLVPCVLLTVLSTRLISALLET
KKRRRNLLNNSGVPLQECKGKIVKTNKKHLEKEQQTDRTTRMLLAVLLLFLITEFPQAILGLLSVI
IGGAFVKQCYAPLX
>hom18604.t1_1
MKIEWNNETVYVCTRTTAAWVKNYITEDVYYMSYYMFRIICVHLLPCIALVVLNVLLFRALKQAQK
RRDILLAKKNQKTECKKLRDSNCTTLMLIVVVTVFLIVEIPLAVVTLLHVLSSSIFSEFLDYHIAN
VLILFTNFFIILSYPINFAIYCGMSRQFRETFKELFIRGAVTNRNGGSSRYSLVNGPRTCTNETVL

Apolipophorin-III

```
>abi9089.t1_1
MNICLPDRINFKLSKNSHVYGDKIQVPIVAETMAYSYLTTTKRRGRIEVIVVTVRFAENLCEIHPM
IMWSHEYEAARKGPWEEYARDRDRFRRKIADVEENLGGIFDAEHRRRIYEERFADRVFECRLVTAH
QGEVDNVVKQVEKKLEETVTALQKAAGPEATAKGQELKTKFDEGFKSAVSEIEKLVKSVQPGVEDA
QKNLKNLTKTALDQILDFGNTVQSQVNSAIADHEKTHKH
>abi11323.t1_1
MAKTFSILLLTFLILQNSTSKPVEKSSLEILAENAKAVAENVTATLGLQDINSQKVVKTINQQTQL
LADRLQTIGDKLKGEIKAHQGEMDNVVKQVQAKLSETAESLKKIAGPQNTAKFEELKTKFDDGFKN
AIVEIEKLAKSAQPKADEVQKNIKDLAKKAYDDVLEIGRNIQTQVNIAIEKHEKTHKQTRKSLVCG
GVNSPQMRVIIPNPLCTDRPARLHKLRDVFINHLCGVSSIIT
>hom8095.t1_1
MIMWSHEYEAARKGPWEEYARDRDRFRRKIADVEENLGGIFDAEHRRRIYEERFADRVFECRLVTA
HQGEVDNVVKQVEKKLEETVTALQKAAGPEATAKGQELKTKFDEGFKSAVSEIEKLVKSVQPGVED
AQKNLKNLTKTALDQILDFGNTVQSQVNSAIADHEKTHKH
```

Octopamine receptor

>abi159.t1_1
MALDFDLLTTLGLVTSSLIAVISNLLLLLVFFRRRGLRTVSNRFVINLLVTNLLSSVFLVPLLIVD
RESESPLGKNELYTNISTSELDFRNGSSVPRKNATKVFFHEENVFDGSDFFESEFVLRDATLEITE
IFNLRGNFTDNFLCFFAQSSTNLICTASILSILLIGIDQFFAVVHPLRYHSFIDKCKSTIFICVCW
CISVILASFDALISVDANFWTFCSDRTVIIETDDDDLQVFRIVYSIVYFIFVVLMPFLLICGIYVC
IYTAARKNSERMRKSTSANSTVNLNSYSQLSVQNESNDVSLPKIHSSPNLTDLQRINDNKIARSVS
ERTGKNIITSLKSRISNASVFKYREETRAAKISILVIFMVLICYGPYGLAVLFNTHVYPAKMPHFY
NYLSLVLLVASNMASPFIFAYRNRRIQREVLKFLHLIPRKNNVSIRRNFREEMLQTRRESDERDNR
VLKDVEMSCCKEENEKKGILKRVCINFSDLEEALLLLPFPAVCEMLQRLTPLVIRGDHTELISKLT
LFLLKIHHAPIVANSSLLPVLKQLKKSMTVKVE
>abi647.t1_1
MGDRGILYDSTTSSSDESVSFATDESFFNAMTENFTYVNVTSQQKTQLMLYDVLIPTIGTLSIVMN
LAVVISSGLILKKGQQPRSTYLFLGNVAMTDLITSIAIVFGQTFPKNLRDRHICAIQMGMIVASTL
ASVYSVGLIAVDRFLYIIYGLQYQRWIYPLRARLLILSTWIIGCTIGFLPLMGWAGDTQNGKICWF
IILAPKELILLTVSIGALPLILVVVLYSIILYHAIKKIIQLQKAHEADVHNKVETDSKGLRIFRGR
GAQSDTSDVEDHSATPREEKSLFKRIFTRKPQNNLNNPSKWKAIKVVLFTSGSFVATWSPYFIACV
VYIYCGDPQTQKCKTLKILIASPLAILGFVNSLVNPIIYAWWHKGFRSFVTQKFNSIRKKKISISN
TSSTDTKKTSNASNSDQKKSIRSMPLSNSTEHIIDENGSKTDSSSKSETTGETKLSNTTLDNDERE
KSDKKFIVSKSDYAGVHNSRENIVKVEENGSTDL
>abi681.t1_1
MASVGCAGNLVVLLGRLLAPTNNVVHSLYIKNLALSDLLMGIYLFTIAGADQHYRGVYIQHDYIWR
HSRLCNLCGFLSTLSCESSVLILSLVTWDRFISVTQPLARKQPSPKTAALTLLLLWLLAATVAFIP
LTELTAAYFKEFYNSNGVCLPLHIHDPFQEGWEYSIAMFVFLNAFALLFISYAYTRMIQEIRASGV
ACRSTQQSHDRDKVAQRFGIIVLTDCLCWVPVIVVKIAALLGTHISEYLYAWLAIFVLPINSALNP
VLYTLTTTMFKKQVRVEPILGPSPPHGSQCKADDIQSEPIKAEQSREVEVVPESANPIRKSIGSCV
RKRRRRPEHQISGYDSAFSLSFGMFPLVGSTRRGLNYRVKESIQNVFYSSIEFQGTQSSLANSKDS
WRRPTAV
>abi755.t1_1
MNVPMENETRIDGFDNSTFDRDENLAKIEIATLAVIFLVTILGNAMVLLALWTRRRYAGRKKLSRM
YFFILHLSIADLITAFLSVLPQLAWDITYRFSGGFLLCKVVKYGQTLGPYLSSYVLMATAIDRHQA
ICYPLTYCSWTSRRSKFMVWTAWATSLIFCIPQLMIFAYQEVDAGIWDCWATFSEPWGQKAYVTWY
GISVFIVPLIVLVVTYSWICREIWHSAVGELGTKPRYVSKLSPEKKIPLISRAKINTVKQTIAVIV
MYTVCSTPFIFAQLWATWDPNAHLLPFFNGPIFTILTLLYSLNSCVNPWIYLAFNRELPRLLLRHY
TATSNNYRAAAG
>abi865.t1_1
MVSMLNQAFWESFKRFEIIDQSTYFALKRRYTWRKLLNATRRPATPQHAHSAASTDTDLTTIDTHE
LWIQESEPSPCTMSALGQFGAEMLKLSRGLEAASTNSAKSRNCQLNSHESTLQIGSKESNSSSIFD
SNSPLLTTESSMSPWSMKRRRASTFHEGDTRDESPSPRWRKRRTSFHERRPTDMSNESKSEGTECG
TLPLLSPYVCSYFNEKSENSSPSRSSEVKIIQNEQRSSKLSLDMEEPIPTISPIKRTQLNKNRNND
QSANNIVVTWECPRRNHRRGSSFGSTRTQVLNSNQRTPLLLRRSATLRHNGHGATGLQDKNKNPSS
PCLLQRYNYNNTSTTRSNAIRSHHSRNSSVISRNSSRHGRIIRLEQKATKVLGVVFFTFVILWAPF
FVLNLLPIVCEHCEKNIGSWVFQFVTWLGYASSMVNPIFYTIFNKVFRQAFKKVLLCRYRNQTWRP
HR
>abi1314.t1_1
MEPNASIQSKTNFTVYSSDDEPLFTPFEATLITLALSLVIFGTIVGNILVCVAVCLVRKLRRPCNY
LLVSLAVSDLCVAILVMPMAMIYEITGTWNFGQIACNLWVSFDVLSCTASILNLCMVSVDRYNAIT
KPLEYGVKRTPKRMIIYVILVWCGAACISLPPLLILGNTHDMEKNSKKLTVCIVCQAIGYQIYATM
CSFYIPLTVMMIVYYKIFRAARRIVLEEKRAQSHLDAHCYLEISVKNGDGPPENKISNLSPSPANT
ARINHRCSTASTNTTKDKITAFMKKLELWIISLKNNNFDIFPTFKITCLADEIEESKVLINNHLTS
LWKQFSFYFKDLDMSKYEWIRNPFVIEKYDDFGLTTEEQEMIIDLSSDSTLKQMFQDENNIVTFWL
R
>abi1660.t1_1
MDNIDIANITTLNTTKDTKTEEWTDFLLLFVRTTIMGSIIIASIFGNLLVIISVMRHRKLRVITNY
FVVSLALADMLVAMFAMTFNASVQIFDRWLFSYFMCDVWNSLDVYFSTASILHLCCISVDRYYAIV
RPLKYPISMTKKVVAIMLLNTWISPAIISFVPIFMGWYTTDENQDFRNKHQDVCEFVVNKYYAVIS
SSISFWIPCTIMIFTYLAIFREANKQEKELYSRHGAALLLHQNNTNGDMLSNSGGSSKTLMHEINQ
DLHHTPTKERNIIKMKREHKAARTLGIIMGTFILCWLPFFLCIVVTIVFWIGYFNSTLNPIIYAYF

NREFREAFKNTLQCLFCSLCTRPPSDLDHYDVRRPSLRYDDRTRSIYSETYLKHIDRRRSSEFGSS
L
>abi1928.t1_1
MRDSCRGIGYLRKGEEMRVKGVSTHARRRERLIQCAVVERGGNMPSKLRVDSGAEDDEDDAASVYD
YYRFDHLVLCVSHLSPTRAASVKRVLASVMALANVTARSAVGVEDILIMLDNGTSNGTFSNGTVTI
TTSYPSGYTLPQIVLASIIVTILMIVVVIGNMLVIIAITTEKALKNIQNWFIASLAVADFFLGLVI
MPFSLANELMGYWIFGAWWCDVHSAMDVLLSTASIMNLCLISLDRYWSITQAVEYLKKRTPMRAVV
MIGAVWLLSALICIPPLLGWKVARTPDEQFPKCQ
>abi3436.t1_1
MQKEISAGLNTTNDTTIHIEELALAGLLSIVIVITVIGNTLVILAVLTTRRLRTVTNCFVMSVAIA
DWLVGIFVMPPAVAYRLMGKWELGWILCDIWVSLDILLCTASILSLCAISVDRYLAVTQPLSYSRK
RRSKRLAFSMILVVWIISALITCPPMFGWYIKPKGELI
>abi6780.t1_1
MAGWNDTFLFDNITFINETFELLQEVQKIDALYNESSSEAMYPLGWVIPVTVVYAIIFLTGLLGNV
STCIVIARNKTMHTATNYYLFSLAISDLLLLISGLPPEMYRIWSPDVYVFGQAFCVLQGFAAETSA
NATVLTITAFTVERYVAICHPFLSHTIAKLSRIIKFIVAIWVVALCLAIPQAIQFGVYYDEEDGVQ
SSTCTVITENSFSHAFEISTFVFFIGPMTLITVLYVLIAFKLSKSRLLVNAESTSLESRCNLQSRI
SRNSRSSAAQKRVIKMLELRILFEIISDRMIIHKCYIKKTKQKNQKKVAIFS
>abi6901.t1_1
ILGSNERRLHTEQHANKSDDHRRVERGVGRFTRTANSTIRMERSRVLASHQRKETMSGKPRYRLPD
IRNVLYFLRSFACHFRALLENLPNGAKTYPKTTRTEESKPPKVFKNEQQQMKKCANKKSSAAEALV
ASLVMMEGQSTTTMEIVEEQDEQQNGVEQEEETTAFTISKPKCQHNTTQVHIAPISNNVSPEKSST
ATTNNGSASHQSHISDITRVEILTKNIPVCPASVDKLLPTRKDKKESMEAKRERKAAKTLAIITGA
FVMCWLPFFIFALVMPLCEDCIHPYVMSFFLWWGYFNSTLNPVIYTIFNPEFRQAFKRILCGVHGR
GNRNFRSGKIR
>abi7062.t1_1
MSTLPFQVVLFVIVCLVGLLGNTLVIYVVIRFSKMQTVTNMYIVNLAIADECFLIGIPFLIVTMIR
RSWIFGEIMCKAYMTMTSVNQFTSSTFLFVMSADRYIAVCHPISSPKWRTPLISRIVSFAAWTLSI
TLMTPIIINSHLVESNSENSTSGSCVITFSNETYHQTARAFTLYAFVFGFGTPLCFILIFYCLVIL
KLKTVGPQNKSKEKRRSHRKVTNLVLTVVTVYVICWLPYWITQLATTYTSDEVTIYFNNCFPFAWK
TVDSTAKKSRSWSRRIWMWKQFLALIGDAFDNTTLNMWTSIIMVENDAVPFAWSFRLDCSGRVHRH
HC
>abi7342.t1_1
MNDDELSVASSILGNKTHPQGKQTPTKWLRRAKMETTKRYETETVFSNETIVWSVVDLTLLLITVA
GNALTLCAIILSKKVSGLLSNRFVFSLALSDLMVGLTIPYHFAFSVGHTLSENRETCILRFVLVIF
ACSSSIYNLLAIATDRYLAIVHPLRYGRYMQKRIAYAVIIFGWCVTLFVATVVIYWNRWAKGKECS
LNHVVPAEYVTYVLIPMFISVWFVMLLIYIKIWRVANKHAKRLRDVTKSHNGTMLPKDWKSVKVVM
LILGCFSICWMPYFATIAYIRIFGHIPTLLYEITFTLAMANSSMNPVIYAWKNSNFRKSFWCMLRC
KSPNSLVEKPQYVTNHVPTIDRNVARQEHDENNVTIYSKLTFEIDDETNDDRKSDTTSADTISR
>abi7411.t1_1
XMADENGSCPVEDIEFTNISLGFKSAVPEWEALLTILTLGLIIIILTVIGNILVILSVFTHKPLRIV
QNFFIVSLAVADLTVAILVMPLNVAYSILGRWLFGIHICKMWLTSDVMCCTASILNLCAIALDRFW
AITDPINYAQKRTLKRVLLMIGGVWVLSLIISSPPLIGWNDWPDPDTFLIQERCQLTERQGYVVYS
SLGSFFIPLFIMTIVYVEIYIATRRRLRERAQASKINAFMSKNTCHNKGTPDRESVSSETNHNENP
NPCEVPVNGKSKDKKHKTKRKKKNGDGEKSQFLKPMLVQEDSVTDNADAHRNNSLPSTSDNKDTSC
INTSLISNPQPKKINQSEKKPGVVYQFIEEKQRISLSKERRAARTLGIIMGVFVVCWLPFFLMYVI
KPFCKNCCPSARLINFITWLGYVNSALNPIIYTIFNLDFRRAFKKLLGMKP
>abi9232.t1_1
XTVSMPFYTVYVLMGYWDLGPLLCDLWLSVDYTVCLVSQYTVLLITIDRFCSVKIAAKYRSWRTKN
RVVWMVTITWIVPALLFFISIFGWEHFIGYRDLLPGQCAVQFLKDPIFNTALIIGYYWTTLIVLFI
LYGGIYKTAYDMQKKSEAKQRKMQSMVALSAGTMTGMAGRAAGIGLSKTQSTLLSQDKPPVVPSSG
LPPSQTNNTTNVVNLTIREQNPGMGSADSNSSQKTCSIKNSTTTTTTTTTADTSNNDKRNLNPATH
TTTEGDKSERSSSPAFESDDESAAVQQNRKRSSVVGIVVQIGMSPNLFDNNNRINGGIPTSGVELT
NTRPSNSGGDVISPSLPLILEQSLLDTDNPTENTGVGDLCSIEPLKPEPALNVVDKLPAEPCCSPS
LSQKSQNIIPPPVQFQETPLSNSPPSVAERPKTLQVKPQVTYDVLIGLDGGDLRYMDESSVNVPSP
LCESPPSSSTISESPTHVTNTAANTSLLQTALIRATAQANPPSKKLLNTEVTLTSERPITTIVNEP
PSNCQRDINTEPSTPLCGMPTISQTGNAAAIMAATTIVDVDRNKSLNKTDTDDGLLKKDFVKNIGR
RLKTKKKKTDSTISVRQKSKSENRARKAFRTISFILGAFVVCWTPYHILALVEGFCSDPPCTNGHL
YMFSYFLCYANSPLNPFCYALANQQFKKTFTRILKGDLHIT
>abi9293.t1_1

METSSTTIYNHSHQALVLYAIEMQPLDNISDVDVNVTRNMSRLNSPLSVTVAVLLVFLFSPMILIG
NSLVLVAMYRFKRLRTPSNYLVMSLATSDLGIGMFMPVGMYLELGGMEKFTGARVCLLSYGIATTL
CCVSVLVMVAIAVDRFTSLARPLRYNNLITHSAIERYIIVFWIYASLVGFTPLGYSILHGNNVX
>abi10261.t1_1
MNEIVIIPDLVTNLEENLTKDLENFSDKTEFAHPNKSIYTYSTNVDIGNNFASRTWILWLLVILKT
TVMALIIVAALFGNLLVIVSVMRHRKLRVITNYFVVSLALADMLVAIWAMCFNFSVEITGGIWLFG
YFMCDVWNSLDVYFSTASILHLCCISVDRYYAIVQPLDYPLIMTNVKLAIMLGVVWCSPALVSFLP
IFMEWYTTEENLIFRKKHPHVCNFEVNKTYSVISSSVSFWVPGMVMIFMYYRIYVEADRQERMLYS
KRKRKGNGTSGRTGKSPRIPNGKKDFTSEQEEIDIFQWRIRFSINDDDGDLYEIVYFT
>abi11353.t1_1
MVIFTLCVAIVRTKTTNVRDPNVTSSNFTCNDHDPNPFLTNQFSNNLCPPKKSTNEARNDKLNNVV
ASNVVENLTERTSKLNQPPFNRKATTDRLENGLLSAVARGVPLAKRNTNERMNFSTTNETDSNGTL
IDEEYINTIEEFIYPKSWTWVLIFFHSMVFILGLVGNTLVCVAVYRNHTMRTVTNYFITNLAVADF
LVILLCLPPTLIWDVTMTWFFGMTMCKIVLYSQ
>abi11530.t1_1
MNIYLNPCEITQILCKHCRNLETWNFISRSNFRNGNPPDVGSAVQLSKLRYPSKVAAALLNKHLQI
NGISAGLTTLRESVDADFAEAKAEAGSSSKMKRERKAARTLGIIVSAFLACWLPFFLWYVITSLCG
SELCYNPPAVVTLVFWIGYFNSALNPLIYAYFNREFRVAFKKTLQNCCRFTSKIMCWKCRSSRDQI
TYSNASSEIHMNNHLRISDQRGEGNQRLSYNISEGEIINLQSEAVI
>abi11532.t1_1
CSMEKSGLSRCFKSRRSNESQCPMLSSKPQIKEPSQQKQQRSSLLTVKTSPSTTSTQKKLRFQLAK
ERKASTTLGIIMSAFTICWLPFFVLAPVRPFLDPSSSTVIKLSSVFLWLGYANSLLNPIIYATLNR
DFRKPFQEILYFRCGSLNHMMREEFYHSQYGDPDQHVSSRHMDYDEGVEYIEAGGEETKVVEIDAA
HESFL
>abi12270.t1_1
MDVNKGLGHVHLYLMLSPLALRFRTNTINKNEKIKENQNKFEQRVSMASPGSVDAFPKIRRKRRNR
VDRRQTQNVENMIVNAMASFSATFTTNPPILLHNANNLQNRTNITNIENLHPDWTDLLLLVVKSCI
FGTIILSAVLGNALVIISVHRHRKLRVITNYYVVSLAMADMLVALCAMTFNASVELTGGKWLFGYF
MCDVWNSLDVYFSTASILHLCCISVDRYYAIVRPLEYPITMTTKTVSFMLANVWILPALISFTPIF
LGWYTTEDHKVYRSENPNVCIFVVNKYYAIISSSISFWIPGIVMITMYCRIYKEAIRQRKALSRTS
SNIILNSIHQHRTSTYNTRYGDHYLHPSDGELTTIGQINGRRSTSSGSAISYGTTTNMDNCKAATE
LNMNAFIRKDLNSLLNERGEGNLVEHKRCELSPSDTRNEESSKRSLMYDVRCTMYTMNNIGPSMDP
CGTPIFIVRLYEAHENGHEFSLKNSKNWRVKTTP
>abi13002.t1_1
MDTIIPSPSCGVRALGNRFDEQRLTLRIHRGRGSSGRRHHGSPHSNGSNSTITTESASPSPRGKHE
RVKISVSYPSSDNLSPHPAPHLLAITPTSPTGQPTYTVHYTVNGKENTSTNLCRRDKHLRVSQRLA
AHRRNSCDSNEDRMQRSPSPSVCDDGINKPKIISKRMGKRNIKAQVKRFRMETKAAKTLGIIRHCS
SASRRGSDGSQLQGRQFRSPSFNIQQPGNSLGEDSDPGGDPSDSX
>abi13491.t1_1
MIVNKSSVQVDGNNTSLDIIDDTVVQALFLMLYTSIFILGIFGNVLVCYVVFRNKAMQTVTNLFIT
NLALSDILLCVLAVPFTPLYTFLGKWIFGSVICHLVSYAQGASVYISTLTLTSIAIDRFFVIIYPF
HPRMKLSTCIFIIINIWLFSILVTLPYGIYMLHTHDNKTSEAKYFCEENWPSEKYRRIFGGLTTLM
QFVIPFFLIAFCYICVSLRLNDRAKSKPGSKNSRKEEADRERKRRTNRMLIAMVVIFLLSWLPLNT
INVINDFSIHLGKWQYYLLSFFMVHAMAMSSTCYNPFLYAWLNENFRKEFKQVLPCFERAARTTAS
GRIGNWRTERTCNGNNETQQESLLPSGIHRATSVRERKTTPSQPTKTDSVEVENVLVPTVGAVYDS
VAETVRLKLISEEDPPPYEATIKNDL
>abi14118.t1_1
MNTSSPLNVTTESIYSITEVVFIGTIAAVLSVMTVVGNIMVMISFKIDKQLQTVSNYFLFSLAVAD
FAIGLISMPLFTVYTLLGYWPLGTLICDTWLALDYLASNASVLNLLIISFDRYFSVTRPLTYRAKR
TTKRVAIMIGCAWGISLLLWPPWIYSWPYIEGERTVPDKACYIQFIETNHYITFGTAIAAFYVPVT
VMCILYWRIWRETEKRQKDLPNLQAGKKDSSKRSNSSVYVCSDEANTACSTVDLEEWRRPRSESSG
ADTDSVYMHSSVCADHHAHRKRPVSIRTIFVH
>abi15910.t1_1
MFQLTTDNTYGVNRTSNLKYVELNDTYYNTDLIQALQTETFANTQNSSSWGPKRDPLYIVIPITIL
YAIIFFSGVVGNVSTCIVIARNKCMHTATNYYLFSLAISDLLLLVSGLPPEICQIWSKYPYIFGEA
FCVVQGFAAETSANATVLTITAFTVERYVAICHPFLSHTMSKLSRAIKFVIGVWILALCLAIPQAI
SFGIVYERSPDGRMMNDEHYVCNVKRIVIPHTFAISTLLHRSTVSSSRCNSVKLKHRVCRPTITHT
NISTQAVLVMNGDNIEGVQHNRPEDEGRKNFARNSQATKHVVKMLGE
>abi15981.t1_1

MILLNGTAPPNIRPQARVDTTGGRRMLGWNVPPEDLIHIPEHWLVYPEPEASIHYLLGIIYVMFFI
MSVIGNGLVLWIFSAAKKLRTPSNVFVVNLAFCDFIMMLKSPIFIYNSFSRGFACGPIACQVFAFV
GSLSGIGASMTNTCIAYDRYTTISNPLEGKLTKIKALVMILCVWAYTIPWAVLPLLEIWGRFVPEG
FLTSCTFDYFMDTFDNRMFVGVLFTFSYLIPMSLIIYFYSQIVGHVVGHEKALREQAKKMNVESLR
SNANQASQSAEVRIAKAAITICFLFVASWTPYAVMALIGSFGDASLLTPGVTMIPALTCKLVACID
PYVYAISHPRYRIELQNRLPWLAIKEDGETTSVGTEHTTAAPAATTPATS
>abi16583.t1_1
MDRLTCVEAEPSNASKITLVSLSSHKLFRAEEDIFGLNFAVRFLMKTSSRVIKQREKSHCIKMHKD
NLPLKPTTPGQHSPRITKYYGTRTVEKDFTNVSIAESILVIRKKLVEDQELQERTSIPINGILELL
RRCLKTTFQKFPKRSAVLLIRKTPSSGIPTNPNSFDERSAQSSFRGSTLSVNGELQQQAAALARNR
QPSVGIDMDMVSEFDPSSSDSGVVSRCTVVKPLKLRLCKPIFGKRTTKNRHAEESRAGSSCEPIVE
KVVPRIQKPRDPEREKRRIARKKEKRATLILGLIMGSFIACWLPFFFMYILRLAFDMPGIAFSTAF
WLGYMNSALNPVIYTIFNKDFRRAFRRILFK
>abi16595.t1_1
MNISDYFYITSYDMANESLDFNWTNATILEMDAWKHWESLVQKRAVMVSLLLLFSMATVFGNTLVI
LAVVRERYLHTATNYFITSLAVADCLVGLVVMPFSALYEVLEHTWFFGPDLCDVWRSLDVLFSTAS
ILNLCVISLDRYWAITDPISYPMRMTRSRAILLIAAVWVCSSGISFPAIAWWRAVRTEPIPEYKCP
FTEHLGYLIFSSTISFYLPLFVMVFTYYRIYRAAVAQTKSLRLGTKQVLLGAGSLELTLRIHRGGT
GKLATPETRNLYANQEDEPLTALQNNGLARMASTRLGPHNKNFSLSRKLAKFAKEKKAAKTLGIVM
GVFIICWLPFFVVNLLSGFCLQCIWHEEIVLAVVTWLGWINSSMNPVIYACWSRDFRSDGNINAHL
LYEFPVAYEETLDVNNSCNRER
>abi16849.t1_1
MEQNDEDFMMDPMEQYNTSYNASVTNSTTRYTPYVERPETYFVPVLFFMIFVVGVLGNGTLVVIFL
RHRTMRNVPNTYILSLALGDLLVLITSVPFTSVVYTVESWPWGELICKLSETAQDISIGVSVFTLT
ALSADRFFAIVDPLKKFHTSGSGRKATRITLGIAISIWISAIICAIPSAVGSHLVVKGYDSFQFTI
CYPFPETWFNKKYPKINVMTKFLILYVVPLTIIAIFYMNMANHLIVSTRNVPGEMQGTQRQIRARK
KVAVTVLIFVLVFALCFLPYHTFMLFFYFNPNAQDDYNEFWHYLRIVGFCLRYLNSCANPIALYWV
SGAFRKHFNSVARRNTMYPVSELRRKRIFRRLHLKALCETCNLSALRIQSATLFIASLASERLTTI
NECAKIKAKFLYLLCIKPARPRCDTCHGNHATSMSLVSTRRNQSYCSRKSRATSVRKPDHHIGQET
SITLLDREFLGVGRAPPTYELTYKKRSKNGTGHRSQDTRASSKYQSRKHPLMLEPTPNSFNASSCL
KFQIPRNYIRLFSYVRRARLSRTKESTPFGRIPSDVDVPKSDKSV
>abi17146.t1_1
MECYNLTELLDRERRLPSTMNVLIDLIRKAENGRSSQDARQLSIELLQRFRQGGIVRSTNSANSQF
SLPFSPSGRETFKSRITLRTLIPGTAVNFPNETLNAIERCSMHAMLSASIDTTTRGDEGNVCNRLG
RYERRTARDVSDDVDVFNPHDLSEKGNLEEEAQPEGEAPTENRENLDLPIGEHDVETLPRNSARST
TSVSVSQCPVENGVVYTRWGAVQAGTVIAGIASGFEPQTIREQGYTIDSRFAATLSGDLAEAALFQ
AAEYDDVKNIGASGGWNSTQVPRWYFISRTTNMEMTDADIRGGLDGLILALNVQSWKTRFSTIKVS
QILDMYYSKRGVFSDTHRACNRNNLLTTVAPSSDLVAQTTAFGFHFDNAATFPGTIVDDAISTLSE
RAVELFYSYTATLNDLNCQIDSLTRDRTATDVIIVLDAQWPYATIYPALSYLLDNLDVNQYGSNYT
IINGNNGGFIVNTSNSIADFHESFNQSVHEAQPNGFQYTLIFNEVEQIVKNRMDDDQLKGNLGGKS
TVVLFVPYQITPSEADNNYAVERRIIHRSYLPDFKLLVLGYGSSDSYNSLVSNAASDIFTLSVSSD
GTSIVSAVGPVVSRIQEVQRRIVNPNCGSHFSGSSSSHSFDDFVEPETVNYYRVAPNYFYGEGDRT
LKVRGQGYGNIVVCTSTVTTHPRQNGTSGNANCKSITSNEESFSLTGLCNDGFISDCSPFYFSVES
PTVNNNARCSDLDVNAKTSTALPEILRDCYRKNVTDNLPQVPLNIQVLVELIRKLEYDIGNAINMR
ILTTSLLHGIRLDGIEKSSTGLESDFLIPYRASGHQFYKFKLIVDYLTFGDSLQFPIDAINQNDLC
ILHLITSNTLDIWQRGDETRTCVVRSMSKREVVNLASRQISECPIEKGVIKTRWGTISPSHLIAGV
ASVYQENSVKFQRLVEDFGEEKNITRTFNDKTKDEVLNNLLVATIIGDLAEVLLNQAFQTPIFDEP
GRWNDTSYPKAYYVDDVISDMTSAELLGGIDGLLIANKVKEWTSILDRTRFSQILDMYYSERGVGY
DRKYRACERRSKLKSLFEQYDFISQTSNTAKLLQEMSQYDRYLSEEGIDQYAESVVSHYKNAADAI
SSHYDACQQGGDNITNLEIITILDGTWQRYEAQSIISYLTEVTDISHYGSKLGVINGQTGKWMANL
SGDVIQIFENLTKTVSWPSRLQLSDSLNTVISHFQNKTKPDCNSRKLRPMGHVVLVLVNTALITEV
DAKNADLSIQSLKKSHPHVKIIYVASEYNAAHYEDFATNVYGNDLVIKTINDIATFVDRISETLSN
MPANIMNYYCNVSTVKLEDYITPRIDTLYEIHSDYIQRTDITIKFKGNNYGDLTICAYNVNIIQNR
ICKAVTVNDEVTFNVKDHCTVESSCNVQFSISTNTSQYKCSGNWYKLAVLTLTGGMNKRESHRGSK
MDYLVSLYGCMFLIGVLGNGTLGLALFSGPGAKHRSPLLLGLVAADFFVCCFSGPVTAALYTISSW
TKSWSCVALFVQAWPISASTLSMMTLSVDRYLTVKNYRPAGQVVRRRPLLLSVVAGTWLSAALFSS
LQFVQQNPLKRSFLIVRIIFVHLIPACTVIACHIGVHAKLTALSLTARAKHGELPLPMPLLRRPTH
VIIVAGISNRLESEGRNGLRKAESDPEDIAQPPTSTLRSRRRLANSLLWVAVIFAACWLPYVVCLI
LDESSGPPPEIVQRYCLLLGHVHSALSPLMYWTLNHQWLQRPCRFRLPALYRSASSTNEAALGPFH
PRLVRPPPVRRRSSHYLY

>abi17418.t1_1
MLYVYVRISCIVAQRHDQLSHISTRSRKSQKLVLNAKEESDIDRASSEYEDHNLKCTRYISHISAR
IAAQEPLNPNNYVETRKNGGHSRSLRSTLSYAYASEPVTPQRAASLYTATNLSTNRSRPESLQLDV
SILNFEPATRVSSFRRETKTAQTLSIVVGGFVVCWLPFFVYYLLTPVLPTNEANKVIMAYLTWLGW
INSAINPFIYAFYSPDFRIAFWRLTLKHFTKSRREDIAFQHK
>abi18263.t1_1
MNTFAFFVVTVTLSQIFAENPTLKERSTDSNIAKDVQQNIENLPSYLSNMNVTEPIAENVTLSNIT
SETNITQKLISCVPNFGGSDVQLVNDTELIKLLLPDTNVTSKETPANCLAVLFYSKYCPFSSMAAP
HFNALPRAFPDIKMIAINAMMYHLFNTQNGIVGVPSLLLFHNGRAVAKFNYSDYTLELFSKFITMY
TGIKPKEKSYVTSADFSGPVSSVPTKDTDVFLGLAWFFIIMCGVYCFTKSKWWKWIVETIQNINKT
TLSVRKSVVLKSFFKLECPKFTCANYDARSLGRRVGHFTQQTSVQCGHFNQFDSKSTSVDPIKIIK
STLQIDISNSSFINEIWVIAEPSQIIMKTSAFLPVVLWGIFGNICLLHIILKHRHLRSPTNLLIGN
MAAADLASLLIHPWVCLIYDFFQNYQLGEFGCKVEGAIECAILLTGVMSLSAISYDRLTAIVLPKE
TRLSRKGAKIVMGLTWVCGLLVASPLFAYRSYKVGNLNDKKNDIQERQWLDFLEKYCMENTMVINI
YWHVIITMMVWFPLIIMVVCYVGIFLKLNNYEKVVKKQRTIHVNYKAKVAKTMFIVVVTFMVCRLP
FTALIFYRNELLKNDSIAKTNVVQNQVNGIYTTLWFASKFLMFANAAVNPVIYGLTNEKFRRAFRD
TAISRCLFACGDEKPQKIPTKKKTKETMLGSNKSKIFVIFKGKNAKNVDAPPKRPC
>abi18590.t1_1
MNISNSSDATYYEKTDSGRLNLYYTVCEVLVAIFAVVGNAMVILVFRKERRLRRRTNYYIVSLAAA
DFLVGLLGIPFAIMASVGLPSNLYACLFTVSLLVVLCTISIFCLVAVSVDRYWAILHPMGYSRNVR
TKTALX
>abi18686.t1_1
MSFATVMNKKTKKNPLMDGVPYGRWVTRRVAVGTIATIWLLAALISFVPISLGLHRPSEPPPLYNA
GGQNYSTCALDLTPTYAVVSSCISFYVPCIVMIGIYCRLYCYAQKHVKNIRAITRPIDMPEGATSK
KKSFKQKNKALHIHLHNHHSSPYHVSDHKAAITVGIIMGVFLICWVPFFCINIVAAFCKTCIPDIT
FKILTWLGYSNSAFNPIIYSIFNTEFREAFKRILTTHYPVWCNCGYQSVALNTNDKFVTDYGTKTV
IVNTKRNGSFGEFSSEHLSTESVRHTRVNSTEKDILGEDISAI
>abi18927.t1_1
MRLGRIWSLSLAYADDLVLMLEKEEGRTEAECKKNEGNGVQEWWRKYERPDNSPGNIYTHNLNVNN
ADTSTFDHDRELNSISTFSAINFKNNYRYAIKVKYAQITLIIRTGNVAETCENAKLCPIEWRARRN
EIALEPPQFRTIRSEVKMNIEEDFDSSWAYDADYNFSSFNISEEKYIQEIVEYVTPNFWIWVSIFF
FGLVFVVGLIGNVLVCTAVYRNRGMRTVTNYFILNLSVADFLVILTCLPPTVVWDVTSTWFFGDVA
CKVVLYLQTVSVAVSVLTLTFISIERWYAICYPLKFKSTTARARKAILFIWLVAFVFVLINDGPKG
YRAQGSGQSFKV
>abi19126.t1_1
LRQIVTISPTHGTSTAGRSTSGGTMLRMLGAAQKREVKATQNLSIIVLFFMICWIPLYTINCVIAF
CDKCKINSTFMLMCIILSHANSAGNPLLYAYHLKDFREALKHFICDLFHYENSGAVTSSNAFTHYR
LHSLRESRTYNFSHQRKYIESPIYLSRTTKSMSLPTPPKLLSIVPNSIALAAATAVDKEMWRISEV
PSIMDNSNNDSSGMHTRPSTPFIKANSSSSSIKNCSYLNDINYDEDDDVFLDDTLPISDFNCEGME
FQNNIFDKLCEAPVITTFKQQHKNCLSSSSPQLSRNLFLIDTEPDFNYKSVRCNSTTDKSEFKKCP
TSSMSGEYIYSPSRNIKMSPLKMVGEYLWNASKYPKSASLSDDSNVSPKYKRKNNGSVSRDDWVVR
LKQNILTQIGSHVNSSIPKPDFKINITSLMTNVYNGRNTDDDPVHAEKIRSFYPICEVPKNTDQDL
WKDENEEALNLYFNVDYERNATIASATIRLHRIPSDNLTDPFATNERTCENSNEDEKLLRITIYWY
TKSLRKHRVKKRMCDSKVVTENVKWVELSVKSATKAWNKGRNLGLAVTVEDQDGTSLKASRYFKGA
SCTVGMPTPRPIPTIIVDAAKKANKLDRDGDPVGHDTIPIVHPDALLLPTLDICTWEFPSDLHDTR
YCLLQKIHEQQSNHRIRHQRRLEENSDRMESTRQAAAPDPRSRIVDKQIVITSENVARFNANINR
>abi19527.t1_1
MQTVTNLFITNLALSDILLCVLAVPFTPLYTFIDKWIFGKAICHLVPYAQATSVYVSTLTLTAIAI
DRFFVILHPLRQRMKLSTCFIILLGIWVFSMLVTLPYGICMQVIVHEGREVCEEKWPLDNLEVAFG
FSTLIMQFIVPFFIIAYCYLRISRRLNQRAKSKPGTKSARREEADREKKRRTNRMLIAMVTIFGVC
WFPLNLVNFINDIYDIGEFFHLIFFLTHAMAVSSTCYNPFLYAWLNENFRKEFKQVLPCVLRNSSS
RYCNGDEHTLDTLLHSAVIISPIKQLEPREPIAERIAPSQDNSLTDTVAVDTYSSACSLNLLWWRL
KPPT
>abi19583.t1_1
XEFQKEIKAYRGVTRVRYMRLFAFRIRNGRSIFRDDQTPERALQKAFTHVDGGNRIAMIHTRDTST
MIILSDSDDDDVIFTLESLTQAAIIFIMGVAIIVANEQRFLTMERAHWENVLKRTISVIKLLAAEN
MAFRRKWGVLYRNKGICIQLLAEYDDVIEAHEARIKNKELTNHYLGKHIQDEIIILFGQNIQRQIR
PEYVVVKINESFATFLNDSSGEGLFENIMDLFQKLELPITNIRGDNGANMRGKHSGPSDVINCYLL
SLAVADLLCGLLVIPLSVYPALVQRWVYGDIVCRLIGYVEVTLWAVTVYTFMWISVDRYLAVRKPL
RYETLQTKTRCQCWMAFTWISAAMMCCPPLLGFNQPLFDDESYICMLDWGNMAAYSVTLAILVLGP

SLITIIYTYTYIFNMMRKLRSGVPIHDKEYATALSENLSNPSHIMSFVLVMAFWISWTPFVGVRLY
EYVTGVKLPIPFLHFGIVWIGFMNSLWKSLILLFLSPQFRLALRIFCMTICCRYKGRMQAELIGME
ADD
>abi19946.t1_1
MIHEYLGAAFSPTSTGSILRPYILSLYKPSNNIFTKAWVFLAMSVLGNPRFPIWHARTGGGFGGNM
TVVDKVLPEMLHLVDDHWYQYPPMNPMWHGILGLFIGALGFVSFAGNGMVIYIFCSTKSLRTPSNL
YVVNLAFSDFLMMLSMSPTMAINCYYETWVLGPFMCDLYGLAGSLFGNTSIWTMTMISRDRYNVIV
KGLSGKPLTIKGAVLRILFVWVHAALWTIAPLFGWNRYVPEGNMTSCGTDYLTKDWLSKSYILAVA
AHEKAMREQAKKMNVASLRSSENANTSAECKLAKVALMTISLWFCAWTPYLIINFMGMFIPSKVTP
LTTIWSAVFAKANACYNPIVYGISHPKYRQVLHQKFPSLSCASAASDTTSVASGVTQATATDEKT
>abi20794.t1_1
MLLAEEDRAGCTRSFCCSEEVPQTSFSLRFCTLDCLKTSSFLMEYNVGGSGSVVEASALEHSLHRN
GYCSDWRQPHRYMDCACPQKDEDLNLSVADTMVSTLNVTFSYIYMLNSHWPFGKLYCKISQFISVL
SICASVFSLMAISIDRYMAIMTPLRPRMGKTMTLCVAFSIWVLGIVISSPCLLFFTLHSDGDRVIC
YLHWPDGPTNESMIEYIYNVAFLIVTYVVPIGSMTYTYARIGVELWGSQSIGECTQRQMENIKNKR
R
>abi21691.t1_1
XHFPLPSVYCLAWICLDVLFCTASIMHLCTISVDRYLSLRYPMKFGRNKSRRRVTLKIVFVWLLSI
AMSLPLSLMYSKDQDSVLIDGECQIPDPLYKFIGSIICFYIPLIVMLLTYALTVRLLAEQRQNLST
PDWSSGWLGGPTTTGLGKFNNTLLQTENN
>abi22199.t1_1
MVRHLLPIEVQVDNMTRKTCHWNDMVFGSYIPELIVYRTEKNTHLQIDTIYFTQCSPSWSADVDTS
WTILKMFTLYAVPLVFMSVTYCQIVRVLWKSGKVTRDTFATVEGSSTIAFTNYNTNMECQLSSRKK
AAKMLVVIVGMFAVCFFPVYLLSVLRLTVGLTNSDANRAFALVSHWLCYANSACNPLIYNFMSRKF
RKEFRRAFDCCSTSDDGAEFQLNSLRKRHAAKNCGTFRRTSSKNTSRSSRMTNFDSYNFIHRKSGK
SNDVKRDVM
>hom556.t1_1
MDPFVNGTVSPDGEDEYTPYDERPETYIVPVLFLIIFVVGVLGNGTLVVIFLRHRAMRNVPNTYIL
SLALADLLLILTCVPFTSTIYTVENWPWGVALCKMSEIVKDISTGVSVFTLTALSAERYCAIVNPL
RRLQTKPLTVLSAVLIWLLAMILSIPDGVFTNLNEFAVSNATRFILACTPFPDNETSKLYMQYNVA
GKALIFYVLPLCIIASFYILMAKKLHASANEMPGELQGQGVAQVRARRHVARMVLCFVFLFFICFF
PYHLFALWYFLNPNMEAEYNDFWHALKIIGFCLSFMNSCVNPVALYCVSGVFRQHFNRYLFCDNTR
RLRRNIPSSMGNCETSFNSTYRRNTQGTMIESHCTQRKHAPFGRQSTQDTTLIPMDIANNKETKDL
NAKTTMILHNEKKKDAGSLGS
>hom580.t1_1
MGDRGILYDSTTSSSDESVSFATDESFFNAMTENFTYVNVTSQQKTQLMLYDVLIPTIGTLSIVMN
LAVVISSGLILKKGQQPRSTYLFLGNVAMTDLITSIAIVFGQTFPKNLRDRHICAIQMGCTIGFLP
LMGWAGDTQNGKICWFIILAPKELILLTVSIGALPLILVVVLYSIILYHAIKKIIQLQKAHEADVH
NKVETDSKGLRIFRGRGAQSDTSDVEDHSATPREEKSLFKRIFTRKPQNNLNNPSKWKAIKVVLFT
SGSFVATWSPYFIACVVYIYCGDPQTQKCKTLKILIASPLAILGFVNSLVNPIIYAWWHKGFRSFV
TQKFNSIRKKKISISNTSSTDTKKTSNASNSDQKKSIRSMPLSNSTEHIIDENGSKTDSSSKSETT
GETKLSNTTLDNDEREKSDKKFIVSKSDYAGVHNSRENIVKVEENGSTDL
>hom778.t1_1
MSALGQFGAEMLKLSRGLEAASTNSAKSRNCQLNSHESTLQIGSKESNSSSIFDSNSPLLTTESSM
SPWSMKRRRASTFHEGDTRDESPSPRWRKRRTSFHERRPTDMSNESKSEGTECGTLPLLSPYVCSY
FNEKSENSSPSRSSEVKIIQNEQRSSKLSLDMEEPIPTISPIKRTQLNKNRNNDQSANNIVVTWEC
PRRNHRRGSSFGSTRTQVLNSNQRTPLLLRRSATLRHNGHGATGLQDKNKNPSSPCLLQRYNYNNT
STTRSNAIRSHHSRNSSVISRNSSRHGRIIRLEQKATKVLGVVFFTFVILWAPFFVLNLLPIVCEH
CEKNIGSWVFQFVTWLGYASSMVNPIFYTIFNKVFRQAFKKVLLCRYRNQTWRPHR
>hom814.t1_1
MKKKLGIIRHTLQARQALVTHNSLTDDGFLAIWWPLKCQITKRRARMMIMFIWFVALTTTLPWAIF
FDLVVIFSDAPDVRLCIEVWPDSLNGALYFLIANMVFCYILPMILITMCYVLIWIKVWKRDIPTDT
KDAQMERMQQKSKVKVVKMLVAVVILFVLSWLPLYIIFARIKLGGNIESWEEEILPIATPIAQWLG
ASNSCINPILYAFFNKKFRRGFMGIIKSRRCCGRLRYYETVALMSSSTSMRKSSHFYNNNSSSRRI
PPVQDNAVSYIYNNTGV
>hom814.t2_1
MQSRPCPIFLAIWWPLKCQITKRRARMMIMFIWFVALTTTLPWAIFFDLVVIFSDAPDVRLCIEVW
PDSLNGALYFLIANMVFCYILPMILITMCYVLIWIKVWKRDIPTDTKDAQMERMQQKSKVKVVKML
VAVVILFVLSWLPLYIIFARIKLGGNIESWEEEILPIATPIAQWLGASNSCINPILYAFFNKKFRR
GFMGIIKSRRCCGRLRYYETVALMSSSTSMRKSSHFYNNNSSSRRIPPVQDNAVSYIYNNTGV

```
>hom1729.t1_1
MRVKGVSTHARRRERLIQCAVVERGGNMPSKLRVDSGAEDDEDDAVMALANVTARSAVGVEDILIM
LDNGTSNGTFSNGTVTITTSYPSGYTLPQIVLASIIVTILMIVVVIGNMLVIIAITTEKALKNIQN
WFIASLAVADFFLGLVIMPFSLANELMGYWIFGAWWCDVHSAMDVLLSTASIMNLCLISLDRYWSI
TQAVEYLKKRTPMRAVVMIGAVWLLSALICIPPLLGWKVARTPDEQFPKCQ
>hom3063.t1_1
MQKEISAGLNTTNDTTIHIEELALAGLLSIVIVITVIGNTLVILAVLTTRRLRTVTNCFVMSVAIA
DWLVGIFVMPPAVAYRLMGKWELGWILCDIWVSLDILLCTASILSLCAISVDRYLAVTQPLSYSRK
RRSKRLAFSMILVVWIISALITCPPMFGW
>hom3345.t1_1
MIFSLCRYWAVTNIDYIHHRTAKRIGIMILIVWCVAFLVCIAPLLGWKDPGWNVRIEDRKCIVSQD
IGYQIFATSSSFYLPLMVILILYWRIFQTARKRIRRRQILSNVAGQGNKNPAAGGIAGGLAVASGT
GGIAAAVVTIIGRPLPTISETTTAFTNVSSNNTSPEKTSFANGLEADPPTTTDGAISCYQPQYPVK
RKSKDSADSKRERKAAKTLAIITGAFSDVLSMENPVAARLAASVAATAVASGPRNVTFKCD
>hom6288.t1_1
MSTLPFQVVLFVIVCLVGLLGNTLVIYVVIRFSKMQTVTNMYIVNLAIADECFLIGIPFLIVTMIR
RSWIFGEIMCKAYMTMTSVNQFTSSTFLFVMSADRYIAVCHPISSPKWRTPLISRIVSFAAWTLSI
TLMTPIIINSHLVESNSENSTSGSCVITFSNETYHQTARAFTLYAFVFGFGTPLCFILIFYCLVIL
KLKTVGPQNKSKEKRRSHRKVTNLVLTVVTVYVICWLPYWITQLATTYTSDEVIEIDINFVRPPVF
QQSIDVVFRRRFCRRIWMWKQFLALIGDAFDNTTLNMWTSIIMVENDAVPFAWSFRLDCSGRVHRH
HC
>hom6540.t1_1
METTKRYETETVFSNETIVWSVVDLTLLLITVAGNALTLCAIILSKKVSGLLSNRFVFSLALSDLM
VGLTIPYHFAFSVGHTLSENRETCILRFVLVIFACSSSIYNLLAIATDRYLAIVHPLRYGRYMQKR
IAYAVIIFGWCVTLFVATVVIYWNRWAKGKECSLNHVVPAEYVTYVLIPMFISVWFVMLLIYIKIW
RVANKHAKRLRDVTKSHNGTMLPKDWKSVKVVMLILGCFSICWMPYFATIAYIRIFGHIPTLLYEI
TFTLAMANSSMNPVIYAWKNSNFRKSFWCMLRCKSPNSLVEKPQYVTNHVPTIDRNVARQEHDENN
VTIYSKLTFEIDDETNDDRKSDTTSADTISR
>hom6621.t1_1
MDKNETNSTDDENEYKKSLYKYYVPLLVILCLVSIVINIKILLAVHWLRRPISPTVHISLSLAAAD
AFSSTFVGVGLIINNYLSPVFDVNLPCYIPIMAEIYRLSGIIITVVHLLALSMNHWLGIMKPLHYS
SIMTTRKITITVTFLWILPIAFFNIYFLVASEEDFWQRGCTNRDTFLHQFRFRIIFSLLFFIPLFL
MTFFYSHILLIVKKQQKVWANLSRSGSKRTKNLAKANSQQRRNLEGNIKAIFTTLLILGSCMIGWM
PAILKFILICTDKCIYSIDDTKNISITMNFFISYTTHVLLILKTLANPIIYSARMLEIK
>hom9099.t1_1
MNEIVIIPDLVTNLEENLTKDLENFSDKTEFAHPNKSIYTYSTNVDIGNNFASRTWILWLLVILKT
TVMALIIVAALFGNLLVIVSVMRHRKLRVITNYFVVSLALADMLVAIWAMCFNFSVEITGGIWLFG
YFMCDVWNSLDVYFSTASILHLCCISVDRYYAIVQPLDYPLIMTNVKLAIMLGVVWCSPALVSFLP
IFMEWYTTEENLIFRKKHPHVCNFEVNKTYSVISSSVSFWVPGMVMIFMYYRIYVEADRQERMLYS
KRKRKGNGTSGRTGKSPRIPNGKKDFTSGFC
>hom10184.t1_1
XSKVAAALLNKHLQINGISAGLTTLRESVDADFAEAKAEAGSSSKMKRERKAARTLGIIVSAFLAC
WLPFFLWYVITSLCGSELCYNPPAVVTLVFWIGYFNSALNPLIYAYFNREFRVAFKKTLQNCCRFT
SKIMCWKCRSSRDQITYSNASSEIHMNNHLRISDQRGEGNQRLSYNISEGEIINLQSEAVI
>hom10848.t1_1
MDVNKGLGHVHLYLMLSPLALRLPNNPVELATLKKNSTMFSSRLFNKSSILYFVMLYKLVNNLQYF
SYYRFRTNTINKNEKIKENQNKFEQRVSMASPGSVDAFPKIRRKRRNRVDRRQTQNVENMIVNAMA
SFSATFTTNPPILLHNANNLQNRTNITNIENLHPDWTDLLLLVVKSCIFGTIILSAVLGNALVIIS
VHRHRKLRVITNYYVVSLAMADMLVALCAMTFNASVELTGGKWLFGYFMCDVWNSLDVYFSTASIL
HLCCISVDRYYAIVRPLEYPITMTTKTVSFMLANVWILPALISFTPIFLGWYTTEDHKVYRSENPN
VCIFVVNKYYAIISSSISFWIPGIVMITMYCRIYKEAIRQRKALSRTSSNIILNSIHQHRTSTYNT
RYGDHYLHPSDGELTTIGQINGRRSTSSGSAISYGTTTNMDNCKAATELNMNGKFYNW
>hom11489.t1_1
LGNRFDEQRLTLRIHRGRGSSGRRHHGSPHSNGSNSTITTESASPSPRGKHERVKISVSYPSSDNL
SPHPAPHLLAITPTSPTGQPTYTVHYTVNGKENTSTNLCRRDKHLRVSQRLAAHRRNSCDSNEDRM
QRSPSPSVCDDGINKPKIISKRMGKRNIKAQVKRFRMETKAAKTLGIIVGGFIFCWLPFFTMYLIR
AFCVDCIHPLLFSILFWLGYCNSAINPLIYALFSKDFRFAFKRIICKCFCKRHCSSASRRGSDGSQ
LQGRQFRSPSFNIQQPGNSLGEDSDPGGDPSDSX
>hom11930.t1_1
```

XSVDVKDGNNTSLDIIDDTVVQALFLMLYTSIFILGIFGNVLVCYVVFRNKAMQTVTNLFITNLAL
SDILLCVLAVPFTPLYTFLGKWIFGSVICHLVSYAQGASVYISTLTLTSIAIDRFFVIIYPFHPRM
KLSTCIFIIINIWLFSILVTLPYGIYMLHTHDNKTSEAKYFCEENWPSEKYRRIFGGLTTLMQFVI
PFFLIAFCYICVSLRLNDRAKSKPGSKNSRKEEADRERKRRTNRMLIAMVVIFLLSWLPLNTINVI
NDFSIHLGKWQYYLLSFFMVHAMAMSSTCYNPFLYAWLNENFRKEFKQVLPCFERAARTTASGRIG
NWRTERTCNGNNETQQESLLPSGIHRATSVRERKTTPSQPTKTDSVEVENVLVPTVGAVYDSVAET
VRLKLISEEDPPPYEATIKNDL
>hom14903.t1_1
MEQNDEDFMMDPMEQYNTSYNASVTNSTTRYTPYVERPETYFVPVLFFMIFVVGVLGNGTLVVIFL
RHRTMRNVPNTYILSLALGDLLVLITSVPFTSVVYTVESWPWGELICKLSETAQDISIGVSVFTLT
ALSADRFFAIVDPLKKFHTSGSGRKATRITLGIAISIWISAIICAIPSAVGSHLVVKGYDSFQFTI
CYPFPETWFNKKYPKINVMTKFLILYVVPLTIIAIFYMNMANHLIVSTRNVPGEMQGTQRQIRARK
KVAVTVLIFVLVFALCFLPYHTFMLFFYFNPNAQDDYNEFWHYLRIVGFCLRYLNSCANPIALYWV
SGAFRKHFNSVARRNTMYPVSELRRKRIFRRLHLKALCETCNLSALRIQSATLFIASLASERLTTI
NECAKIKAKFLYLLCIKPARPRCDTCHGNHATSMSLVSTRRNQSYCSRKSRATSVRKPDHHIGQET
SITLLDREFLGVGRAPPTYELTYKKRSKNGTGHRSQDTRGHVQRLVSLFHAEMI
>hom15180.t1_1
MFLIGVLGNGTLGLALFSGPGAKHRSPLLLGLVAADFFVCCFSGPVTAALYTISSWTKSWSCVALF
VQAWPISASTLSMMTLSVDRYLTVKNYRPAGQVVRRRPLLLSVVAGTWLSAALFSSLQFVQQNPLK
RSFLIVRIIFVHLIPACTVIACHIGVHAKLTALSLTARAKHGELPLPMPLLRRPTHVIIVAGISNR
LESEGRNGLRKAESDPEDIAQPPTSTLRSRRRLANSLLWVAVIFAACWLPYVVCLILDESSGPPPE
IVQRYCLLLGHVHSALSPLMYWTLNHQWLQRPCRFRLPALYRSASSTNEAALGPFHPRLVRPPPVR
RRSSHYLY
>hom16632.t1_1
MPEGATSKKKSFKQKNKALHIHLHNHHSSPYHVSDHKAAITVGIIMAAFCKTCIPDITFKILTWLG
YSNSAFNPIIYSIFNTEFREAFKRILTTHYPVWCNCGYQSVALNTNDKFVTDYGTKTVIVNTKRNG
SFGEFSSEHLSTESVRHTRVNSTEKDILGEDISAI
>hom16842.t1_1
MRLGRIWSLSLAYADDLVLMLEKEEGNNYRYAIKVKYAQITLIIRTGNVAETCENAKLCPIEWRAR
RNEIALEPPQFRTIRSEVKMNIEEDFDSSWAYDADYNFSSFNISEEKYIQEIVEYVTPNFWIWVSI
FFFGLVFVVGLIGNVLVCTAVYRNRGMRTVTNYFILNLSVADFLVILTCLPPTVVWDVTSTWFFGD
VACKVVLYLQTVSVAVSVLTLTFISIERWYAICYPLKFKSTTARARKAILFIWLVAFVFGKYLALL
SLTNPFCFI
>hom17707.t1_1
MSVLGNPRFPIWHARTGGGFGGNMTVVDKVLPEMLHLVDDHWYQYPPMNPMWHGILGLFIGALGFV
SFAGNGMVIYIFCSTKSLRTPSNLYVVNLAFSDFLMMLSMSPTMAINCYYETWVLGPFMCDLYGLA
GSLFGNTSIWTMTMISRDRYNVIVKGLSGKPLTIKGAVLRILFVWVHAALWTIAPLFGWNRYVPEG
NMTSCGTDYLTKDWLSKSYILAVAAHEKAMREQAKKMNVASLRSSENANTSAECKLAKVALMTISL
WFCAWTPYLIINFMGMFIPSKVTPLTTIWSAVFAKANACYNPIVYGISHPKYRQVLHQKFPSLSCA
SAASDTTSVASGVTQATATDEKT
>hom18184.t1_1
XLCISDLLFCFLVLPFGASQFFHGKWIHGDFLCILIPLLRYGSVGVSLLSVATISINRYILIAWPQ
LYPKVYTKTKVAVFIAMIWIFSYGLMIPTLTGVWGEFGYDAKLGTCSINKDKNGHSPKVALFALGF
ALPCIIIIVCYAKIYWVVSKSNKRMQQHVSSSSKRGEMHVTKMVLAIFFCFVICYLPLTLVKMFDE
NVSNPPLHVLGYLLLYLAACINPVIYVTMNKQYRRAYFNTLTCKFSNNIDSNTPVQNSKSMSVMFA
KNIINNPKV

Insulin receptor substrate

>abi3552.t1_1
MSTNSSSREELGPKSRTRSSSATESSKPTNVGRRQNQPNPICGVVKPIPHIQDTTFQNAAEVQLHA
PPYQPSVALMISTATVKLKINDWYAMNATCTNDFRWVIWVVCFIAAGGAAWSGTINHQRTRSLPLA
HPAPSNDHISHPPRAAKRTTNQSSKCMYHLADDRRTGLFGAFRTDADRRLRNHRIDNAELAVFCFF
YVSRFSGSGGGRERCDSMPSRARTTSEGNHFISIFNRPHFRPPSLYKESPSGSPVSPPSAAGSTDS
AGSSLSIDEPDNTWTDTNDVLRYSHSLTPDEAIAEENFEDCPDGNFIMRLVGNNSYIPMNLGTTDD
GYVKMQSRNQHMSPTASLSSVTSGTPSTDMKFAEYTLEKVPSYFTPSEDDETSSTDRPTRAYSFGS
HPDSYKYRSMQADAARERAYSFGAKTKKMQRIFQHPQANSGSKSSSAPLLSNSRVNSHGSSDPMND
LMEIDFSKSKENKTNRVPPGYVEMKPGSLENNRINRNGGGGEDVTGTSGYVDMKCGRSPGKSSTIY
YVKRNSPPKSTGYMEMDPRKKMATSPLSPSFLPGTSDYMDMNVAKKTPRVQLTSFARYSGHHDDST
PSSIEFIDEDEPKPKSHGSDGYVEMNVGKSTSSHQRQSSLDSAKINVGDYTNMSCGIPSSTMTSVT
PSSSSSSSKKRERKLGNRSQPIQIQGNGSSQFPKSSSPSNPLLYGRKYSTGTPPKMFLNLNTSSLP
RQKSKKVSVRRDSKDSSSSSVTTPSSSSTIFPISINSPCSPVKPPVEASDAYTSMDFTRSSPKNGN
EYVNYNPAKTRQQESGGDYALMKPSNSSVLVVPLSTMNLVESKCFRPIPETNESRTTSPRQSIDVG
KSPESPKNSRSNSINGDGAGSEVTLPSVSRPSSTSSELNSVTSTIVGSRPSSVNSDLVRPPSVSSE
LCYASLDLEDGGSKSPRTVKPQTQTSSEGVAPSQPDQDPSFTYAKIDFVKSEGFRHNPLLPNNAKV
KH
>abi11141.t1_1
MSSRNHHDQQVIKSGYLKKLKTSRKKFFVLRGETAKTPARLEYYDSEKKFLGGHNPKRSIPLKTCF
NINKNQFDAKHRHVVALYTKDDVFSVAFDGEEDLEAWYKALLALQRGEDVIDGKVPKPTFGDNVAS
YVRPENLFTATPPRRYKKVLIDTGFPINVHIFF
>hom3167.t1_1
MSTNSSSREELGPKSRTRSSSATESSKPTNVGRRQNQPNPICGVVKPIPHIQVALMISTATVKLKI
NDWYAMNATCTNDFRWVIWVVCFIAAGGAAWSGTINHQRTRSLPLAHPAPSNDHISHPPRAAKRTT
NQSSKCMYHLADDRRTGLFGAFRTDADRRLRNHRIDNAELAVFCFFYVSRFSGSGGGRERCDSMPS
RARTTSEGNHFISIFNRPHFRPPSLYKESPSGSPVSPPSAAGSTDSAGSSLSIDEPDNTWTDTNDV
LRYSHSLTPDEAIAEENFEDCPDGNFIMRLVGNNSYIPMNLGTTDDGYVKMQSRNQHMSPTASLSS
VTSGTPSTDMKFAEYTLEKVPSYFTPSEDDETSSTDRPTRAYSFGSHPDSYKYRSMQADAARERAY
SFGAKTKKMQRIFQHPQANSGSKSSSAPLLSNSRVNSHGSSDPMNDLMEIDFSKSKENKTNRVPPG
YVEMKPGSLENNRINRNGGGGEDVTGTSGYVDMKCGRSPGKSSTIYYVKRNSPPKSTGYMEMDPRK
KMATSPLSPSFLPGTSDYMDMNVAKKTPRVQLTSFARYSGHHDDSTPSSIEFIDEDEPKPKSHGSD
GYVEMNVGKSTSSHQRQSSLDSAKINVGDYTNMSCGIPSSTMTSVTPSSSSSSSKKRERKLGNRSQ
PIQIQGNGSSQFPKSSSPSNPLLYGRKYSTGTPPKMFLNLNTSSLPRQKSKKVSVRRDSKDSSSSS
VTTPSSSSTIFPISINSPCSPVKPPVEASDAYTSMDFTRSSPKNGNEYVNYNPAKTRQQESGGDYA
LMKPSNSSVLVVPLSTMNLVESKCFRPIPETNESRTTSPRQSIDVGKSPESPKNSRSNSINGDGAG
SEVTLPSVSRPSSTSSELNSVTSTIVGSRPSSVNSDLVRPPSVSSELCYASLDLEDGGSKSPRTVK
PQTQTSSEGVAPSQPDQDPSFTYAKIDFVKSEGFRHNPLLPNNAKVKH

Krüppel homolog

```
>abi17457.t1_1
MDLPCPVCIHNLWGQKPTEECPRSYAMHTHWAEPILRRNVLMHQLWSSKVQEMVGFYGEANLPVGS
VQTSEEARLLSVKRVVCSPDLPITGFPGQPHFDQQNVQCQLCNKMFASKTAFQTHQRAHAKESDDP
YRCDICGKTFAVPARLTRHYRTHTGEKPFRCEFCNKSFSVKENLSVHRRIHTKERPYKCDVCSRAF
EHSGKLHRHMRIHTGERPHKCSVCAKTFIQSGQLVIHMRTHTGEKPYVCTVCNKGFTCSKQLKVHS
RTHTGEKPYSCEICGKSFGYNHVLKLHQVAHYGEKVYKCTICNHTFNSKKCMEAHIKSHSENAPPA
TPPSSSSSSESSCSSVSDKENKDIPMNLVPQNQEIPTMSYDTDIRYYLYSRERLPSPLPSYLHPSN
TTSGVDLLAAAATATERYDQPEAVLNLISRSPHEIVTTLRHPSYFTAPSLQYDSPFASGDDIRRRV
EAALAVEDVPSEDEPILTPPSSNPVSPEPPSPDPELSLPPRKRSKMILKSMESAKDLSPVRHNSVI
HYARAS
>hom15480.t1_1
MDLPCPVCIHNLWGQKPTEECPRSYAMHTHWAEPILRRNVLMLVIMSRDVKIGEMQEKLISLMMTC
KSSESDDQLWSSKVQEMVGFYGEANLPVGSVQTSEEARLLSVKRVVCSPDLPITGFPGQPHFDQQN
VQCQLCNKMFASKTAFQTHQRAHAKESDDPYRCDICGKTFAVPARLTRHYRTHTGEKPFRCEFCNK
SFSVKENLSVHRRIHTKERPYKCDVCSRAFEHSGKLHRHMRIHTGERPHKCSVCAKTFIQSGQLVI
HMRTHTGEKPYVCTVCNKGFTCSKQLKVHSRTHTGEKPYSCEICGKSFGYNHVLKLHQVAHYGEKV
YKCTICNHTFNSKKCMEAHIKSHSENAPPATPPSSSSSSESSCSSVSDKENKDIPMNLVPQNQEIP
TMSYDTDIRYYLYSRERLPSPLPSYLHPSNTTSGVDLLAAAATATERYDQPEAVLNLISRSPHEIV
TTLRHPSYFTAPSLQYDSPFASGDDIRRRVEAALAVEDVPSEDEPILTPPSSNPVSPEPPSPDPEL
SLPPRKRSKMILKSMESAKDLSPVRHNSVIHYARASHVLEVGLPEYGIPSLETVTIEEFNLDLETL
GIAGASGYVNVNDVVLSGLTGFYASALKGTITIIPLGYTIDGTLTIPHISFNTGYDMDLIADNIIV
YGTGSASMDLYDITIDIKTSIGITGGINLRELSIFINIGSANTQVTGWFNDEEHSKVVSDLVNDFL
IPAWVNQQPEAISDLISPVLMEIINYYLANGGGDGAEETSIIDDILEMCAAEES
```

Target of rapamycin

>abi15881.t1_1
LKEGEPNPGVVLSVLLTIGDLAGVTGGGVELQQWMKELMTILLEMFGDASAPEKRGAALCTLGQLV
GATGHVIKPYTEYPILLDVLINFLKTEQHPFIRRETIRVLGLLGALDPYRHKINRGQIDYQPEAPV
LIAITDKSDDSNADLTSSEMLVNMSSSTLEEYYLAIAISTLMKIIRDPTLLQHHTMVVQAVTFIFK
SLGIKCVTYISQVLPSLLNVVRTADFNFREFLFQQLAQLIEIVKQHIRNYLDDICALIKEFWTPNS
SIQVTLVILIEHIAVALGAEFKVYLPKLMPQILRVLNHDTSKDRIVTIKLLEALRKFGNNLDDYMH
LILPPIVKLFNARVPLPVSKQALETIDLLADILDFSDFISRIIHPLVRTLDTNPELRATAMETLVS
LVLQLGRKFNIFVPLVHKVMTKHKIQHSKYEMLITKLNTETTLADDSDFKVPRNRIKNKNRDSNLP
TDSSILRLKVSSSNLQQAWTITRRVSKDDWLEWLRRLSIELLKQSPIPALRSCLSLAQTYSQLPRD
LFNAAFISCWNELSEPMQLELIKSLEQALIVADISEITQTILNLAEFMEHCDKGPLPLEQKILGER
AMHCRAYAKALHYKEEEFQRDANSQVVEALISINNKLQQKEAAEGLLQYVKQRNCDMQIQVRWYEK
LHNWDKALGLYEERLAVESGDQEACLGQMRCLEALGEWDALHKVVEDRFDTLSDANQLKAGRLAAA
SAWGIHHWESMERYVNVMPHESQDGSFYRAILAIHNEQYEEAQKFIDMARELLDTELTAMAGESYQ
RAYGAMVIVQMLAELEEVIQYKLVPERRHTLRAMWWQRLQAGQRKAEDWQKIIQVHSLVLSPHEDI
HTWLKYASLCRKSGSLMLSHKTLVMLLGYNPDDHPGECLPFNQPQVTFAYTKHLWMAGRKEYAYEQ
LQHFLDEYLQLFNSEDVTHDERRRLLARCYLKLGSWMVSLHGTTESSIPTVLQRFQHATEHDPHWY
KAWHSWAYMNFEAVLFYKTQSESLARTNRQVQKSDKPSDYIPYTVLAVQGFFKSINLSKGSSLQDT
LRLLTLWFEYGQWPEVHDAIVEGLRLIEKNTWLQVIPQLIARIDTPRSLVFRLIHLLLVDIGKTHP
QALIYPLTVASKSQSLSRRQAANKILKSMSEHWPTLVNQALMASEELIRVAILWHEMWHEGLEEAS
SSPRGHPPARLAVLFFGEGDVEGMFKVLDPLHARLERGPQTLKETSFHQAYGRDLNEAQESCQRYK
MTKIKQHLTHAWDLYYHVFRRITRQLPQLTSLELQYVSPNLLICKDLELAIPGSYSPGQPIVRIAY
IHPSLEVITSKQRPRKLLIRGSNGKDYMFLLKGHEDLRQDERVMQIFGLVNTLLLKDPDTVRCNLT
IQRYAVIPLSTNSGLIGWVPHCDTLHTLIRDYREKKKILLNIEHRIMLRMAPDYDHLTTIQKVEVF
EQALEHTHGDDLARLLWLKSPSSEVWFDRRTNYTRSLAVMSMVGYILGLGDRHPSNLMLDRLSGKI
LHIDFGDCFEVAMTREKFPEKIPFRLTRMLINAMEVTGIEGTYRRTCESVMSVLHRNKDSVMAVLE
AFVYDPLLNWRLVDTVGRVNRSNANDVGSMSTGSSQDQDVDSLSNNVSKKIIPSPETVGDGSEPEA
VKNKKAVKIINRVRDKLTGNDFNVEETLTIERQVDLLIQQATDNENLCQCYIGWCPFW

Odorant binding protein

>abi3665.t1_1
MALTDEQKEKVKQHYKECVAETGVAEELVQKGRKGEFADDPKLKQFVFCFFKKVGFQNEQGDLQLD
VIRAKIPTDVNKNEAEDVIKACKDITGTDAADKAFSVYKCYWNSTPNHIALV
>abi4098.t1_1
MQGNDQILAGELEELEEENNLYPTNPRHRRDGENSVKEEKCRRKDVKSCCEEEFLQSLKDADRVLK
KECYREITGKNHSSHLADPFRCENIEEHKRDITVDDKDNFKIEEMTKYLNEKLSSHEWLKQQMDVI
INKCVTEANETDAKRENDDKGACSPAAIKLGHCFWREIQIACPTNLIKDERKCNKIREEIKKKDNL
GFDV
>abi4505.t1_1
MKYTVLLIVFLATLFQVLSSPHNHYDYRSVCITELNLNISAFPEHADKEYIESDHDAQCLLRCVLQ
KKGEINSEDEFQEDAIKNKLPSEVKIDFDKCADIKDDDACETVFKRISCIDEQIPRPSLPPQN
>abi5092.t1_1
MIKYIVFFIAFSAAAVVRENVIEQIVTNKEECIKELDYDSPNTANLIQAHLVSLIEGNIKLGEDLN
YDGKCMLRCMMAKGDVINNDNQLLTEKLMTVFPDTDFEECKSIENDDACETAYEIHKCIVKALT
>abi6367.t1_1
MKYTVLLIVFLATVFQVLSSPHYYYDYRSVCITELNLNVSAFPRHPDKEYIGSNHDAQCLLRCELQ
KTGEINSEDELQEDAIKNKLPSEVEIDFDKCADIKDDDPCETVFKRISCIDEQIPRPSLPPQN
>abi7033.t1_1
MEELGRQLHTECQEQTGVAEELIKTIQDKKGFPDDNKLKCYIKCLMAEMAVIDDDGVIDVEAAVAV
IPEEMKDNVEPTMRKCGVKIGSDPCDSAYLTHKCYYESNPEVSLKIA
>abi7666.t1_1
MQTLLSTLVVFVALFTVTIGYNFDDAVYNQILANNFDDFAPDAQNFISNHRFRRDAEHKCHGRHSS
CCNEDVFQKMHEDGKETFKECFKTVTGKEFGEGPPKDPFSCENMEKRKKEIIVDDDGNVKPDDAKQ
FLKEKFADIEWIQSHIDEYVDKCATEAKEEAAKFDASDKDACNPSGLKFKHCLWKSVQLGCPADQI
TDENKCKKIREYIDKHGDVFPPPPLPHIH
>abi7667.t1_1
MIYHLNILFCHFADEILTSELQELLSGNVYGNVRVRRDDEAAKCQSKHPLSGLKSCCSNDIVDHYH
EADRPIMLECRNELYGKENQSDVPRECGNVHANKDLYLCLGQCVGQKLGYIDSNGKLLPDKLRNSI
KEKLSKIQWLQPELDTVINKCIGEVESANKESSEEGKCKPGASTIGHCVWREIQFACPAEEITDVE
NCEKIRNYLNTKPQIP
>abi7668.t1_1
MRIFIICVLLGVNRAYDFEGDYHNQNLNKNGNHPFTRIRRDDLADECQEEIKSCCNEEIFKRVHDA
DYEIHKECYKLMISNDTEVEPLNIYACKHPLTQKEITCVTQCVVQKKGLIDSKGNLIVEEFLARIP
EIYSDVEWLMPKIEDFFQKCNAQLNEFLEDHDTNDGCNPSAIKMQSCFWKEIQMNCPADEIQDEEK
CTKIRENIMNDATE
>abi8416.t1_1
XETRNASFTSQDSSNEDDKNISQLTTTGEISSDCVIHCVLSHFDMIDESDYPDRNKITQGLLGNAD
GRELKDFLRDSADECYQILEQDDRIGSCSYSTELVKCLASKGKSNCSDWPSDDLPVAQYF
>abi11926.t1_1
FTQEQINGIMDILYKFMNECAPETGATDEDIKDLINMRLTRREGKCLAACCNKKYGIQDGDGKINK
IKSLEVLKPMQKMDQNMYEKFYKIFDICAGAEIKLDDECDIAYEFIECYREHKIKFGMRDDVIPM
>abi15452.t1_1
MKQRANIEQKNNEEQEIGFLAVLCVTTASKADQQRAKEIVAKLLNECKTKVGATDADIQAIRDKQI
PKTKTGMCLIDCIFDKTHITKDGMLDKEGTIQAFTTVLQGNKMKLKTLSDLLDVCVKETSSNNMDR
CTNAAKIVECSSKHGKVFGLDV
>abi17067.t1_1
MTTKNVLQPLDVNSRNVDRSPVRKRARYESPIKERWSLARSDVLLDEVYDYGVSLLADSDSEAASE
DEKESCYNASKEEVAILESSLSKDTLKAGEWSQFLTKKKVEREVSTTDHFEELSDEMVLHILHFLP
KYSLSVVGLVCRRWHSLTQDESLWARMDVSCRQLKPGSLGHILSRQVMILRLAQSEISFPPIIEGC
RAFNEDFRSRLLFLDLSMAHISCDGLIQLFRKCRRLKKISLEHVAINTNVLETLAVSEDLEVINLT
MAEGLNEHGLKSLLTKCKKIRELNLAWTYMNTSCIKYICSNLPRTLDRLNMAGCRKLLNDENVREL
VNCCGGLRELDLSDCTGLTGDAVKHVTLLEGLNFLALSRCYQIPYKALMYLKRMTSLMYLDVHGGY
IETGELRLIQEALGPLVHLNKFKFSSIARPTVGTRRSSIWNMRPEKSRIIIPYRQYSAVPPKKEIP
GKGKGPISWQNVTITGVIGGGLLAFMFHLKNKKKQVLLRLRDVLALTQFNYEHHITCVLYIFSFTA
QIKERQRMVGKAAIGGYFELVDAENNLRKSDEFLGQWLLIYFGFTHCPDICPDELEKLSAVVDNLD
KSENVPKVQPLFISVDPHRDSPEIVGRYCKEFSTRLLGLTGTEEQVAKACKAYRVYFSAGPKDKDK
DYIVDHTIIMYLLNEAQMKAAAKLIRNTCRQKTKATDEQIDNMHKGIWDEDQNAKMKKNGAFDLEL
ANKQLPQLPENRKEPTVNSMNKCKDAVCVHVAQSALTEAQIASTSKLVRRTCKSKNKVDDIVLNAM

```
VKGNFENTDKNTKVTKDGHMDVESANRQILTLPEPRQAPSKRSLDECKDVGSMLSDKCDIGYEIAK
CLYFSNPKLVILIVFDLTPQFRVSDRTWIKMGKIKLEPTEEEGNSIVIKEEELTYEEKLENVSVIA
KPMASKKLTKKCHKLIKKAAKHKTFVRNGLKDVQSRIRKGETGIVLLAGDVTPIEIMCHIPGVCED
KSIPYCYIPSRTDLGASLGVKRGSLMVLIREHESYIDVYNECKEEITNLAVEL
>abi19709.t1_1
MPKTVFIRTILYCGESLDARKERIRKYREECIAETKVDPQLIDKADGGEFSDTRELQCFAKCFYTK
AGFITKDGELLLDVVKAKIPPEQDRETALGIIEKCKSLKGKDVCETAYAIHKCYFQNAHAAGKKE
>abi20726.t1_1
MEPQSIVTVLFLCAFILTEQVLGENSSEKLHKKVCMREANVNPSLIEKACYYLESGFINEAGEIQI
DEIKSKIPRKVNGKLAQEAIDKCKNTKGMDSCETAFEMQKCLHANRLTV
>abi21550.t1_1
ALTDEQKEKLKQHYKECFAETGVAEEVVQKGRKGEFIDDPKLKQFVFCFFKKVGFQNEQGDLQLDV
IKAKVPTDVNKNEADDVIKACKDTTGTDAADKAFNVYKCYWKSTPNHVSLI
>abi22385.t1_1
MKYLLIFIAVAVVVVSGVPQSQKDKIKAFSRECISVTGVDKELLKQAKEGHFVDDEKLEDFTFCLF
KKMGLQDENGEILINVARSQLPAELSEADKDDIVNKCFSKKGVTNX
>hom6835.t1_1
MIYHLNILFCHFADEILTSELQELLSGNVYGNVRVRRDDEAAKCQSKHPLSGLKSCCSNDIVDHYH
EADRPIMLECRNELYGKENQSDVPRECGNVHANKDLYLIDSNGKLLPDKLRNSIKEKLSKIQWLQP
ELDTVINKCIGEVESANKESSEEGKCKPGASTIGHCVWREIQFACPAEEITDVENCEKIRNYLNTK
PQIP
>hom15093.t1_1
MKAAAKLIRNTCRQKTKATDEQIDNMHKGIWDEDQNAKMKKNGAFDLELANKQLPQLPENRKEPTV
NSMNKCKDAGGDKTEKCDLSYAFFKCMYFDNPEVTKDGHMDVESANRQILTLPEPRQAPSKRSLDE
CKDVGSMLSDKCDIGYEIAKCLYFSNPKVSKWFYP
>hom18384.t1_1
MEPQSIVTVLFLCAFILTEQVLGENSSEKLHKKVCMREANVNPSLIEKAYSGDFANNRELKCFFRC
YYLESGFINEAGEIQIDEIKSKIPRKVNGKLAQEAIDKCKNTKGMDSCETAFEMQKCLHANRLTV
```

Troponin C

```
>abi680.t1_1
MENKIDKKFLKNYFLCKCLRRSTYSTRQTGQEEPESEGRITQIADDVPPTTPKSRHLLAIEEISDV
GARQNKPRHSVVSKSQMKEFREAFRLFDKDGDGSITKEELGRFMRSLGQFARTEELQQMLQEVDVD
GDGNVSFEEFVDIAWSAGAGAGPEHVLSREEEEKELRDAFRVFDKHNRGYITASDLRAVLQCLGED
LSEEEIEDMIKEVDVDGDGRIDFYEFVNALGEPGNDDSYDDEDDEIAAF
>abi1280.t1_1
MTGRGKVITGRIETEIEKNREESNWAKVIDLAEQLKEKSPEYEYLSHFLIGEGRLERYLEEWAPIE
ANVNKAKYNLMEARRNLLIASNKKGEKFGVALDAHLLLGKLYYACGNYNEGLNSFKSAELHGLSEK
TLPLRSLKIVAESFAIKGLCLQKDTSATTKFKKAERHDEMLKCFEVASDLGLLYMQEVDKLHISTL
TNTGTHSPQPPIVGKSLSSVLEQAMQEAPALLLQHNKHQEALNRYRNMLTAVESQGIYTVRLKFLC
QLAELLLQEIPSDVYKPPNNLNVTSPWKPKQYASLNQFIPRNKCEETLLVLLVAESIAVRNAVLSQ
SPEFKEARHSAYQDAAAVYDLLTIATVRWGQISLLQESLERAMKFSFKEPHLWRQHALSLLAMGHY
EHAILVLKEVIRLEPNSSVNCLIAAKLCYEHLNMPSEGTKFSEDAMKIELVHSSALLSRCHLYIGI
GYQLQAQMTLIKQDKSNLNNMALENFKCAVQIEPNDNLCEYYLALQLAIVGQIDDALHHVQIALNL
QPESSSTLHLLTLLLSANRQHEEALQMVEAALQEYPDCLNLMYVKAHLELHEIGGEKALTTAKQML
ELWKNLYEGQTGSDVPECDRKSDTRSVFQLYTSEMSDKDTSSLQAQSIAASRIEQALSEVASSMSS
FSPRPGPQRAWMLLLEIWLLLAELYLALDQLPDVLKCIQEATQIFPLSHHIMHMKGLLHMHKQEWA
EAKLCFQNAVAINPQHVKSLQQLGLVYHYLSLQGLAETTLREAAKIDPGNHVTWYNLGKVLEALGE
YEKASDSMATALMKVIADYRCQDLQKSRTRKSSVFDATRPLAQAACKRRFVARLTHCRYAATNRNA
LCAANCQNVRPTVTGLLGRRDALRISSWPPDVSKKVSCTRNTLRLSIGPGMFFTADQLTEEQIAEF
KEAFSLFDKDGDGTITTKELGTVMRSLGQNPTEAELQDMINEVDADGE
>abi1281.t1_1
MFSNRNGTIDFPEFLTMMARKMKDTDSEEEIREAFRVFDKDGNGFISAAELRHVMTNLGEKLTDEE
VDEMIREADIDGDGQVNYEGAFNF
>abi2787.t1_1
XAEIQEWYKGFLKDCPSGHLSVDEFKKIYGNFFPYGDASKFAEHVFRTFDANGDGTIDFREFLCAL
SVTSRGKLEQKLKWAFSMYDLDGNGYISRQEMLEIVTVCIIFFT
>abi2946.t1_1
MGSFGFIFGRNDQNGIRRNALLAEASVKIATRTGAGVQNQVTFVIEQMPALIYLMLFFYAANVKQQ
IAQSRGAITCCSSVLEIAHCIFSRFTPRRFSCPTIKRFIARFDAGRRALGFNAKEDFEEGGFILYI
QDGTSAVSVEFPRKDRRKNGKWYVEDDQKQAILRKAFQMFDTTKSGTIETLKISTILNTMGQLFDD
SELNKLIAENDPDKSGKVDFDGFCNIASHFLEEDDSESTTQELKEAFRLYDREGNGYITTATLKEI
LRALDDNLSSSDLDGIIAEIDTDGSGTVDFDVVFILLQNLNYKTYKASRLKSIMRRLVTSKISSSV
IRHRKYPAFCTPSSPRLSFREYVASGWFFVVVGSFEYHTGILHIFGEMDEEQKLTILRKAFQMFDT
SKSGMIETTKISTILNTIGQLFDHGELNKLIAENDTEKVGKVSFEGFCSIATHFLDEEDDESTQQE
LKEAFRMYDREGNGYITTATLKEILAALDDKLTSRELDGIIAEIDTDGSGTVDFDGKYWEF
>abi3138.t1_1
MSSRKTVSRRGTTKKRAQRATSNVFAMFDQAQIAEFKEAFNMIDQNHDGFVDKEDLHDMLASLGKN
PTDDYLDGMMNEAPGPINFTMFLTLFGERLQGTDPEDVIKNAFGCFDEENMGVINEERLRELLTTM
GDRFTDDEVDEMYREAPIKGGLFDYVEFTRILKHGAKDKDEQ
>abi4789.t1_1
MLQGAKIYSKQLLYKASGKVKRGKNGSRLQSYIRSLSLPSWPVAGADKEKKVKKKKKEDAPAADAA
PAPAPAAETRSSSKGSSKKAKRSGSNVFSMFSQHQVAEFKEAFQLMDNDKDGIIGKADLRATFDAV
GKLSNEKELDEMINEAPGPINFTQLLSLFALRMQDSGGSDDDDVVVAAFRSFDENGTIDSERFRHA
LMTWGDKFSSKEVDDAFDAMEIDDHGRIDTNALISLLTGAEEEGEGEAA
>abi11482.t1_1
MELCSNFDADEIKRLGKRFRKLDLDNSGALSIDEFMSLPELQQNPLVQRVIDIFDADGNGEVDFRE
FIQGVSQFSVKGDKLSKLRFAFRIYDMDNDGFISNGELFQVLKMMVGNNLKDTQLQQIVDKTILFA
DKDEDGKISFEEFCSVVGNTDIHKKMVVDV
>abi11947.t1_1
MLRNRRLYKIYICKPQRFDPNVSVSVLKKAFEAFDSQKSGSIPCDMVGDILRLMGQPYDKKILQEL
IEEVDADKSGRLEFEEFVTLAAKFIVEEDDEAMQKELKEAFRLYDKEGNGYIKTSCLKEILRELDD
QLTDRELDMMIEEIDTDGSGTVDFDGKLHQPLLK
>abi12033.t1_1
MSGVNITCNIGNQLGNGGGEMKFNVASKDLPSLATCIATEIVNEILENALDIATMGSVTGLECDTI
AFSVDENMCKFSSNETYQESAEKFNLQSNSISNFTNDIDETCPGIKEIIDGLSSIHVPNQNAEEPM
RLDVEMKRLLSEMMSPPYDIVNYETLPRKDGVGDQPIEEASLIEESANKSDECLVDTEKTKIVEKK
TRVTDLVMNSKQVLSEYLMSEESRKQDEWEESNEQVLQVIELDYIRPDEEQFHAHTSILPENVML
```

PDDDLFSPLLDDDCMDNIDLSSTAFKDEERQISLEKLVGGGDDYNDDKEQDEKENVAGSSHSKPKD
DNGVSNVTDVNKEEKSKSKFQILKKKKFNFNFKFWGLGKKKRGQEKAQLYDTLSVYLTIYIKIRSG
IDADADVAKVRSGNGTIDAVNIGDCLRALNQNPTLAIIEKVGGTKKKNEKKLKLDEFLPIYSQVKK
DKEQGNFEDFLEALKLYDKDENGKMLSAELSNMFLALGEKLTDSETDEIIKDCMEKEDDDGFTPYE
LNLIENNILQFSSFSSSS
>abi12427.t1_1
MLFDKDEDGTITMAELGVVMRSLGQRPTETELRDMVNEVDQDGNGTIEFNEFLQMMSKKMKDADGE
EELKEAFRVFDKNNDGLISSNELRHVMTSLGERLSEEEVEDMIKEADIDGDGQVNYEGK
>abi12615.t1_1
METNSENNLNFQEFKAVRGLRQRSQDFVQNLSILSRGSLDEKLRWTFNLYDINGDGYITREEMTEI
VTAIYDLMGKLAEPSIDDDTVKEKVDRIFQKMDKNQDGVVTFEEFLDCCHKDKDISSSMTVFDSSI
>abi12695.t1_1
MANYSEDQMAEFQEAFQLFDNRGDGKIHVSQIGDALRALGQNPTESDVKKYTHQHKSDERVSFEVF
LPIYQQISKSRSADTADDFIEGLRHFDKDGNGYISSAELRHLLTTLGEKLTDDEVEQLLQGQEDSQ
GNVNYEDFVRLIMSG
>abi13233.t1_1
XLRTAFVMLDSNSDGKVTTDDLQIMLRRLGIEIKDEIIEELVRTASHTGSELIDENDFLQWVKRIQ
ELLPEKATDDINKDLMAAFQVFDLDSNGFISRDELKIAMEKIGEPVSDEQITQLITMADIDMDGKI
NYEGKD
>abi15179.t1_1
MEPQPEEIIVPINNDLERKIADVFDIFDHAGTKTVDVREVGTIVRALGCCPSEAEVQEMLVAMEDP
ALPGSIHLVKFLPYVAQMITEHKHQPATPDLLLEAFQLLDADGRGFLTREYISTLMTQDGEPFTQD
ELDEMLEIAIDPHTQTVPYEYYINQLMHEPLSEDDVYAIADEIEAAKPPPPPPPKRFSEVLHTMQA
ATT
>abi15782.t1_1
MTHFQSAMPNRDFLWSVFQRVDRDRSGFINADELQVALSNGTWSPFNPETVRLMIGMFDRHSRGQV
SFEDFGALWKYVTDWQNCFRSFDKDNSGNIDKDELRTALTTFGYRLSEGLIDILMRKFDRHGRGTI
LFDDFIQCCIVLYTLTSSFRQYDTDQDGVITIHYEQFLRLFILIILWISTLGAFVYSIRAQSNIGW
IVLVAATVITIILVIIPLDAKEESQEFVKLNNDDFLSKVMNSDNPVIVNFHAEWCEPCHILTPKLT
ELIQHKENIDLATVDVELHAELAHTFEVKAVPAVLAVRNGLVVDKFIGLVDANMIENMISKLDTKK
T
>abi16665.t1_1
MRENGIVPDVRQDSKTGNMSPIRGIGTTYDPVDRKRLWALERVQERFLKEALRLEKSTPAYIAEGA
RDELGEEELKNVRVLRIKSRGGSGLLNFDAFCRVASHFLESEDDEALQKELKEAFRLYDKEGKVLH
DV
>abi17145.t1_1
MTDSQSVNVRVPLESMEKLPELKENPFCTRICKVFSRDGKGDLSFEDFLDLLSVFSEQAPRDIKVY
YAFRIYDFDGDRHIGPDDLNQAVRLLTRHELSPEDMQQIVEKVIEEGDVDGDGKLSYMEFEHVITR
APDFVSNFHIRM
>abi17600.t1_1
MFGILTIELLINTNVTPINVKCSQHLKLCAYTHANRSWIVYVICSVYGYLHNLGMECVQVLCKYCV
AVARLCGGAHAFSTTELCALYNTKISAQIESELNTHSDFRRHLRKAGTSIDNIEDDFRNGLKLMLL
LEVISGETLPKPDRGKMRFHKIANVNKALDYIASKGVKLVSIGAEEIVDGNLKMTLGMIWTIILRF
AIQDISVEEMTAKEGLLLWCQRKTAPYKNVNVQNFHLSFKDGLAFCALIHRHRPDLIDYNKLSKDN
PLENLNTAFDVAEKYLDIPRMLDPDDLQNTAMPDERAVMTYVSSYYHCFSGAQKAETAANRICKVL
KVNQENERLMEEYERLASDLLEWIRRTMPWLGSRQTDNSLAGVQKKLEEYRTYRRKHKPPRVEQKA
KLETNFNTLQTKLRLSNRPAYMPTEGKMVSDIANAWKGLEMSEKAFEEWLLSEMMRLERLEHLAQK
FKHKADAHEDWTRGKEEMLQSQDFRQCRLNELKALKKKHEAFESDLAAHQDRVEQIAAIAQELNTL
EYHDSVSVNARCQRICDQWDRLGALTQRRRQALDDAERILEKIDILHLEFAKRAAPFNNWLDGTRE
DLVDIFIVHTVEEIQGLIDAHAHFKATLGEADKEYQSIVGLVREVETIVKQHQVPGGLENPYTTLT
AHDLTRKWADVRQLVPQRDATLQAELRKQQNNEMLRRQFAEKANAVGPWIERQLDGVTAIGMGLYG
NLEDQLHRLKEFEQGVYAYKPHIEELERIHQAVQESMIFENRYTQYTMETLRVGWEQLLTSINRNI
NEVENQILTRDSKGITQEQLNEFRSSFNHFDKNRTGRLTPEEFKSCLVSLGYSIGKDRQGEIDFQR
ILAIVDPNSTGYVHFDAFLDFMTRESTDTDTAEQVIDSFRILAADKPYILPDELRRELPPDQAEYC
IQRMPPFKGLGAVPGALDYMSFSTALYGQSDL
>abi18614.t1_1
MRLLGPDLHTDDVLKKAFDTFDVEKKGSIGTAMVGTILGMLGIQVTEKTLGEIIAEVDADGSGELE
FEEFITLASRFMVEEDAEAMQAELKEAFRLYDKEGNGYITTDVLREILKELDDKITAEELDMMIME
IDSDGSGTVDFDEFMEVMTGGDD
>abi20122.t1_1

MASDTGFKKPIGAKKKGSGPKFELTEEQKVDIREAFDLFDTEGIGKIDTKELKVAIRALGFEPKKE
EIKRMIADVDKEGSGKLSFDDFLQLMAVKMAEKDSKEEILKAFRLFDDDETGKISFKNLKRVAREL
GENLTDEELQEMIDEADRDGDGEINQEEFLRIMKKTSLY
>hom1130.t1_1
MTGRGKVITGRIETEIEKNREESNWAKVIDLAEQLKEKSPEYEYLSHFLIGEGRLERYLEEWAPIE
ANVNKAKYNLMEARRNLLIASNKKGEKFGVALDAHLLLGKLYYACGNYNEGLNSFKSAELHGLSEK
TLPLRSLKIVAESFAIKGLCLQKDTSATTKFKKAERHDEMLKCFEVASDLGLLYMQEVDKLHISTL
TNTGTHSPQPPIVGKSLSSVLEQAMQEAPALLLQHNKHQEALNRYRNMLTAVESQGIYTVRLKFLC
QLAELLLQEIPSDVYKPPNNLNVTSPWKPKQYASLNQFIPRNKCEETLLVLLVAESIAVRNAVLSQ
SPEFKEARHSAYQDAAAVYDLLTIATVRWGQISLLQESLERAMKFSFKEPHLWRQHALSLLAMGHY
EHAILVLKEVIRLEPNSSVNCLIAAKLCYEHLNMPSEGTKFSEDAMKIELVHSSALLSRCHLYIGI
GYQLQAQMTLIKQDKSNLNNMALENFKCAVQIEPNDNLCEYYLALQLAIVGQIDDALHHVQIALNL
QPESSSTLHLLTLLLSANRQHEEALQMVEAALQEYPDCLNLMYVKAHLELHEIGGEKALTTAKQML
ELWKNLYEGQTGSDVPECDRKSDTRSVFQLYTSEMSDKDTSSLQAQSIAASRIEQALSEVASSMSS
FSPRPGPQRAWMLLLEIWLLLAELYLALDQLPDVLKCIQEATQIFPLSHHIMHMKGLLHMHKQEWA
EAKLCFQNAVAINPQHVKSLQQLGLVYHYLSLQGLAETTLREAAKIDPGNHVTWYNLGKVLEALGE
YEKASDSMATALMAANCQNVRPTVTGLLGRRDALRISSWPPDVSKKVSCTRNTLRLSIGPGMFFTA
DQLTEEQIAEFKEAFSLFDKDGDGTITTKELGTVMRSLGQNPTEAELQDMINEVDADGE
>hom2625.t1_1
MGSFGFIFGRNDQNGIRRNALLAEASVKIATRTGAGVQNQVTFVIEQMPALIYLMLFEDDQKQAIL
RKAFQMFDTTKSGTIETLKISTILNTMGQLFDDSELNKLIAENDPDKSGKVDFDGFCNIASHFLEE
DDSESTTQELKEAFRLYDREGNGYITTATLKEILRALDDNLSSSDLDGIIAEIDTDGSGTVDFDVF
LLVFSDLKQTKTSESIDEEQKLTILRKAFQMFDTSKSGMIETTKISTILNTIGQLFDHGELNKLIA
ENDTEKVGKVSFEGFCSIATHFLDEEDDESTQQELKEAFRLVGWLVTTYVAKKKLLRMYDREGNGY
ITTATLKEILAALDDKLTSRELDGIIAEIDTDGSGTVDFDGKYWEF
>hom10140.t1_1
MIIFRVTKVLCPWSYAQIVIDADEIKRLGKRFRKLDLDNSGALSIDEFMSLPELQQNPLVQRVIDI
FDADGNGEVDFREFIQGVSQFSVKGDKLSKLRFAFRIYDMDNDGFISNGELFQVLKMMVGNNLKDT
QLQQIVDKTILFADKDEDGKISFEEFCSVVGNTDIHKKMVVDV
>hom10640.t1_1
MSGVNITCNIGNQLGNGGGEMKFNVASKDLPSLATCIATEIVNEILENALDIATMGSVTGLECDTI
AFSVDENMCKFSSNETYQESAEKFNLQSNSISNFTNDIDETCPGIKEIIDGLSSIHVPNQNAEEPM
RLDVEMKRLLSEMMSPPYDIVNYETLPRKDGVGDQPIEEASLIEESANKSDECLVDTEKTKIVEKK
TRVTDLVMNSKQVLSEYLMSEESRKQQDEWEESNEQVLQVIELDYIRPDEEQFHAHTSILPENVML
PDDDLFSPLLDDDCMDNIDLSSTAFKDEERQISLEKLVGGGDDYNDDKEQDEKENVAGSSHSKPKD
DNGVSNVTDVNKEEKSKRASFVFSIYDFEGNGTIDAVNIGDCLRALNQNPTLAIIEKVGGTKKKNE
KKLKLDEFLPIYSQVKKDKEQGNFEDFLEALKLYDKDENGKMLSAELSNMFLALGEKLTDSETDEI
IKDCMEKEDDDGFTPYERKSYKSYK
>hom10988.t1_1
MSRDSHYRTQFKEAFMLFDKDEDGTITMAELGVVMRSLGQRPTETELRDMVNEVDQDGNGTIEFNE
FLQMMSKKMKDADGEEELKEAFRVFDKNNDGLISSNELRHVMTSLGERLSEEEVEDMIKEADIDGD
GQVNYEGK
>hom14744.t1_1
MRENGIVPDVRQDSKTGNMSPIRGIGTTYDPVDRKRLWACSGLLNFDAFCRVASHFLESEDDEALQ
KELKEAFRLYDKEGKVLHDV
>hom15614.t1_1
MFGILTIELLINTNVTPINVKCSQHLKLCAYTHANRSWIVYVICSVYGYLHNLGMECVQVLCKYCV
AVARLCGGAHAFSTTELCALYNTKISAQIESELNTHSDFRRHLRKAGTSIDNIEDDFRNGLKLMLL
LEVISGETLPKPDRGKMRFHKIANVNKALDYIASKGVKLVSIGAEEIVDGNLKMTLGMIWTIILRF
AIQDISVEEMTAKEGLLLWCQRKTAPYKNVNVQNFHLSFKDGLAFCALIHRHRPDLIDYNKLSKDN
PLENLNTAFDVAEKYLDIPRMLDPDDLINTPKPDERAIMTYVSCYYHAFQGAQQAETAANRICKVL
KVNQENERLMEEYERLASDLLEWIRRTMPWLGSRQTDNSLAGVQKKLEEYRTYRRKHKPPRVEQKA
KLETNFNTLQTKLRLSNRPAYMPTEGKMVSDIANAWKGLEMSEKAFEEWLLSEMMRLERLEHLAQK
FKHKADAHEDWTRGKEEMLQSQDFRQCRLNELKALKKKHEAFESDLAAHQDRVEQIAAIAQELNTL
EYHDSVSVNARCQRICDQWDRLGALTQRRRQALDDAERILEKIDILHLEFAKRAAPFNNWLDGTRE
DLVDIFIVHTVEEIQGLIDAHAHFKATLGEADKEYQSIVGLVREVETIVKQHQVPGGLENPYTTLT
AHDLTRKWADVRQLVPQRDATLQAELRKQQNNEMLRRQFAEKANAVGPWIERQLDGVTAIGMGLYG
NLEDQLHRLKEFEQGVYAYKPHIEELERIHQAVQESMIFENRYTQYTMETLRVGWEQLLTSINRNI
NEVENQILTRDSKGITQEQLNEFRSSFNHFDKNRTGRLTPEEFKSCLVSLGYSIGKDRQGEIDFQR

ILAIVDPNSTGYVHFDAFLDFMTRESTDTDTAEQVIDSFRILAADKPYILPDELRRELPPDQAEYC
IQRMPPFKGLGAVPGALDYMSFSTALYGQSDL
>hom16577.t1_1
MRLLGPDLHTDDVLKKAFDTFDVEKKGSIGTAMVGTILGMLGIQVTEKTLGEIIAEVDADGSGELE
FEEFITLASRFMVEEDAEAMQAELKEAFRLYDKEGNGYITTDVLREILKELDDKITAEELDMMIME
IDSDGSGTVDFDGNFIFLIF

Vitellogenin

>abi441.t1_1
MTRYIGLAAASDNAWKSNTEYVYNVRGRTLSGLHDVADEYSGIVMKARLMIRPKQEGLLTARLTDV
KYAQVHTHLPKGWDMEMRESELNYQQMPLTEEPFQIKINNDVVEDVICSKNIPNWEANIIKSIVSQ
IQLDINAQNVVKSHVNQLPEEDSYSAVFKTMEDTVTGKYETMYNINILPKYILQSEPWLAPFPDMK
GNGEIVEVVKNKNFSRNDELAGYHFGFSRMGEWEPNTNKMGNFFSRSSVSRAIISGTLKRYTIQSS
VTTNKIIISPTLNNEQKGMVVSRLNITLMNVQDASNPFPEPSQPRNVGSLAYRYNNPFGEDNTVRA
GEPERETRQHHNSPYGSSSSSSSSSSSSSEEDQTKPQQQHSRYRRSSPQNQQSSGSSSSSSSSSSS
SVSSEEYWQQEQPKLNSPPDVPLLPFFVGHKGRSIKYAPNVDVVKTAHKLCKEIGKELQHPQEMYK
EQTLDKFTLLTSLLRIMSEKEIQEVGQSLYNNNERGEKSDVWKTYRDAVAECGTGPAFVNIQNWIK
NKEIKGEEAAEIVASMLQNVREPTDEYIRKFYAMATRPDVQSQAYLNDTAILSFSNLLRKVYVSRK
MSHNQFPVHVFGSFRTKAGKKFVTEEYIPYLNKKLNESISHADSHKILVYIRALGNLGHQQILTAF
EPYLEGRKQVSQFQRLQMVLALDKLVKVEPKVARSVLFKIYQNSGEEEELRAAAVFQLMRTVPPAS
MLQHMAKYTGIDKSEYVNAAVKTAIQSAAQLEGEEFEVLIIKAITEIYNETWIRFALLEKEVLIEL
RDCK
>abi15563.t1_1
MDGTGRTHVIELGLLGPITLYYEFSLHRIFWSDAGTGNIESTSIEGDDRHGFRSLRSNPVGLASLS
KDIFWTNQRSSKIYWADKNNGGYNRKITLDLPEDIDGMRIISISPRPIPSHPCRQNNGNCTHLCLA
VEKSAQCACPLGMNLKVDNVTCFKPKTCAATEFFCKNSETCISRDMLCNGHKDCLMGEDEVDCDVH
NDCLHNEFQCKNGECIKNKFVCDMHYDCKDKTDEMVCSDDKYKRKCPENHFRCSNDLCIAERFVCD
GVKDCYDGDDELINCVSTTCSTEQFRCTIGSCIPKSWECDHEYDCSDLSDEHSGCGSITCSPEKFT
CNNGRCIDKILQCDKVDDCGDMSDEISCKISDRNGCKLNEFQCRGNKTICIPESALCNGTPECPLH
DDEQGCSNCRPDEFECDNKICLTKDWICDGTDDCGDGSDESLNICAYKNHSLLVPSADVPCPHGFR
CKSGQCINIRLLCNGEENCFDGSDEHGACNTSCQVNSNPCSHKCIKTPSGPMCACETGYKLRGDGQ
TCVDIKECSFEPPICSQLCTEQLGSYTCGCFNGYVLRADKISCKSKGEPMSMVFTVNNEIRQLSKA
NNTLSILYSDDSPSISGLDISIELGYIYFSIEQTSTIHRINLKNFSREYLTNVGKPQKLAVDWITQ
NIYFVDGSDKTIRVCNFHQQLCSKLFSLDSNSQISAITVDAVNKHIFYSVTSWWIFNSPNSVIYKC
NLDGSRLQELLKSTKGYITGLAYDIYRKKIFYVDKHNGQINQINYDGNNQMNLIYNLSKPYGLNFF
EDNLYYLMNGGLMGKCKLFGDERSCEAFRLNTQSGELFSILQSSRQPNVGNVCQNNNCTQMCIPTD
VRPKCLCFEGNYVLEGQECKLERREAHLPDKKPLFKPHVTNQKSSIENSSAGSMVAGVLIPIFILL
GAAVVYLFIRRRNSGKFNISMRFYNPMFGISSNNEAPGTVLQVGEHEYSNPVDFCSQDMNAATNAI
LQKVNPLVNIDG
>abi17397.t1_1
MTSSVNLYSGSCIPSNKRCDLKWDCDDGSDETSDCSAMVQGDCPEGEFQCKLTRRCIFHGWWCDGD
PDCGTSAELGPDMSDEVNCKMDTFWCPWNQGRCGNSTVCRPLKVFCDGHNDCPDNSDEFTEFCNNK
SRCDNFGCSHECSMTQDGPRCYCPEGRTAPGRTCVDVDECVLEDSCAQNCVNTVGSFECSCVSGYK
AIGHDCHAINGKDFLLPKKCINYLSQNQLKMIYVHQTPLVVWCAFHTVLGVMNSPFIISVPESEEP
SLLFSTEHTIERIDLKGKLWSNKTKTTLMNITALNFFHRNRTLYYLQHKITNSSLMAVNVDNFSQT
WELGMSHLFKNLQSVKQIAFDWVSENWYLLDVQQEVIILCTTHLDKCIILLESKMSTPRDLALDPT
SGYFFFTKFSERFPSMIERCRMDGTERVTLVDSNIVFPCSITLDFATKKVYWVDNWLDGVGYVDYD
GKNRRIIKEDPLYGSLYAITFLESKWFVSNNKVMVNKIKVVDKFTRKSNILIDNTTMPLHLHVFHR
QRQPDVAHPCKNNNGNCEHFCIPNWNRDIAIAKCMCLAGYKLQERGRCIQQRPSTFLLMAKMKSAL
IKGIDLENGTEAIAPITGLSQLSGIEVHVKNRTIFYWNGTVIEGYNLDTHIRTKMVESGMSLMHAG
VAYDWTLGNLYWTQSDSGTISVVKLSDPTLHRTLYFKRASHPTTIVLNPKRGLMYWIDRPPARLTD
GKIRSAWMDGTNVEIFIDTDIRWPSGLSLDLVGKRLYWSDADLGKIESASLDNKNRKVIFTNLIYP
CEVVYFNRSIYYTEYQKGTIISKNLDTNVTRTLVEDAPISQLRLFNSKSQTGADSYLNDTHCVHLS
LKIPNRVVCKCGDGYNFVDSTCVRQKNFSLCREDEFQCATYVRCIPKKLVCDGDRNCRDGSDELTA
DGGPCLRNCSSSEFTCASDGTCVMKHWLCDGEKDCADGSDEEPKNCPGLCLPMQFLCAKTKRCIPY
AWKCDGDSDCGLLDDSDEGEDCKVKKCDITEFTCHNNRCIPTDLYCDGADDCHDGSDEIDCAICDP
TLHFFCEPSKSCLPNTLRCDNHTDCADGSDERDCKSLCEKSEFECSNHICVPAVFRCDTFQDCLDG
SDERNCNYTKDHPLVSSTEKSSQLESNCEHPSRLCDNNTRCVPLEHLCDSKYDCLDESDEGLRCLE
DLCSLGSICSHLCHNAPEGVVCSCPDDLHLQPDMTTCLHVHPCESWGVCSQLCTRHGSRHKCDCLP
GYVIEKDGFTCKSENASTPYVIFSNRHELRGVDLYSFNVKALISSLKNTIALDFYHDANSDMIFWT
DVIDDKIYRGTLVGNYLSNIEVVVHSGLSTAEGLAVDWIGENLYWVESNLDQIEVARLNGSFRRTL
VGGEMDSPRAIALDPRDGLLFWTDWDNSAPRIERCSMAGLDRQIIVRVDQITDGAWPNGITLDYDM
RRIYWIDARSDSIHTTKYDGTDLHEVMKHHDMLSHPFAISLFENYVYWTDWRTNSVVRANKWNGGD
VFVIQRTLTQPFDIQILHPSRQPRDGLSPCGNNNGGCSHLCLLHTNHTYRCDCPHVMRLHTDNQTC
IVNERVLLIARNNEIRGVDLLQPYYHTIPTISFQNAMNNRHLEYLAENSTLYWIDSQQNEVKRTGL
TSGPAHTLIDTGLQHPTGLALDWLAGLIFIGSSKGITVCNLDGEYAMTLIEHISVGSIAVDPTLGR

LYWVMENNDNITIDSSAMDGSKHITLVEDLDRTTSGLTVDRESQRLYWISDFNVYFYDFNAENVTK
LNLSGAVSAATVYRGLLYYADDDTQSIHSTNKTTGEDSKILRNSTGVLTLRIYDREEQIGIGPCGK
TKTGCQHLCLPISATERTCRCATGYQVDPQDPTKCIGLSEFLFYSNGWELCGRSLDDSNSTALGPL
SRVALASAIDFVAEEDLLFWADNDRGTITSIKRDGTGRKLIIDQHDGMESVSADWLTGLAIDWSAR
NIYWSDPKHGVIQVAKLNGSSRYVVLSHEIGTPSALAVDPEVGLLVWGGASKLETSGLDGSNRKLL
VDQARSISDLTLDYVNHYIYWCDSGSDTIERMKYDGSERLTLLNHSLENPIALTVFNDVLYWIDST
HQHGSIKLAPVSNLSDYTVLINNTGDSPKDIQIFSKRRQRGVNPCAQNNGGCEQLCLFNGNSPVCA
CLHGKVSENGKSCEEQGSVEGVSFEQLHQALYWTCNNDATISRVNLTKDGKNASNVEVVVRLRSQD
KPRGIAIDSCGSYLYWTNWNSQQPSIERVFLSGYGRENIISTEIRMPNAITLDHKAQKLYWSDARL
DKIERCEYDGKDRQVLGGVTPHHPFALAVYGDYLYWTDWVLHAVLRADKLTGSNVVSLRRDVAKPM
GIIAVANDTDDCFSNPCLELNGGCEDTCNLSPMGEVRCSCDENRILGEDARRCFGRTTQTCNEDSF
RCSDGGCVPLQVTCDKIPHCTDASDEEAGYCAHRVCPTGWFSCTNRRCLKSKDHCNGVDDCGDASD
EANCACSEQNHFKCNSGRCILKQFRCDKDPDCEDASDEIGCGERNCAEENKDMNFAQCPNTVACIH
INWWCDGQNDCWDNADESNCTICQQDVQFKCTGNSKCIDAEKQCDGIDDCGDGHGRLSSDEQSCHG
NCTKDQFKCLSDLLCIESVRKCDGIENCQDGSDEHGCSVECPYDRFRCHSGECIPQAWQCDGQGDC
RDSSDESAHCMTRYCLSSEIRCNSTGGCITPHQLCDGEDDCADGEDERPTGGCLGKRCSTQEFQCD
DGRCINMEYYCDGDDECDDGSDEPEGCHERCEFKCKTFEHCILKEQVCNGVADCIDGSDEGRHCNN
EEEQCIKEGLFKCSNGVCINETLVCNRENDCGDFSDEISCNINECTATIKPCSQKCIDKPIGYQCA
CEIGYELDPNDAQNCVDVDECAEYPCSQICQNTMGSYHCMCGSNYFLREDKKSCAADSHDIPKLIV
ANRYYIRELTLHGNATLLIHKLTNVVALDFDWKTQCYFWSEVTGGKSNIAKQCNLTDDNANITILH
SDTLQNPDGLAGLDTIEVSTLGGQNRRVLISTGLEEPRGIAVHPLKGYLFWSDWGSNVHIGKAGMD
GSNPHVIINKVLGWPNAIAIEFETNELFFADAKEDYIGVSDLDGRNVRIIASRLKYPDLKLHHVFS
ITLWEDYVYWTDWEMKSVERVHKNHGNMTSSLLTTIHRPMDLRMVHPFRQPQRDNPCEKANCSALC
LLTPEAPFYKCACPENYILLPDNRTCKSNCTSMHFECPHSYKCIPFWWKCDGQDDCFDGSDEPADC
RPFKCNPGQYQCDDGNCIHPSNICDGTSDCPDKSDEKNCNDYACLVTHFRCKGNATTPPRCIPGNL
RCDNKIDCPFGGEDEEGCPTASCPPQHFQCDNKKCIPSVWVCDKDNDCGDKSDEFETCTTRTCQRG
SFTCKSGRCIPLTWVCDGDGEDEPITCNQPGFHTCDPSYFRCNNSKCIPGRWYCDYDDDCGDNSDE
FNCEPRECSESEFKCKDGRCIQKIHVCDGEYHCKDKSDEMNCSATLCEADQFKCNDSSNCIPVTWR
CDNDLDCIDNSDELSCTESMSCQGTGQFHCDNGRCVDGRWRCDGEDDCGDNSDEKISQCKRLKCPR
GLHKCIENHRCVLRATLCDGHCGELGTEDAIYCRNVTHCNDKNQFRCTNEHCIDRNLVCDNKNDCE
DNSDEVNCICKWNTCSHICLLDKKGAPKCKCSLGFKHNATTNSCEAHEQNATLILAIENELRLISP
YQTNSEKALLGNTELGMGVGYKIGAVDVFYENQDIIIFWTGYRHKQLEKMLLRLNNSSRTSRDISS
RTILVDDLIEPYDLAVDWVARRIYLTGTDKVLVTTFDGKQKYTLINDGTRQRGDIVVAPTQGLLFW
VDLGSPAVIETAYMDGNKRRVLVNDVIWPTGLAIDHPAQRLYWVDQKMHRVESVKFDGSDRQIVHI
FDDGKKPHKIDVFEDYLYISTSDTHDVLKMHKFGNGKIHYLSEGLLTLHDIFVVHEHRQEHGIKNR
CQDFCHSSEFCLLSSGGATCFCADGLKKNNLTCSAIMECPLNCNQGKCEWIEGIPTCTCPPEYSGT
LCEHYRCSLYCHNGGICYVDKDAVGWPLRCHCPAQWTGEKCEITVNMCEGPCYNGGTCYRYHRTGL
GHRCKCKDGFTGVRCENCGNHLKCNNGGVCTHEDNHEYCKCPLGYGGSNCERFECIEDVCNKRGTC
YISSAGTTCRCNSGYYGEHCENESCVPSCQNGATCVMGARQAECKCPPTFGGQSCEHDLCMNPTPP
KECAANGRCYCRNNGTCRTMAGTTVCDCPHMWGGEICEKYIGENSACNIYCLNGGDCTLTINNEPT
CVCKDGYSGKRCEIFRACPLDCQNGGSCAISDTGPYCQCTHEYEGPNCEHSSLDESDVRHRMDKED
GGVVLPVLLAFGVILLVVGCIGIAYLILKKRRPFSHERLQENDFNNPMYQDRDAEPFSLDADKSTN
FVNPVYETVYNGAGSAKDEKTGLLQSNPDEIPPVRNEET
>abi17706.t1_1
MKKLCWTNNTDETIQCIEYDGFETKNKAVVLTGVFSTEGLACDWFTNKLYWTESNLHHIEVVSMVD
KPNHKVLFWTDIDQPRAIALDPMMGLMFWTDWGEIPKIEKAGMNGDPSTRKIIVSEEIHWPNGLSL
DYEAKCIYWVDGKLHFLDKIDYNGENRKRVRSQDLLYPYALTRYDSKFFWTDWQNSSIYTLENSTG
ELKKLLHSEKVPCDIRVWESKVQPNNHSHPCEENNGNCSHLCLLSPNPPGYTCACPIGVKLSNNLT
CADGPQEILILARRADICVIYLDSPDYTYRHLNLTNLNYTIGVDYDPVEDFIYWTDDEIMKIQRAK
LKGTQQTDIITHEIQHPDGIAIDWVARNLYWTDPGTDKIQVASLDGKYRNIIINEDLFEPRAIAVA
PELGWLFWSDWNDKVPKIERANLDGTERTIIVSSNLTWPNGIALDLEKEKIYWCDAKTHTIEYANM
DGSERIVLLTDLKHPFGFTLMGDYLYWTDWTRRTVERVHKETGILRETILDQISDVMGLKAVKVGQ
IQGVNPCSLNNGGCSYFCLYRHDKTYVCSCPIEYDLDKDGRTCFIPEAYLFYTSNTSVGRVGIGNG
HVVTLPVRGIKSASSIDFDYINERIYWSDSKHKTIMRAYINGSDPQRIVELGLSAPEGVAIDWAAL
NIYWADPVANKIEVARLSGSSRRTLLWRNIEEPHSIALDPIKGYMYWSQWGQSNCIKKASMDGENP
KTLIMNIKYATGLTLDYEMMRLYWIETATYSIVSTNYDGLNKTVIVSQLDNRPIGLSLYKDLLFWG
NTIKDEVWRVNKRNRENAVKIAGLSEGVTDLSIFHASKQKGLNQCASNNGGCSHLCLALPSQNVEE
SDSYTCACPTHYTLQNNTCLYSSDCPEAVLSIQGLKAVKYIDFDPVQQYLYWIEARTQSIKRAEVT
GAHMAVFVAGGKNVRPFDLAVDALGRLLFWTCEASDVINVTRLENSSVMGVVLPKKDDKPRLIAIH
PTKRLLFYTDVQPTGTQLIRIRMDGTHRISIKKAANITAIAVDVENDLLVWVQGQNITMSNIDGDD

QHVLLSENKSRVTILVVHSGWLYWLDREINQLQRIELKTGQSRSPVLNHASHIVDLVSVIPTDRDH
SCSQITIKKCSHLCIINGTSAVCACPDGLKLQTDRKSCAPLPNCGDDYFSCSVQSQSNKDCIPLAW
RCDKQVDCHDGSDERDCPLCQSDQFRCKSSHCIEGNQLCDKVPQCPDGSDEETCCKEEEFRCPKTG
ACVEVSTLCDGVDDCADGADERKSVCQEANRYTSSQKSGSGIITIVILTTASVVIAFVALFYLLRR
KCNVIETPHEQTEDLLNPLRAQAQLKAQKFRKGMPDVIGMSMLNGSQTSSYDRDHITGASSSTNGS
SIGSYPRETLNPPPSPATTAASTRGSTPSSRYRPYRHYRSINQPPPPTPCSTDVCDESDYNYPTRS
RYDGGPFPPPPTPRSHCHSESCPPSPSSRSSTYFSPLPPPPSPLLGYAWLGSVFFQTGAELTEMSS
WAARMHRRAATFGNVVTSCGHPSGPYPRRLEKVKFGVPLEEVCKNDIPGPLLVLILKLNKEAPFRK
DVFRAPGHQGAMKKLTHFLQTGRLINMDNFSVYTIASVLKKFLRKIPGGVFGRDIEHQFFQIVEMT
DVKAQRDEIHRIIASLPVYTQRLLVLLFGTFRVIASNSETAATGMTSEALGVSVAPSFFHTCVSDG
KTAKMEDVMRFKVATRVMKHLIEEFASSDLFGRDNYEFYARVTGRVLRVQGEWICSFQYPPPQSKG
HSAQIYPGDYSALEQYLLGQVSLEAEKTWLQCECDRWRSKYSLEAMSQVEECQSTPALAEAGLKNQ
VSNSNSLGMIAEHTLLESCTRLSISLEQNGLFKGNGTSRSSSASHSSKTSQTHSGPRMTLEELRAV
NRYAESTRSLSYLPQVHERQTERMRTRSQWFLAPSVECTSCGGSLDIPLNDADVSALTSALLRRSS
SGTIVGAVGLSLSAESIQKRPSLRRSNSKDKRHYLHRSTSRRNKENGSRSNSFKGRSERKNSGSRS
GSFKLKYENISRSCSFKNKNELCTCPSNDKMEEEIDIKTPTVKTLETIHVTLTYKPRI
>hom15424.t1_1
MVQGDCPEGEFQCKLTRRCIFHGWWCDGDPDCGTSAELGPDMSDEVNCKMDTFWCPWNQGRCGNST
VCRPLKVFCDGHNDCPDNSDEFTEFCNNKSRCDNFGCSHECSMTQDGPRCYCPEGRTAPGRTCVDV
DECVLEDSCAQNCVNTVGSFECSCVSGYKAIGHDCHAINGKDFLLPKKCINYLSQNQLKMIYVHQT
PLVVWCAFHTVLGVMNSPFIISVPESEEPSLLFSTEHTIERIDLKGKLWSNKTKTTLMNITALNFF
HRNRTLYYLQHKITNSSLMAVNVDNFSQTWELGMSHLFKNLQSVKQIAFDWVSENWYLLDVQQEVI
ILCTTHLDKCIILLESKMSTPRDLALDPTSGYFFFTKFSERFPSMIERCRMDGTERVTLVDSNIVF
PCSITLDFATKKVYWVDNWLDGVGYVDYDGKNRRIIKEDPLYGSLYAITFLESKWFVSNNKVMVNK
IKVVDKFTRKSNILIDNTTMPLHLHVFHRQRQPDVAHPCKNNNGNCEHFCIPNWNRDIAIAKCMCL
AGYKLQERGRCIQQRPSTFLLMAKMKSALIKGIDLENGTEAIAPITGLSQLSGIEVHVKNRTIFYW
NGTVIEGYNLDTHIRTKMVESGMSLMHAGVAYDWTLGNLYWTQSDSGTISVVKLSDPTLHRTLYFK
RASHPTTIVLNPKRGLMYWIDRPPARLTDGKIRSAWMDGTNVEIFIDTDIRWPSGLSLDLVGKRLY
WSDADLGKIESASLDNKNRKVIFTNLIYPCEVVYFNRSIYYTEYQKGTIISKNLDTNVTRTLVEDA
PISQLRLFNSKSQTGADSYLNDTHCVHLSLKIPNRVVCKCGDGYNFVDSTCVRQKNFSLCREDEFQ
CATYVRCIPKKLVCDGDRNCRDGSDELTADGGPCLRNCSSSEFTCASDGTCVMKHWLCDGEKDCAD
GSDEEPKNCPGLCLPMQFLCAKTKRCIPYAWKCDGDSDCGLLDDSDEGEDCKVKKCDITEFTCHNN
RCIPTDLYCDGADDCHDGSDEIDCAICDPTLHFFCEPSKSCLPNTLRCDNHTDCADGSDERDCKSL
CEKSEFECSNHICVPAVFRCDTFQDCLDGSDERNCNYTKDHPLVSSTEKSSQLESNCEHPSRLCDN
NTRCVPLEHLCDSKYDCLDESDEGLRCLEDLCSLGSICSHLCHNAPEGVVCSCPDDLHLQPDMTTC
LHVHPCESWGVCSQLCTRHGSRHKCDCLPGYVIEKDGFTCKSENASTPYVIFSNRHELRGVDLYSF
NVKALISSLKNTIALDFYHDANSDMIFWTDVIDDKIYRGTLVGNYLSNIEVVVHSGLSTAEGLAVD
WIGENLYWVESNLDQIEVARLNGSFRRTLVGGEMDSPRAIALDPRDGLLFWTDWDNSAPRIERCSM
AGLDRQIIVRVDQITDGAWPNGITLDYDMRRIYWIDARSDSIHTTKYDGTDLHEVMKHHDMLSHPF
AISLFENYVYWTDWRTNSVVRANKWNGGDVFVIQRTLTQPFDIQILHPSRQPRDGLSPCGNNNGGC
SHLCLLHTNHTYRCDCPHVMRLHTDNQTCIVNERVLLIARNNEIRGVDLLQPYYHTIPTISFQNAM
NNRHLEYLAENSTLYWIDSQQNEVKRTGLTSGPAHTLIDTGLQHPTGLALDWLAGLIFIGSSKGIT
VCNLDGEYAMTLIEHISVGSIAVDPTLGRLYWVMENNDNITIDSSAMDGSKHITLVEDLDRTTSGL
TVDRESQRLYWISDFNVYFYDFNAENVTKLNLSGAVSAATVYRGLLYYADDDTQSIHSTNKTTGED
SKILRNSTGVLTLRIYDREEQIGIGPCGKTKTGCQHLCLPISATERTCRCATGYQVDPQDPTKCIG
LSEFLFYSNGWELCGRSLDDSNSTALGPLSRVALASAIDFVAEEDLLFWADNDRGTITSIKRDGTG
RKLIIDQHDGMESVSADWLTGLAIDWSARNIYWSDPKHGVIQVAKLNGSSRYVVLSHEIGTPSALA
VDPEVGLLVWGGASKLETSGLDGSNRKLLVDQARSISDLTLDYVNHYIYWCDSGSDTIERMKYDGS
ERLTLLNHSLENPIALTVFNDVLYWIDSTHQHGSIKLAPVSNLSDYTVLINNTGDSPKDIQIFSKR
RQRGVNPCAQNNGGCEQLCLFNGNSPVCACLHGKVSENGKSCEEQGSVEGVSFEQLHQALYWTCNN
DATISRVNLTKDGKNASNVEVVVRLRSQDKPRGIAIDSCGSYLYWTNWNSQQPSIERVFLSGYGRE
NIISTEIRMPNAITLDHKAQKLYWSDARLDKIERCEYDGKDRQVLGGVTPHHPFALAVYGDYLYWT
DWVLHAVLRADKLTGSNVVSLRRDVAKPMGIIAVANDTDDCFSNPCLELNGGCEDTCNLSPMGEVR
CSCDENRILGEDARRCFGRTTQTCNEDSFRCSDGGCVPLQVTCDKIPHCTDASDEEAGYCAHRVCP
TGWFSCTNRRCLKSKDHCNGVDDCGDASDEANCACSEQNHFKCNSGRCILKQFRCDKDPDCEDASD
EIGCGERNCAEENKDMNFAQCPNTVACIHINWWCDGQNDCWDNADESNCTICQQDVQFKCTGNSKC
IDAEKQCDGIDDCGDGHGRLSSDEQSCHGNCTKDQFKCLSDLLCIESVRKCDGIENCQDGSDEHGC
SVECPYDRFRCHSGECIPQAWQCDGQGDCRDSSDESAHCMTRYCLSSEIRCNSTGGCITPHQLCDG
EDDCADGEDERPTGGCLGKRCSTQEFQCDDGRCINMEYYCDGDDECDDGSDEPEGCHERCEFKCKT
FEHCILKEQVCNGVADCIDGSDEGRHCNNEEEQCIKEGLFKCSNGVCINETLVCNRENDCGDFSDE

ISCNINECTATIKPCSQKCIDKPIGYQCACEIGYELDPNDAQNCVDVDECAEYPCSQICQNTMGSY
HCMCGSNYFLREDKKSCAADSHDIPKLIVANRYYIRELTLHGNATLLIHKLTNVVALDFDWKTQCY
FWSEVTGGKSNIAKQCNLTDDNANITILHSDTLQNPDGLAGLDTIEVSTLGGQNRRVLISTGLEEP
RGIAVHPLKGYLFWSDWGSNVHIGKAGMDGSNPHVIINKVLGWPNAIAIEFETNELFFADAKEDYI
GVSDLDGRNVRIIASRLKYPDLKLHHVFSITLWEDYVYWTDWEMKSVERVHKNHGNMTSSLLTTIH
RPMDLRMVHPFRQPQRDNPCEKANCSALCLLTPEAPFYKCACPENYILLPDNRTCKSNCTSMHFEC
PHSYKCIPFWWKCDGQDDCFDGSDEPADCRPFKCNPGQYQCDDGNCIHPSNICDGTSDCPDKSDEK
NCNDYACLVTHFRCKGNATTPPRCIPGNLRCDNKIDCPFGGEDEEGCPTASCPPQHFQCDNKKCIP
SVWVCDKDNDCGDKSDEFETCTTRTCQRGSFTCKSGRCIPLTWVCDGDGEDEPITCNQPGFHTCDP
SYFRCNNSKCIPGRWYCDYDDDCGDNSDEFNCEPRECSESEFKCKDGRCIQKIHVCDGEYHCKDKS
DEMNCSATLCEADQFKCNDSSNCIPVTWRCDNDLDCIDNSDELSCTESMSCQGTGQFHCDNGRCVD
GRWRCDGEDDCGDNSDEKISQCKRLKCPRGLHKCIENHRCVLRATLCDGHCGELGTEDAIYCRNVT
HCNDKNQFRCTNEHCIDRNLVCDNKNDCEDNSDEVNCICKWNTCSHICLLDKKGAPKCKCSLGFKH
NATTNSCEAHEQNATLILAIENELRLISPYQTNSEKALLGNTELGMGVGYKIGAVDVFYENQDIII
FWTGYRHKQLEKMLLRLNNSSRTSRDISSRTILVDDLIEPYDLAVDWVARRIYLTGTDKVLVTTFD
GKQKYTLINDGTRQRGDIVVAPTQGLLFWVDLGSPAVIETAYMDGNKRRVLVNDVIWPTGLAIDHP
AQRLYWVDQKMHRVESVKFDGSDRQIVHIFDDGKKPHKIDVFEDYLYISTSDTHDVLKMHKFGNGK
IHYLSEGLLTLHDIFVVHEHRQEHGIKNRCQDFCHSSEFCLLSSGGATCFCADGLKKNNLTCSAIM
ECPLNCNQGKCEWIEGIPTCTCPPEYSGTLCEHYRCSLYCHNGGICYVDKDAVGWPLRCHCPAQWT
GEKCEITVNMCEGPCYNGGTCYRYHRTGLGHRCKCKDGFTGVRCENCGNHLKCNNGGVCTHEDNHE
YCKCPLGYGGSNCERFECIEDVCNKRGTCYISSAGTTCRCNSGYYGEHCENESCVPSCQNGATCVM
GARQAECKCPPTFGGQSCEHDLCMNPTPPKECAANGRCYCRNNGTCRTMAGTTVCDCPHMWGGEIC
EKYIGENSACNIYCLNGGDCTLTINNEPTCVCKDGYSGKRCEIFRACPLDCQNGGSCAISDTGPYC
QCTHEYEGPNCEHSSLDESDVRHRMDKEDGGVVLPVLLAFGVILLVVGCIGIAYLILKKRRPFSHE
RLQENDFNNPMYQDRDAEPFSLDADKSTNFVNPVYETVYNGAGSAKDEKTGLLQSNPDEIPPVRNE
ET
>hom15717.t1_1
MKKLCWTNNTDETIQCIEYDGFETKNKAVVLTGVFSTEGLACDWFTNKLYWTESNLHHIEVVSMVD
KPNHKVLFWTDIDQPRAIALDPMMGLMFWTDWGEIPKIEKAGMNGDPSTRKIIVSEEIHWPNGLSL
DYEAKCIYWVDGKLHFLDKIDYNGENRKRVRSQDLLYPYALTRYDSKFFWTDWQNSSIYTLENSTG
ELKKLLHSEKVPCDIRVWESKVQPNNHSHPCEENNGNCSHLCLLSPNPPGYTCACPIGVKLSNNLT
CADGPQEILILARRADICVIYLDSPDYTYRHLNLTNLNYTIGVDYDPVEDFIYWTDDEIMKIQRAK
LKGTQQTDIITHEIQHPDGIAIDWVARNLYWTDPGTDKIQVASLDGKYRNIIINEDLFEPRAIAVA
PELGWLFWSDWNDKVPKIERANLDGTERTIIVSSNLTWPNGIALDLEKEKIYWCDAKTHTIEYANM
DGSERIVLLTDLKHPFGFTLMGDYLYWTDWTRRTVERVHKETGILRETILDQISDVMGLKAVKVGQ
IQGVNPCSLNNGGCSYFCLYRHDKTYVCSCPIEYDLDKDGRTCFIPEAYLFYTSNTSVGRVGIGNG
HVVTLPVRGIKSASSIDFDYINERIYWSDSKHKTIMRAYINGSDPQRIVELGLSAPEGVAIDWAAL
NIYWADPVANKIEVARLSGSSRRTLLWRNIEEPHSIALDPIKGYMYWSQWGQSNCIKKASMDGENP
KTLIMNIKYATGLTLDYEMMRLYWIETATYSIVSTNYDGLNKTVIVSQLDNRPIGLSLYKDLLFWG
NTIKDEVWRVNKRNRENAVKIAGLSEGVTDLSIFHASKQKGLNQCASNNGGCSHLCLALPSQNVEE
SDSYTCACPTHYTLQNNTCLYSSDCPEAVLSIQGLKAVKYIDFDPVQQYLYWIEARTQSIKRAEVT
GAHMAVFVAGGKNVRPFDLAVDALGRLLFWTCEASDVINVTRLENSSVMGVVLPKKDDKPRLIAIH
PTKRLLFYTDVQPTGTQLIRIRMDGTHRISIKKAANITAIAVDVENDLLVWVQGQNITMSNIDGDD
QHVLLSENKSRVTILVVHSGWLYWLDREINQLQRIELKTGQSRSPVLNHASHIVDLVSVIPTDRDH
SCSQITIKKCSHLCIINGTSAVCACPDGLKLQTDRKSCAPLPNCGDDYFSCSVQSQSNKDCIPLAW
RCDKQVDCHDGSDERDCPLCQSDQFRCKSSHCIEGNQLCDKVPQCPDGSDEETCCKEEEFRCPKTG
ACVEVSTLCDGVDDCADGADERKSVCQEANRYTSSQKSGSGIITIVILTTASVVIAFVALFYLLRR
KCNVIETPHEQTEDLLNPLRAQAQLKAQKFRKGMPDVIGMSMLNGSQTSSYDRDHITGASSSTNGS
SIGSYPRETLNPPPSPATTAASTRGSTPSSRYRPYRHYRSINQPPPPTPCSTDVCDESDYNYPTRS
RYDGGPFPPPPTPRSHCHSESCPPSPSSRSSTYFSPLPPPPSPLLGYAWLGSVFFQTGAELTEMSS
WAARMHRRAATFGNVVTSCGHPSGPYPRRLEKVKFGVPLEEVCKNDIPGPLLVLILKLNKEAPFRK
DVFRAPGHQGAMKKLTHFLQTGRLINMDNFSVYTIASVLKKFLRKIPGGVFGRDIEHQFFQIVEMT
DVKAQRDEIHRIIASLPVYTQRLLVLLFGTFRVIASNSETAATGMTSEALGVSVAPSFFHTCVSDG
KTAKMEDVMRFKVATRVMKHLIEEFASSDLFGRDNYEFYARVTGRVLRVQGEWICSFQYPPPQSKG
HSAQIYPGDYSALEQYLLAMSQVEECQSTPALAEAGLKNQVSNSNSLGMIAEHTLLESCTRLSISL
EQNGLFKGNGTSRSSSASHSSKTSQTHSGPRMTLEELRAVNRYAESTRSLSYLPQVHERQTERMRT
RSQWFLAPSVECTSCGGSLDIPLNDADVSALTSALLRRSSSGTIVGAVGLSLSAESIQKRPSLRRS
NSKDKRHYLHRSTSRRNKENGSRSNSFKGRSERKNSGSRSGSFKLKYENISRSCSFKNKNELCTCP
SNDKMEEEIDIKTPTVKTLETIHVTLTYKPRI

Vitellogenin receptor

>abi6250.t1_1
MKPFPYEPDVNITKCQPDDFHCGSGTGPYSEQCIPKEKRCNGYLDCRSGKDEQGCPNKQIACNLDQ
FRCANGQHCIELSQKCDYKSDCDDNSDEQGCTLHCDGYLDCADGSDEINCTAITCQENKFMCPRGA
GGKPKCIDKSLLCNGKRDCEDGSDEETACSKGSCPALGCEYKCQASYTGGMCFCPDTRKLGPDNKT
CIDKDECMEWGYCDQLCINRDGGYTCSCAPGYILRNNRCIVANQGYLNLFFAHDKAIYTMNMTGGN
VKVFANSTSASGIDFHYRKNLLFWSDIKTKRIHVQQLRSTNFFLSSAQNVEIVSPGSWLPVAIAVD
WIGDKLYVADSVGQKIDVFELTGKSHAIVLASNLTYPSDIALDPLVGYFFVADGAQILRANMDGTN
TRSIISEAAYKAYGITTDIIAKRIYWCDALLDYIETANYDGQNRILVIRGPAVPKPARLAHYENRI
YWTDGTKQGIVSVDKYNSSVKNIFHMQDVKDPKAIKIVHTLSQPMTNSPCGNNNGNCQHLCIVTKT
SSGLGFRCACAIGYRLKSDERNCDLVYEFLMYSQQRFIKGKVLNPVIEGFSDAILPVVSRKARFVG
LDFDAKDEYIYYSDVLEDVIYRVYRNGSAREIVLISHNEGVEGLAVDWVSKNLYYIDSRKGTLNVL
STRNITYRRTLLNNLKRPRAIVVHPNRGFIFFSEWDRPANISRAHSDGTNLLVFKNVTLGWPNGLA
IDFTTDRLYWCDALLDHVQHSNLDGTDVKTVNSRLIKHPFSIVIFKDFMYVTDWRLDAIIKLHKLN
GTNEEILVREPQTNRLYGVKPCWYNNGGCQKLCFAIPRNNSNGLSVKCGCPYGEKLREDQKTCQQN
EHNEPPVQACPNTWDFTCNNNRCIPKTWVCDGENDCLDNSDEEQNCTRATCSNLEFSCKSGRCIPK
VFKCDSENDCGDFSDEIGCVNITCSSTQFQCANGNCIPSAWKCDSESDCADGSDEGDSCAEKTCAY
YQYTCPRTGHCIPKTWVCDGDDDCYDKNDEKDCPPTICLPNQFKCADLRQCVQESYKCDGIPDCND
NSDELGCPSIEPDRCDNEKQFQCQSSGICIPRAWYCDGTPDCDDHSDEPTSCGEISCPGDHYKCNN
SKCVFKASICDGNDDCGDGSDESYVHACGKPLFRCADGEWQCPGVTERCVNITNVCDNKPDCPNGA
DEGSGCDLAECNHQGGLCSNGCKQTPEGPLCMCPKGEVLSKDGFTCEDLNECDPPGLCSQRCTNTK
KSYYCSCTAGYTLEPNKHSCKAFNHSAAFLIISNRHSILVADLKEQGLERVPIIVENVVATTSNMH
TGTIFWSDMKLKKISRLDRGSEPVDIISTGLDLVEGLAYDWISGNIYWVDSKLNAIEVSREDGSNR
IVLVKENITQPRGMCLDPSPNARWLFWTDWGENPRVERMGMDGTNRSTIIDTKIYWPNGLTLDIAT
ERVYFADSKFDFIDFCYYNGSGRQQVLAGSHYLLHPHSLTLFEDTLYWTDRQLNRVLSAHKFKGNN
QTVVSHLISQPLSIHVHHPSLQPKSENPCLNNPCEHLCLLSPSVARGFTCKCKPGFTISPNGKCIE
EENPYLMVLRGSQIIDVSIKPGDKATGYLTAIVGVDHGTQVDYDRKGDTIYWVEGKDEDEDNCTVW
TTPYGGGNKTQFLAAETGFVGAPSIIAFDWLGRNLFVGNRIASSFEVIKVDGKFKQRTIILVNNGN
ETSVAKPRSMCLDPTDGKLYWTDEGGSGVPEKIGKVNMDGSDPVVLTINGNPDAITIDINKKILYY
SNQYPPQIKSMNVDGTNEKTIFSKENSIGRIKALGVLDNILFYLDPQYEKLTRIDLPDGNSSKSIL
ENEPELKTFKIFKKRQVVDHPCQINNGGCEHICIPSEKKSNYCTCGIGYRKDNEQSCVPHKTFAIV
NQLDITRGYSLIDSSEAMLPISGPGHHILHVDVHYKENWIYWVEFNKGTWNGIFRIRPNGTELQQI
IKDGIGSNGIRGLAVDWVAGNLYFTNVFPHENYVEVCWLDGSNRKILVKTTTDTPRELAVNPLKRM
LYWIDYGQHPKIGKAYLDGSGWTQVITSGISNPRDLTVDMLTHDIYWVDTRLDMIQKMSYTGGSRQ
VIRRNLPNPMGIAVFKSDMYWVDRNLETVFKSSKILTPNITEPTKVRTNLKKLRDIVIFDILNQPS
DDTNPCLKFGNGGCEQLCFSFPSEANKGPFKCDCANGKLASDRHKCEYINEYIVFTTRTEIRAINL
DPKLTNLPFNPIGNLTNVVGIDFDYADNKILFTQIRPWARIGWLPATNPSSQNIEPLLDKGVNPEG
IAYDWTQKKIYWTDSSNHSIYAMNLDRTDLVTISRVERPRAIVIDPCNGFLYYTDWGKFGTSGKIF
RTTMAGSLKKVIIEKNLAQPSGLAIDYDEKMLYWTDAVREKIERSDMNGENREILISATIYPFSIT
IYGNYIYWTDLQLRGVYRAEKHTGANMIDMVKRLEDSPRDIQIYSVKRQSCTVNPCHINNGGCAQS
CHPSINGTAECKCDGNSKLVNEGRMCVSKNLSCDSTKFYCANGKCISRMWACDGEDDCGDKSDENN
NYCSFHSCSPNEFRCANGRCIFKSWKCDHENDCKDESDEQDCNYPPCASGEFTCANHRCIPMEQVC
NGVNDCKDNTTSDETHERCPTNTTCPANNLKCDKTNICVEPYWLCDGDNDCGDNSDENPLHCAQRT
CPPNSFRCRSHRCIPATWYCDGDDDCGDAADEPPEYCKSEGRTCFGDLFTCDNGNCIPRMYICDGD
NDCLDNSDEDVRHQCNERRCDDETEFTCEANKAWGRAQCISRKWLCDGDPDCVDGADENATLHNCA
SPQPCSDDLFTCSNGRCINQGWVCDHDNDCGDGSDEYKSCNYKTCSTQEFTCQNFKCIRKQYHCDG
EDDCGDMSDEVGCKKENVTCANPGQFRCNNGQCIDHQLVCNKVADCTDESDESLHCNVDECAKVEI
NQCGHKCVNTPTSFYCECNQGYKLLEDGKACADINECVESQGVCSQYCSNTPGSYYCKCNEEFYER
QSDKHTCKRKDNIEPWLIFTNKYYVRNMSTDARTYAIVHQDLMNVVAIDFDMREQKIYFCDVSAKT
IFRSNVDTNDEKEPIIRHDSHGLEGIAVDWIGRKLYWLDRHSKNLDVAELDGTKRKTLRTGIVDPR
GLVVHPGVGYMYFTSWHLQSYIAKLGMDGSNFTRILTWNDDIAWPNALTIDYFTDRIYFADAHLDY
IASVDLEGKHRHIVLSGSKVPHVFALTVFDDYIFWSDWNLKGIYRANKFNGADFRLLRNTTHRPYD
LHIYHPLRQLNYSNPCGTNNGGCSHLCLIAPPHESSYLNIEGYGEEGSTTYICACPNQFYLAQDGK
TCVANCTSGQWTCGGNDEKCISWFWRCDGEKDCKDGSDEPASCPPRVCRVGTFQCKNGHCTQTTTI
CDGTDDCGDGSDEQNCKTPCPELDFKCKTTGRCILDSWKCDGDADCKDGSDEDPAICHKRACDPET
EFTCRNGRCIQALWKCDFDNDCGDDSDEPAYICRQKNCTTGWQRCPGRTNYRCIPKWLFCDGKDDC
RDGSDELPENCLPCNPDTDFKCNNNRCIPKQWKCDFNDDCGDGSDETDNICKGDFRECSESEFRCK
NKKCISTRWRCDHEDDCGDNTDEMGCDGFACKNGTFQCASGHCIASHFHCDGDKDCRDMSDEMNCP
PKYPGGRYCPEYKYQCNNHLCISQSDICDGTDDCGDNSDESSSLCAHFNCNTLRRYQCANHKCVPR

YQVCDGIDNCGDGSDENDMALCATKNKSCNEFQCANKKCVNKAQVCDLADDCGDSSDELGCHHNTV
CSDHDKGGCEHRCTNLTERSYICECNSGYIISKENRKKCQDVNECTTGIHTCSQTCYNLNGTYSCS
CHAGFKLADSLSGVCRALDTDITLIFSNGPEIRGYSLKKRDEFDVISEEKRIEALDYNPKDKIIFW
ADSYDKTIKRSYMINAVDGQVKMGYAQDLNMKGNSKPTALAVDWIGDNIYWTELDRAGPKPKGRIM
VAKTDGRYRRAVVSGGLTSPTSIVVNPQHGTMFWADVGPLPHIETAWMDGFKRKVLVTDNIRHPTG
LAIDYNMDQLLYWADTKLNTIESVKFDGTNRKVILIGDSLKHPISLDVFESNLYWLTRDTGELIKQ
DKFGRGVPVIIQGDLVNPSGIKVYHKLKYNTTINNPCSSSDCSHLCLLVPSGHRCSCPDASIASHK
KSERICDAASERPRPAPRICNCENGGICKESETSDLICQCPEDVMGKFCEIRLAHSKAAGESNTTA
IIIPIVVILLVLAAATGIWFVLRKRPFGKGTTLGSIASSQSVSFRQGTNVEFGPNTFSGNGAGTVE
PLDVSYSLDPINNKNRDFHNPMYDAVQNNPDAIGNGSSALYEIPVDVTKSKSDTFMEPPSAILAPS
SITHRSSPQVNIRHRELDPASDTGKDTQKLVEEDC
>abi8181.t1_1
MNTDKLCIPESFVCNGEPDCLDGSDETLGCSAKIPCEGFKCKNGHCIPHEWRCDGGNDCQDNSDEQ
DCASYLDPKDCLLDDRRFLCTSNNTCLKLSQVCNDKKECPDGSDESPFCTLNTCKDRGCSHECVKL
PNGPKCICPKGYHTISDKTCVDVNECEIYGVCDQKCRNTPGSYECYCDAKYILQEDKSTCRVAGGE
AMMIFSSKTEIRGYFLQSGLYFPIAKNLKQVFGVAFDGHHLYWTDIFSEHKSIVKALEDGSNRELL
VTFGLGAPEDLAVDWITGNIYFTDSEMQHIGVCNDDGSHCTVLVNEDVHKPRGIVLNPSDGEMFWS
DWGKRPEIARSKMDGTEDKSFVSNNIEWPNGLTVDYPNQRLYWADAKFMTLESIRLDGTDRRVILE
GVVKHPYAIAVFENRLFWSDLTTNSIQSCDKFTGKNHHTLIKDKREYIYGIHIFHSALKTKKDNPC
LMAFCSDLCLLSGSDYSCACPQDKILQADKHTCKLSGKRQLLVAGTKDMLIRIEHQLLGKHEVMAL
PTVAKNIGALAYSSINNTLFISDTDSHSIITMHMVTGHSKQLYQVESTSRIESISYDYFGNNLYWC
DLGMETVNILNLNTMSQRILLHDMLGEIPTSIVVVPEEG
>abi9145.t1_1
MNDGHKQLMVHWVGGGSNVTICVARDMAPPAQKMSPSSVYISDDYGTSYENKTDLFKLADGTYSSV
EKFYNHPKYKSYFVFADVRNKHIFVTADYGQTVKKIKLTFTPNDVTFPELDPHVILILDKDDPEKK
LWYTQDFGETFYVLDEHISAFFWVNDDNNTLALQRSQPSNFSTIMYTSATVLSHPTGFFAKDVVAV
IQRSDYIFFTKKATNDSFDLFVAYKLGTKVKCVFDTELPRKAYQVADVTGNRAFVVISHSETVSHL
YVSENLAGQDGLVHFTLSLENVLAHFPNSSWPDSWLQHVTEDAFTDMYKVEGLSGIYIVSQVLYPS
SFGFLLPQNTVTKITFDHGATWRFIKPPTRDAEGQLISCELSNNCSLHLTQRFNQLYPDTRTVSIV
SSKSAPGIILATGVIGKSLKGHYGVYVSSDAGFTWKQVLRDTHLFAMGDYGGIITAVKYSRKLNET
RTILYSTDEAEKWHETSFHEKNMKLYGLMIEPGENTTIFTLFGSLPQKHQWVIVKLDFLNVFSYNC
SDDDYKYWSPNLNSVKEKLVPCTLGQQVTYRRRVPHSNCYSGKNYDLPVSSAPCDCDSSDYECDFG
FIKMADTGHCIRNRSGEDPYKPPSSCKPGQFYNRTKGYRKISGDVCMDGFEKHFTPDFIPCPFKEE
QEFLLYAQKDRIVRLNPVTKRKEDLPVKDLQNVIAIDFDMKNNCVFWGDIVKDHIGRQCLDGASVY
EILINSNLSSVEGMAYDWISHNLYFVDGLRAMIELIRTDVPHPRHIRKTILKPPNLKKPRGIAVHP
IAGYLFWTDWSSDNPSVSRSNLDGTNVKQLFTKPNVEWPNGIAIDHIAERIYWVDARQDYIGSSDL
EGKKFKKVLEKSEFVSHPFSVAVFKDTMYWDDWKINAIFGGDKDHGIAIETLDTHLLGLMDLKIYA
HSIQEGTNSCANASCQYICVAQPNQKGFLCLCPDGMEPTADGKCLCPGGAAHFPNMTCPQIGHTCS
DNYFTCQDGLCIPRSWQCDNENDCLHGDDEIHCRNETCGPHFFTCGNGKCVPQYWKCDYDQDCPDG
SDEVDCHVENCTQNQFHCKNGKCIPFVWKCDGQNDCRDGSDEEDCTPAEPTQCKVSEFTCSTGGIR
CIPLTWRCDQENDCNDNSDEMGCNNATCSDYQFNCGNPPNKCILKQWVCDGDRDCIDGSDEANCTH
TLTTVAPHYPFSPINTTSCHGWMFKCNNNKCIPNWWKCDLSYDCEDGSDEIGCVSVNNTSSDHSTE
SPWNPTVCGYNKFRCLSGECIDLSWVCDGASDCKDGGDEKHCLTVVHCTKDQFKCRVDGSCIQLDE
VCNGENNCPDGSDEHACATDHNTPDISSPPNCSLGFFLCDVSICNPRAVLCDGVANCRDGSDERNC
TSATKKYYQVLQMGIAERGINESSLLLYWWIAVPEHIKLEFLPSISKVGKNIWQNMTWIEHSEYTF
TNLDPYTLYNMTVYVRTNVTNKEFAPAKYYVEKTGEGVPSEPWNITVHQQNGSHILISWNKPMHPS
GVIELYEICWYPPSPPIKMNLTDDSTAHLLSAYFEPEVRYSFYVVAYNRKYKSRISEIKSILFDGD
TDVGPIRDLTVVGYTDNNVSLSWNYTKMYDGFIVGVEARKPYPQLPPRLSNTTKLTITNLAPGVLY
TFKVRSYKGQFSGPDSTITTSTSGMPLPDVTNLQARVIKESGTAVKLSWDRPKDQRKIAWVYGIYY
GITVDETIQGPKYNTTDLSATISDLGACEIYMFTVGIIGPHGFGPLLMESPVVRTYFNKRAPPKNL
SVSQVPGNHLGMLVQWTASCPSIDAIGYIITVREINTNVTNSVRKPNISSIEISHTFTITYGGVYE
VTICTDEPKAICAPSVIYHAPPILPPFEVHVIPERNGSFAIYWQERIMPPGITSYTYEIFVSEGNK
LNESTANRFEVSKPPFTFNNVSADTYSFAVKLKTQDGYSSGLSEITSVKNSLSSTWTDDISKSSLT
TVLAMVCLLLVILGGAFGFLYIRHRRLQNSFTRFANSHYDTRADAATFDDNGLEEEDSPQIRGFSD
DEPLVIA
>abi13041.t1_1
MPQNATCGKNELPCSNGRCIPSYWHCDNEKDCSDGSDEDPQKCRQKECGADEFTCRSVHGECVPLT
WMCDDTPDCSDGSDEKSCNETCRSDEFTCANGKCIQKRWVCDIDNDCGDNSDEKDCPPVTCSPTSE
FQCSENYCITNKWRCDGEYDCQNGADERDCPKTTFTTFCLSREFDCGDHMTCIHNRWLCDGTKDCP
DGIDESPIHCENITCRADQFQCKDLSCIPGNLYCSGTKECADGSDELDCDTPATKCNPKTQFDCGE

GSCIPIEKVCDGHQDCPAWEDEPRDKCGKNECLDKNGGCSHKCVDTLAGYYCECNHGYKLIDNRTC
KDINECEIPGSCSQKCFNDKGSFKCECEQGYIRDPRDLTKCKASEGHASLLFARRRDIRKISLDHH
EMTSIVNETNSATALDFVFRTGMIFWSDVTDRKIYKAPIDEGNEKTVVISEEITTSDGLAVDWIYN
HIYWTDTGKNTIELANFEGQMRKVLITDELEEPRAIALNPKEGWMFWTDWGAEPKIERAGMDGSHR
HTIVSYDIKWPNGLTLDLVKRRVYWVDAKLSVVSSCDYNGRARRVILSNSDLLRHPFSITTFEDWI
YWTDWDKSAVYKANKFTGKNVDAITATEMIQNPMVVHVYHPYRQPDGENHCQAVNGHCSHLCLPAP
QINAHSPLISCACPDSLRMLPDGLTCVQDAVNVTTPYTVTESDYTVTETENIVTTSEYKRFNFWFG
>abi15563.t1_1
MDGTGRTHVIELGLLGPITLYYEFSLHRIFWSDAGTGNIESTSIEGDDRHGFRSLRSNPVGLASLS
KDIFWTNQRSSKIYWADKNNGGYNRKITLDLPEDIDGMRIISISPRPIPSHPCRQNNGNCTHLCLA
VEKSAQCACPLGMNLKVDNVTCFKPKTCAATEFFCKNSETCISRDMLCNGHKDCLMGEDEVDCDVH
NDCLHNEFQCKNGECIKNKFVCDMHYDCKDKTDEMVCSDDKYKRKCPENHFRCSNDLCIAERFVCD
GVKDCYDGDDELINCVSTTCSTEQFRCTIGSCIPKSWECDHEYDCSDLSDEHSGCGSITCSPEKFT
CNNGRCIDKILQCDKVDDCGDMSDEISCKISDRNGCKLNEFQCRGNKTICIPESALCNGTPECPLH
DDEQGCSNCRPDEFECDNKICLTKDWICDGTDDCGDGSDESLNICAYKNHSLLVPSADVPCPHGFR
CKSGQCINIRLLCNGEENCFDGSDEHGACNTSCQVNSNPCSHKCIKTPSGPMCACETGYKLRGDGQ
TCVDIKECSFEPPICSQLCTEQLGSYTCGCFNGYVLRADKISCKSKGEPMSMVFTVNNEIRQLSKA
NNTLSILYSDDSPSISGLDISIELGYIYFSIEQTSTIHRINLKNFSREYLTNVGKPQKLAVDWITQ
NIYFVDGSDKTIRVCNFHQQLCSKLFSLDSNSQISAITVDAVNKHIFYSVTSWWIFNSPNSVIYKC
NLDGSRLQELLKSTKGYITGLAYDIYRKKIFYVDKHNGQINQINYDGNNQMNLIYNLSKPYGLNFF
EDNLYYLMNGGLMGKCKLFGDERSCEAFRLNTQSGELFSILQSSRQPNVGNVCQNNNCTQMCIPTD
VRPKCLCFEGNYVLEGQECKLERREAHLPDKKPLFKPHVTNQKSSIENSSAGSMVAGVLIPIFILL
GAAVVYLFIRRRNSGKFNISMRFYNPMFGISSNNEAPGTVLQVGEHEYSNPVDFCSQDMNAATNAI
LQKVNPLVNIDG
>abi16248.t1_1
MENNLYHTHLNHNFQINDQKLRHQCDGDVDCEKEDDELECGEIEGARSVKCDEEPDKYARCPKTRK
CISRDWLCDGDDDCGDFTDETHCDQFECANGLCIPKTWVCDNDNDCKDLSDENNCTKTGCTEDEFT
CNDGSCISLSWKCDKEPDCNDASDEVECDIEPYACNNGEFHCGDKKCIKIEFKCDSDNDCGDWSDE
DDCPKIPGNCMLGEFKCNSGKCIPDRWRCDKQQDCESNEDEANCNEMAARTCSADEFTCNNGACVW
KKWICDGTPDCAQGEDEAKCEIVCDESKFACTGSSLNDTRTEFCINKKHICDGQKDCPKGEDEKDC
PTKKECEKGSNCSQLCVVTADGNDGCSCLPGYNLAEDGKSCEDINECLYLTDPVCSQTCNNTVGSF
RCGCMTGYILRPDLRSCKALGAPPTLLFANRMDIRQVSLSNQKYTALLKGLHNAIALDYHYEKKLI
FWSDVSIDVIKKAYINGSGVSDVIKWGLESPGGVALDWIHDLLFWTDSGTQRIEVSTLDGKERVII
AANDLDKPRAIAAHPGEALIFWTDWGPVPKIEKANMDGSNRKSIITESVFWPNGLTLDYTSNRIYW
ADAKHNVIETALYDGSDRRKVISKGLPHPFALTIFEDAIYWTDWHTKSISTANKANGAGFRTIHSN
LHFPMDIHSYHSQRQPSYPNRCGPNKGGCEHMCLPNNKSFTCVCPMGQKLKPDGKTCLKPDNMLIF
ARKKDLRLKHLDDNVMHQHEIVIPVDGVKSVVALTWDSKTDSIFWTDVERDTINSAHWNGSNQQVL
VSTNIISPAGLAYDWITDKIYWTDAGTAHIEVANSDGSMRGLLIWEGLDKPRDIVVDPIGGSMYWS
DWGEKPKIEKASMDGTSRIIILIAKNLKWPNSLAIDHSTGKLYWADGGTKSIEFSNLDGTNRRVLLG
PDLPHPFGLDVFGNNIYWTDWSTSNIETANKLTGFNRTVLGTGIALLMDVRVFHRKRNIIDTACNH
DNGGCSHLCLLNPKGHSCACPVGIKLEPGGKNCTNGPMNSLLLAHRFDIRQISLDVPYIVDVVLPF
PQLKNVMSVDVDRKTGEIYWTDTSEQVIEKGTSDGKHVETVMTHELDMVDAIAIDSTGRKIYWTDG
GRNSVEVAELDGSNRKVLIWSDLDSPRAITLHYHHGLMFWSDWGTHAKIEVAEMDGTNRKAIINEK
LEWPNGLAIDRPSGRLYWNDGKLKTIESSDFDGKDRKVILTDVPHPYGLVVVGNHMYWTDWQTQGL
HRAEKTEGAERTIIRNKLEGLMDVRSVQTENIAENVCGKNNGGCSHLCLRNSESCSCACPTGVKLS
KTDNKTCEYQPNNYLMFATRSALARISLDTEQLWDVTLPVRDIINAIDVDFHWKKQLLFYTDVKKH
KIQSVNMKNFSEVTDVISNISSSNGIAVDWIADNIYWTDTVGGLIEVARLDGSNRKILIRENLMEP
RSIAVFPRKGYLYWTEWGKEPKIERSFLDGSSRKTIIVSDLGFPNGLMIDYKSKRLYWTDARWDRI
ENSDLHGMNRIQLVHSNPTIITTHPFGLALFEDYIYWTDWYQKSILRADKATAKNTGIVRSRLEGA
MGITTVSESRQQGWNPCAVDNGGCTHLCFYKISNYTCACPDILDESCKTEPKQWLSIKNPNGDYDD
TDDDTYSYDPSKTDFEDTLLEESDGGLSVRFYIFTLIPMVCLLLLTIVFLSIAFFYKRSKKKYLYT
TGRSIMTFSNPNYYTSSGEPVTSALNNTDRRPFLWKRLKYDKSQERVYEDKTGNTSPEVVSLIPTV
LTPLSSNCEAITPEMDRSPSITPLHRTDSIQPVV
>abi17397.t1_1
MTSSVNLYSGSCIPSNKRCDLKWDCDDGSDETSDCSAMVQGDCPEGEFQCKLTRRCIFHGWWCDGD
PDCGTSAELGPDMSDEVNCKMDTFWCPWNQGRCGNSTVCRPLKVFCDGHNDCPDNSDEFTEFCNNK
SRCDNFGCSHECSMTQDGPRCYCPEGRTAPGRTCVDVDECVLEDSCAQNCVNTVGSFECSCVSGYK
AIGHDCHAINGKDFLLPKKCINYLSQNQLKMIYVHQTPLVVWCAFHTVLGVMNSPFIISVPESEEP
SLLFSTEHTIERIDLKGKLWSNKTKTTLMNITALNFFHRNRTLYYLQHKITNSSLMAVNVDNFSQT
WELGMSHLFKNLQSVKQIAFDWVSENWYLLDVQQEVIILCTTHLDKCIILLESKMSTPRDLALDPT

SGYFFFTKFSERFPSMIERCRMDGTERVTLVDSNIVFPCSITLDFATKKVYWVDNWLDGVGYVDYD
GKNRRIIKEDPLYGSLYAITFLESKWFVSNNKVMVNKIKVVDKFTRKSNILIDNTTMPLHLHVFHR
QRQPDVAHPCKNNNGNCEHFCIPNWNRDIAIAKCMCLAGYKLQERGRCIQQRPSTFLLMAKMKSAL
IKGIDLENGTEAIAPITGLSQLSGIEVHVKNRTIFYWNGTVIEGYNLDTHIRTKMVESGMSLMHAG
VAYDWTLGNLYWTQSDSGTISVVKLSDPTLHRTLYFKRASHPTTIVLNPKRGLMYWIDRPPARLTD
GKIRSAWMDGTNVEIFIDTDIRWPSGLSLDLVGKRLYWSDADLGKIESASLDNKNRKVIFTNLIYP
CEVVYFNRSIYYTEYQKGTIISKNLDTNVTRTLVEDAPISQLRLFNSKSQTGADSYLNDTHCVHLS
LKIPNRVVCKCGDGYNFVDSTCVRQKNFSLCREDEFQCATYVRCIPKKLVCDGDRNCRDGSDELTA
DGGPCLRNCSSSEFTCASDGTCVMKHWLCDGEKDCADGSDEEPKNCPGLCLPMQFLCAKTKRCIPY
AWKCDGDSDCGLLDDSDEGEDCKVKKCDITEFTCHNNRCIPTDLYCDGADDCHDGSDEIDCAICDP
TLHFFCEPSKSCLPNTLRCDNHTDCADGSDERDCKSLCEKSEFECSNHICVPAVFRCDTFQDCLDG
SDERNCNYTKDHPLVSSTEKSSQLESNCEHPSRLCDNNTRCVPLEHLCDSKYDCLDESDEGLRCLE
DLCSLGSICSHLCHNAPEGVVCSCPDDLHLQPDMTTCLHVHPCESWGVCSQLCTRHGSRHKCDCLP
GYVIEKDGFTCKSENASTPYVIFSNRHELRGVDLYSFNVKALISSLKNTIALDFYHDANSDMIFWT
DVIDDKIYRGTLVGNYLSNIEVVVHSGLSTAEGLAVDWIGENLYWVESNLDQIEVARLNGSFRRTL
VGGEMDSPRAIALDPRDGLLFWTDWDNSAPRIERCSMAGLDRQIIVRVDQITDGAWPNGITLDYDM
RRIYWIDARSDSIHTTKYDGTDLHEVMKHHDMLSHPFAISLFENYVYWTDWRTNSVVRANKWNGGD
VFVIQRTLTQPFDIQILHPSRQPRDGLSPCGNNNGGCSHLCLLHTNHTYRCDCPHVMRLHTDNQTC
IVNERVLLIARNNEIRGVDLLQPYYHTIPTISFQNAMNNRHLEYLAENSTLYWIDSQQNEVKRTGL
TSGPAHTLIDTGLQHPTGLALDWLAGLIFIGSSKGITVCNLDGEYAMTLIEHISVGSIAVDPTLGR
LYWVMENNDNITIDSSAMDGSKHITLVEDLDRTTSGLTVDRESQRLYWISDFNVYFYDFNAENVTK
LNLSGAVSAATVYRGLLYYADDDTQSIHSTNKTTGEDSKILRNSTGVLTLRIYDREEQIGIGPCGK
TKTGCQHLCLPISATERTCRCATGYQVDPQDPTKCIGLSEFLFYSNGWELCGRSLDDSNSTALGPL
SRVALASAIDFVAEEDLLFWADNDRGTITSIKRDGTGRKLIIDQHDGMESVSADWLTGLAIDWSAR
NIYWSDPKHGVIQVAKLNGSSRYVVLSHEIGTPSALAVDPEVGLLVWGGASKLETSGLDGSNRKLL
VDQARSISDLTLDYVNHYIYWCDSGSDTIERMKYDGSERLTLLNHSLENPIALTVFNDVLYWIDST
HQHGSIKLAPVSNLSDYTVLINNTGDSPKDIQIFSKRRQRGVNPCAQNNGGCEQLCLFNGNSPVCA
CLHGKVSENGKSCEEQGSVEGVSFEQLHQALYWTCNNDATISRVNLTKDGKNASNVEVVVRLRSQD
KPRGIAIDSCGSYLYWTNWNSQQPSIERVFLSGYGRENIISTEIRMPNAITLDHKAQKLYWSDARL
DKIERCEYDGKDRQVLGGVTPHHPFALAVYGDYLYWTDWVLHAVLRADKLTGSNVVSLRRDVAKPM
GIIAVANDTDDCFSNPCLELNGGCEDTCNLSPMGEVRCSCDENRILGEDARRCFGRTTQTCNEDSF
RCSDGGCVPLQVTCDKIPHCTDASDEEAGYCAHRVCPTGWFSCTNRRCLKSKDHCNGVDDCGDASD
EANCACSEQNHFKCNSGRCILKQFRCDKDPDCEDASDEIGCGERNCAEENKDMNFAQCPNTVACIH
INWWCDGQNDCWDNADESNCTICQQDVQFKCTGNSKCIDAEKQCDGIDDCGDGHGRLSSDEQSCHG
NCTKDQFKCLSDLLCIESVRKCDGIENCQDGSDEHGCSVECPYDRFRCHSGECIPQAWQCDGQGDC
RDSSDESAHCMTRYCLSSEIRCNSTGGCITPHQLCDGEDDCADGEDERPTGGCLGKRCSTQEFQCD
DGRCINMEYYCDGDDECDDGSDEPEGCHERCEFKCKTFEHCILKEQVCNGVADCIDGSDEGRHCNN
EEEQCIKEGLFKCSNGVCINETLVCNRENDCGDFSDEISCNINECTATIKPCSQKCIDKPIGYQCA
CEIGYELDPNDAQNCVDVDECAEYPCSQICQNTMGSYHCMCGSNYFLREDKKSCAADSHDIPKLIV
ANRYYIRELTLHGNATLLIHKLTNVVALDFDWKTQCYFWSEVTGGKSNIAKQCNLTDDNANITILH
SDTLQNPDGLAGLDTIEVSTLGGQNRRVLISTGLEEPRGIAVHPLKGYLFWSDWGSNVHIGKAGMD
GSNPHVIINKVLGWPNAIAIEFETNELFFADAKEDYIGVSDLDGRNVRIIASRLKYPDLKLHHVFS
ITLWEDYVYWTDWEMKSVERVHKNHGNMTSSLLTTIHRPMDLRMVHPFRQPQRDNPCEKANCSALC
LLTPEAPFYKCACPENYILLPDNRTCKSNCTSMHFECPHSYKCIPFWWKCDGQDDCFDGSDEPADC
RPFKCNPGQYQCDDGNCIHPSNICDGTSDCPDKSDEKNCNDYACLVTHFRCKGNATTPPRCIPGNL
RCDNKIDCPFGGEDEEGCPTASCPPQHFQCDNKKCIPSVWVCDKDNDCGDKSDEFETCTTRTCQRG
SFTCKSGRCIPLTWVCDGDGEDEPITCNQPGFHTCDPSYFRCNNSKCIPGRWYCDYDDDCGDNSDE
FNCEPRECSESEFKCKDGRCIQKIHVCDGEYHCKDKSDEMNCSATLCEADQFKCNDSSNCIPVTWR
CDNDLDCIDNSDELSCTESMSCQGTGQFHCDNGRCVDGRWRCDGEDDCGDNSDEKISQCKRLKCPR
GLHKCIENHRCVLRATLCDGHCGELGTEDAIYCRNVTHCNDKNQFRCTNEHCIDRNLVCDNKNDCE
DNSDEVNCICKWNTCSHICLLDKKGAPKCKCSLGFKHNATTNSCEAHEQNATLILAIENELRLISP
YQTNSEKALLGNTELGMGVGYKIGAVDVFYENQDIIIFWTGYRHKQLEKMLLRLNNSSRTSRDISS
RTILVDDLIEPYDLAVDWVARRIYLTGTDKVLVTTFDGKQKYTLINDGTRQRGDIVVAPTQGLLFW
VDLGSPAVIETAYMDGNKRRVLVNDVIWPTGLAIDHPAQRLYWVDQKMHRVESVKFDGSDRQIVHI
FDDGKKPHKIDVFEDYLYISTSDTHDVLKMHKFGNGKIHYLSEGLLTLHDIFVVHEHRQEHGIKNR
CQDFCHSSEFCLLSSGGATCFCADGLKKNNLTCSAIMECPLNCNQGKCEWIEGIPTCTCPPEYSGT
LCEHYRCSLYCHNGGICYVDKDAVGWPLRCHCPAQWTGEKCEITVNMCEGPCYNGGTCYRYHRTGL
GHRCKCKDGFTGVRCENCGNHLKCNNGGVCTHEDNHEYCKCPLGYGGSNCERFECIEDVCNKRGTC
YISSAGTTCRCNSGYYGEHCENESCVPSCQNGATCVMGARQAECKCPPTFGGQSCEHDLCMNPTPP
KECAANGRCYCRNNGTCRTMAGTTVCDCPHMWGGEICEKYIGENSACNIYCLNGGDCTLTINNEPT

CVCKDGYSGKRCEIFRACPLDCQNGGSCAISDTGPYCQCTHEYEGPNCEHSSLDESDVRHRMDKED
GGVVLPVLLAFGVILLVVGCIGIAYLILKKRRPFSHERLQENDFNNPMYQDRDAEPFSLDADKSTN
FVNPVYETVYNGAGSAKDEKTGLLQSNPDEIPPVRNEET
>abi17706.t1_1
MKKLCWTNNTDETIQCIEYDGFETKNKAVVLTGVFSTEGLACDWFTNKLYWTESNLHHIEVVSMVD
KPNHKVLFWTDIDQPRAIALDPMMGLMFWTDWGEIPKIEKAGMNGDPSTRKIIVSEEIHWPNGLSL
DYEAKCIYWVDGKLHFLDKIDYNGENRKRVRSQDLLYPYALTRYDSKFFWTDWQNSSIYTLENSTG
ELKKLLHSEKVPCDIRVWESKVQPNNHSHPCEENNGNCSHLCLLSPNPPGYTCACPIGVKLSNNLT
CADGPQEILILARRADICVIYLDSPDYTYRHLNLTNLNYTIGVDYDPVEDFIYWTDDEIMKIQRAK
LKGTQQTDIITHEIQHPDGIAIDWVARNLYWTDPGTDKIQVASLDGKYRNIIINEDLFEPRAIAVA
PELGWLFWSDWNDKVPKIERANLDGTERTIIVSSNLTWPNGIALDLEKEKIYWCDAKTHTIEYANM
DGSERIVLLTDLKHPFGFTLMGDYLYWTDWTRRTVERVHKETGILRETILDQISDVMGLKAVKVGQ
IQGVNPCSLNNGGCSYFCLYRHDKTYVCSCPIEYDLDKDGRTCFIPEAYLFYTSNTSVGRVGIGNG
HVVTLPVRGIKSASSIDFDYINERIYWSDSKHKTIMRAYINGSDPQRIVELGLSAPEGVAIDWAAL
NIYWADPVANKIEVARLSGSSRRTLLWRNIEEPHSIALDPIKGYMYWSQWGQSNCIKKASMDGENP
KTLIMNIKYATGLTLDYEMMRLYWIETATYSIVSTNYDGLNKTVIVSQLDNRPIGLSLYKDLLFWG
NTIKDEVWRVNKRNRENAVKIAGLSEGVTDLSIFHASKQKGLNQCASNNGGCSHLCLALPSQNVEE
SDSYTCACPTHYTLQNNTCLYSSDCPEAVLSIQGLKAVKYIDFDPVQQYLYWIEARTQSIKRAEVT
GAHMAVFVAGGKNVRPFDLAVDALGRLLFWTCEASDVINVTRLENSSVMGVVLPKKDDKPRLIAIH
PTKRLLFYTDVQPTGTQLIRIRMDGTHRISIKKAANITAIAVDVENDLLVWVQGQNITMSNIDGDD
QHVLLSENKSRVTILVVHSGWLYWLDREINQLQRIELKTGQSRSPVLNHASHIVDLVSVIPTDRDH
SCSQITIKKCSHLCIINGTSAVCACPDGLKLQTDRKSCAPLPNCGDDYFSCSVQSQSNKDCIPLAW
RCDKQVDCHDGSDERDCPLCQSDQFRCKSSHCIEGNQLCDKVPQCPDGSDEETCCKEEEFRCPKTG
ACVEVSTLCDGVDDCADGADERKSVCQEANRYTSSQKSGSGIITIVILTTASVVIAFVALFYLLRR
KCNVIETPHEQTEDLLNPLRAQAQLKAQKFRKGMPDVIGMSMLNGSQTSSYDRDHITGASSSTNGS
SIGSYPRETLNPPPSPATTAASTRGSTPSSRYRPYRHYRSINQPPPPTPCSTDVCDESDYNYPTRS
RYDGGPFPPPPTPRSHCHSESCPPSPSSRSSTYFSPLPPPPSPLLGYAWLGSVFFQTGAELTEMSS
WAARMHRRAATFGNVVTSCGHPSGPYPRRLEKVKFGVPLEEVCKNDIPGPLLVLILKLNKEAPFRK
DVFRAPGHQGAMKKLTHFLQTGRLINMDNFSVYTIASVLKKFLRKIPGGVFGRDIEHQFFQIVEMT
DVKAQRDEIHRIIASLPVYTQRLLVLLFGTFRVIASNSETAATGMTSEALGVSVAPSFFHTCVSDG
KTAKMEDVMRFKVATRVMKHLIEEFASSDLFGRDNYEFYARVTGRVLRVQGEWICSFQYPPPQSKG
HSAQIYPGDYSALEQYLLGQVSLEAEKTWLQCECDRWRSKYSLEAMSQVEECQSTPALAEAGLKNQ
VSNSNSLGMIAEHTLLESCTRLSISLEQNGLFKGNGTSRSSSASHSSKTSQTHSGPRMTLEELRAV
NRYAESTRSLSYLPQVHERQTERMRTRSQWFLAPSVECTSCGGSLDIPLNDADVSALTSALLRRSS
SGTIVGAVGLSLSAESIQKRPSLRRSNSKDKRHYLHRSTSRRNKENGSRSNSFKGRSERKNSGSRS
GSFKLKYENISRSCSFKNKNELCTCPSNDKMEEEIDIKTPTVKTLETIHVTLTYKPRI
>hom5564.t1_1
MKPFPYEPDVNITKCQPDDFHCGSGTGPYSEQCIPKEKRCNGYLDCRSGKDEQGCPNKQIACNLDQ
FRCANGQHCIELSQKCDYKSDCDDNSDEQGCTLHCDGYLDCADGSDEINCTAITCQENKFMCPRGA
GGKPKCIDKSLLCNGKRDCEDGSDEETACSKGSCPALGCEYKCQASYTGGMCFCPDTRKLGPDNKT
CIDKDECMEWGYCDQLCINRDGGYTCSCAPGYILRNNRCIVANQGYLNLFFAHDKAIYTMNMTGGN
VKVFANSTSASGIDFHYRKNLLFWSDIKTKRIHVQQLRSTNFFLSSAQNVEIVSPGSWLPVAIAVD
WIGDKLYVADSVGQKIDVFELTGKSHAIVLASNLTYPSDIALDPLVGYFFVADGAQILRANMDGTN
TRSIISEAAYKAYGITTDIIAKRIYWCDALLDYIETANYDGQNRILVIRGPAVPKPARLAHYENRI
YWTDGTKQGIVSVDKYNSSVKNIFHMQDVKDPKAIKIVHTLSQPMTNSPCGNNNGNCQHLCIVTKT
SSGLGFRCACAIGYRLKSDERNCDLVYEFLMYSQQRFIKGKVLNPVIEGFSDAILPVVSRKARFVG
LDFDAKDEYIYYSDVLEDVIYRVYRNGSAREIVLISHNEGVEGLAVDWVSKNLYYIDSRKGTLNVL
STRNITYRRTLLNNLKRPRAIVVHPNRGFIFFSEWDRPANISRAHSDGTNLLVFKNVTLGWPNGLA
IDFTTDRLYWCDALLDHVQHSNLDGTDVKTVNSRLIKHPFSIVIFKDFMYVTDWRLDAIIKLHKLN
GTNEEILVREPQTNRLYGVKPCWYNNGGCQKLCFAIPRNNSNGLSVKCGCPYGEKLREDQKTCQQN
EHNEPPVQACPNTWDFTCNNNRCIPKTWVCDGENDCLDNSDEEQNCTRATCSNLEFSCKSGRCIPK
VFKCDSENDCGDFSDEIGCVNITCSSTQFQCANGNCIPSAWKCDSESDCADGSDEGDSCAEKTCAY
YQYTCPRTGHCIPKTWVCDGDDDCYDKNDEKDCPPTICLPNQFKCADLRQCVQESYKCDGIPDCND
NSDELGCPSIEPDRCDNEKQFQCQSSGICIPRAWYCDGTPDCDDHSDEPTSCGEISCPGDHYKCNN
SKCVFKASICDGNDDCGDGSDESYVHACGKPLFRCADGEWQCPGVTERCVNITNVCDNKPDCPNGA
DEGSGCDLAECNHQGGLCSNGCKQTPEGPLCMCPKGEVLSKDGFTCEDLNECDPPGLCSQRCTNTK
KSYYCSCTAGYTLEPNKHSCKAFNHSAAFLIISNRHSILVADLKEQGLERVPIIVENVVATTSNMH
TGTIFWSDMKLKKISRLDRGSEPVDIISTGLDLVEGLAYDWISGNIYWVDSKLNAIEVSREDGSNR
IVLVKENITQPRGMCLDPSPNARWLFWTDWGENPRVERMGMDGTNRSTIIDTKIYWPNGLTLDIAT
ERVYFADSKFDFIDFCYYNGSGRQQVLAGSHYLLHPHSLTLFEDTLYWTDRQLNRVLSAHKFKGNN

QTVVSHLISQPLSIHVHHPSLQPKSENPCLNNPCEHLCLLSPSVARGFTCKCKPGFTISPNGKCIE
EENPYLMVLRGSQIIDVSIKPGDKATGYLTAIVGVDHGTQVDYDRKGDTIYWVEGKDEDEDNCTVW
TTPYGGGNKTQFLAAETGFVGAPSIIAFDWLGRNLFVGNRIASSFEVIKVDGKFKQRTIILVNNGN
ETSVAKPRSMCLDPTDGKLYWTDEGGSGVPEKIGKVNMDGSDPVVLTINGNPDAITIDINKKILYY
SNQYPPQIKSMNVDGTNEKTIFSKENSIGRIKALGVLDNILFYLDPQYEKLTRIDLPDGNSSKSIL
ENEPELKTFKIFKKRQVVDHPCQINNGGCEHICIPSEKKSNYCTCGIGYRKDNEQSCVPHKTFAIV
NQLDITRGYSLIDSSEAMLPISGPGHHILHVDVHYKENWIYWVEFNKGTWNGIFRIRPNGTELQQI
IKDGIGSNGIRGLAVDWVAGNLYFTNVFPHENYVEVCWLDGSNRKILVKTTTDTPRELAVNPLKRM
LYWIDYGQHPKIGKAYLDGSGWTQVITSGISNPRDLTVDMLTHDIYWVDTRLDMIQKMSYTGGSRQ
VIRRNLPNPMGIAVFKSDMYWVDRNLETVFKSSKILTPNITEPTKVRTNLKKLRDIVIFDILNQPS
DDTNPCLKFGNGGCEQLCFSFPSEANKGPFKCDCANGKLASDRHKCEYINEYIVFTTRTEIRAINL
DPKLTNLPFNPIGNLTNVVGIDFDYADNKILFTQIRPWARIGWLPATNPSSQNIEPLLDKGVNPEG
IAYDWTQKKIYWTDSSNHSIYAMNLDRTDLVTISRVERPRAIVIDPCNGFLYYTDWGKFGTSGKIF
RTTMAGSLKKVIIEKNLAQPSGLAIDYDEKMLYWTDAVREKIERSDMNGENREILISATIYPFSIT
IYGNYIYWTDLQLRGVYRAEKHTGANMIDMVKRLEDSPRDIQIYSVKRQSCTVNPCHINNGGCAQS
CHPSINGTAECKCDGNSKLVNEGRMCVSKNLSCDSTKFYCANGKCISRMWACDGEDDCGDKSDENN
NYCSFHSCSPNEFRCANGRCIFKSWKCDHENDCKDESDEQDCNYPPCASGEFTCANHRCIPMEQVC
NGVNDCKDNTTSDETHERCPTNTTCPANNLKCDKTNICVEPYWLCDGDNDCGDNSDENPLHCAQRT
CPPNSFRCRSHRCIPATWYCDGDDDCGDAADEPPEYCKSEGRTCFGDLFTCDNGNCIPRMYICDGD
NDCLDNSDEDVRHQCNERRCDDETEFTCEANKAWGRAQCISRKWLCDGDPDCVDGADENATLHNCA
SPQPCSDDLFTCSNGRCINQGWVCDHDNDCGDGSDEYKSCNYKTCSTQEFTCQNFKCIRKQYHCDG
EDDCGDMSDEVGCKKENVTCANPGQFRCNNGQCIDHQLVCNKVADCTDESDESLHCNVDECAKVEI
NQCGHKCVNTPTSFYCECNQGYKLLEDGKACADINECVESQGVCSQYCSNTPGSYYCKCNEEFYER
QSDKHTCKRKDNIEPWLIFTNKYYVRNMSTDARTYAIVHQDLMNVVAIDFDMREQKIYFCDVSAKT
IFRSNVDTNDEKEPIIRHDSHGLEGIAVDWIGRKLYWLDRHSKNLDVAELDGTKRKTLRTGIVDPR
GLVVHPGVGYMYFTSWHLQSYIAKLGMDGSNFTRILTWNDDIAWPNALTIDYFTDRIYFADAHLDY
IASVDLEGKHRHIVLSGSKVPHVFALTVFDDYIFWSDWNLKGIYRANKFNGADFRLLRNTTHRPYD
LHIYHPLRQLNYSNPCGTNNGGCSHLCLIAPPHESSYLNIEGYGEEGSTTYICACPNQFYLAQDGK
TCVANCTSGQWTCGGNDEKCISWFWRCDGEKDCKDGSDEPASCPPRVCRVGTFQCKNGHCTQTTTI
CDGTDDCGDGSDEQNCKTPCPELDFKCKTTGRCILDSWKCDGDADCKDGSDEDPAICHKRACDPET
EFTCRNGRCIQALWKCDFDNDCGDDSDEPAYICRQKNCTTGWQRCPGRTNYRCIPKWLFCDGKDDC
RDGSDELPENCLPCNPDTDFKCNNNRCIPKQWKCDFNDDCGDGSDETDNICKGDFRECSESEFRCK
NKKCISTRWRCDHEDDCGDNTDEMGCDGFACKNGTFQCASGHCIASHFHCDGDKDCRDMSDEMNCP
PKYPGGRYCPEYKYQCNNHLCISQSDICDGTDDCGDNSDESSSLCAHFNCNTLRRYQCANHKCVPR
YQVCDGIDNCGDGSDENDMALCATKNKSCNEFQCANKKCVNKAQVCDLADDCGDSSDELGCHHNTV
CSDHDKGGCEHRCTNLTERSYICECNSGYIISKENRKKCQDVNECTTGIHTCSQTCYNLNGTYSCS
CHAGFKLADSLSGVCRALDTDITLIFSNGPEIRGYSLKKRDEFDVISEEKRIEALDYNPKDKIIFW
ADSYDKTIKRSYMINAVDGQVKMGYAQDLNMKGNSKPTALAVDWIGDNIYWTELDRAGPKPKGRIM
VAKTDGRYRRAVVSGGLTSPTSIVVNPQHGTMFWADVGPLPHIETAWMDGFKRKVLVTDNIRHPTG
LAIDYNMDQLLYWADTKLNTIESVKFDGTNRKVILIGDSLKHPISLDVFESNLYWLTRDTGELIKQ
DKFGRGVPVIIQGDLVNPSGIKVYHKLKYNTTINNPCSSSDCSHLCLLVPSGHRCSCPDASIASHK
KSERICDAASERPRPAPRICNCENGGICKESETSDLICQCPEDVMGKFCEIRLAHSKAAGESNTTA
IIIPIVVILLVLAAATGIWFVLRKRPFGKGTTLGSIASSQSVSFRQGTNVEFGPNTFSGNGAGTVE
PLDVSYSLDPINNKNRDFHNPMYDAVQNNPDAIGNGSSALYEIPVDVTKSKSDTFMEPPSAILAPS
SITHRSSPQVNIRHRELDPASDTGKDTQKLVEEDC
>hom7297.t1_1
LCIPESFVCNGEPDCLDGSDETLGCSAKIPCEGFKCKNGHCIPHEWRCDGGNDCQDNSDEQDCASY
LDPKDCLLDDRRFLCTSNNTCLKLSQVCNDKKECPDGSDESPFCTLNTCKDRGCSHECVKLPNGPK
CICPKGYHTISDKTCVDVNECEIYGVCDQKCRNTPGSYECYCDAKYILQEDKSTCRVAGGEAMMIF
SSKTEIRGYFLQSGLYFPIAKNLKQVFGVAFDGHHLYWTDIFSEHKSIVKALEDGSNRELLVTFGL
GAPEDLAVDWITGNIYFTDSEMQHIGVCNDDGSHCTVLVNEDVHKPRGIVLNPSDGEMFWSDWGKR
PEIARSKMDGTEDKSFVSNNIEWPNGLTVDYPNQRLYWADAKFMTLESIRLDGTDRRVILEGVVKH
PYAIAVFENRLFWSDLTTNSIQSCDKFTGKNHHTLIKDKREYIYGIHIFHSALKTKKDNPCLMAFC
SDLCLLSGSDYSCACPQDKILQADKHTCKLSGKRQLLVAGTKDMLIRIEHQLLGKHEVMALPTVAK
NIGALAYSSINNTLFISDTDSHSIITMHMVTGHSKQLYQVESTSRIESISYDYFGNNLYWCDLGME
TVNILNLNTMSQRILLHDMLGEIPTSIVVVPEEGKAIQVSQYFIFTRKATDIQIFSTCNSLAFHSK
PHITLPSRNLFLFHV
>hom14376.t1_1
MENNLYHTHLNHNFQINDQKLRHQFSFFTNFFFFSHSFRCDGDVDCEKEDDELECGEIEGARSVKC
DEEPDKYARCPKTRKCISRDWLCDGDDDCGDFTDETHCDQFECANGLCIPKTWVCDNDNDCKDLSD

ENNCTKTGCTEDEFTCNDGSCISLSWKCDKEPDCNDASDEVECDIEPYACNNGEFHCGDKKCIKIE
FKCDSDNDCGDWSDEDDCPKIPGNCMLGEFKCNSGKCIPDRWRCDKQQDCESNEDEANCNEMAART
CSADEFTCNNGACVWKKWICDGTPDCAQGEDEAKCEIVCDESKFACTGSSLNDTRTEFCINKKHIC
DGQKDCPKGEDEKDCPTKKECEKGSNCSQLCVVTADGNDGCSCLPGYNLAEDGKSCEDINECLYLT
DPVCSQTCNNTVGSFRCGCMTGYILRPDLRSCKALGAPPTLLFANRMDIRQVSLSNQKYTALLKGL
HNAIALDYHYEKKLIFWSDVSIDVIKKAYINGSGVSDVIKWGLESPGGVALDWIHDLLFWTDSGTQ
RIEVSTLDGKERVIIAANDLDKPRAIAAHPGEALIFWTDWGPVPKIEKANMDGSNRKSIITESVFW
PNGLTLDYTSNRIYWADAKHNVIETALYDGSDRRKVISKGLPHPFALTIFEDAIYWTDWHTKSIST
ANKANGAGFRTIHSNLHFPMDIHSYHSQRQPSYPNRCGPNKGGCEHMCLPNNKSFTCVCPMGQKLK
PDGKTCLKPDNMLIFARKKDLRLKHLDDNVMHQHEIVIPVDGVKSVVALTWDSKTDSIFWTDVERD
TINSAHWNGSNQQVLVSTNIISPAGLAYDWITDKIYWTDAGTAHIEVANSDGSMRGLLIWEGLDKP
RDIVVDPIGGSMYWSDWGEKPKIEKASMDGTSRIILIAKNLKWPNSLAIDHSTGKLYWADGGTKSI
EFSNLDGTNRRVLLGPDLPHPFGLDVFGNNIYWTDWSTSNIETANKLTGFNRTVLGTGIALLMDVR
VFHRKRNIIDTACNHDNGGCSHLCLLNPKGHSCACPVGIKLEPGGKNCTNGPMNSLLLAHRFDIRQ
ISLDVPYIVDVVLPFPQLKNVMSVDVDRKTGEIYWTDTSEQVIEKGTSDGKHVETVMTHELDMVDA
IAIDSTGRKIYWTDGGRNSVEVAELDGSNRKVLIWSDLDSPRAITLHYHHGLMFWSDWGTHAKIEV
AEMDGTNRKAIINEKLEWPNGLAIDRPSGRLYWNDGKLKTIESSDFDGKDRKVILTDVPHPYGLVV
VGNHMYWTDWQTQGLHRAEKTEGAERTIIRNKLEGLMDVRSVQTENIAENVCGKNNGGCSHLCLRN
SESCSCACPTGVKLSKTDNKTCEYQPNNYLMFATRSALARISLDTEQLWDVTLPVRDIINAIDVDF
HWKKQLLFYTDVKKHKIQSVNMKNFSEVTDVISNISSSNGIAVDWIADNIYWTDTVGGLIEVARLD
GSNRKILIRENLMEPRSIAVFPRKGYLYWTEWGKEPKIERSFLDGSSRKTIIVSDLGFPNGLMIDY
KSKRLYWTDARWDRIENSDLHGMNRIQLVHSNPTIITTHPFGLALFEDYIYWTDWYQKSILRADKA
TAKNTGIVRSRLEGAMGITTVSESRQQGWNPCAVDNGGCTHLCFYKISNYTCACPDILDESCKTEP
KQWLSIKNPNGDYDDTDDDTYSYDPSKTDFEDTLLEESDGGLSVRFYIFTLIPMVCLLLLTIVFLS
IAFFYKRSKKKYLYTTGRSIMTFSNPNYYTSSGEPVTSALNNTDRRPFLWKRLKYDKSQERVYEDK
TGNTSPEVVSLIPTVLTPLSSNCEAITPEMDRSPSITPLHRTDSIQPVV
>hom15424.t1_1
MVQGDCPEGEFQCKLTRRCIFHGWWCDGDPDCGTSAELGPDMSDEVNCKMDTFWCPWNQGRCGNST
VCRPLKVFCDGHNDCPDNSDEFTEFCNNKSRCDNFGCSHECSMTQDGPRCYCPEGRTAPGRTCVDV
DECVLEDSCAQNCVNTVGSFECSCVSGYKAIGHDCHAINGKDFLLPKKCINYLSQNQLKMIYVHQT
PLVVWCAFHTVLGVMNSPFIISVPESEEPSLLFSTEHTIERIDLKGKLWSNKTKTTLMNITALNFF
HRNRTLYYLQHKITNSSLMAVNVDNFSQTWELGMSHLFKNLQSVKQIAFDWVSENWYLLDVQQEVI
ILCTTHLDKCIILLESKMSTPRDLALDPTSGYFFFTKFSERFPSMIERCRMDGTERVTLVDSNIVF
PCSITLDFATKKVYWVDNWLDGVGYVDYDGKNRRIIKEDPLYGSLYAITFLESKWFVSNNKVMVNK
IKVVDKFTRKSNILIDNTTMPLHLHVFHRQRQPDVAHPCKNNNGNCEHFCIPNWNRDIAIAKCMCL
AGYKLQERGRCIQQRPSTFLLMAKMKSALIKGIDLENGTEAIAPITGLSQLSGIEVHVKNRTIFYW
NGTVIEGYNLDTHIRTKMVESGMSLMHAGVAYDWTLGNLYWTQSDSGTISVVKLSDPTLHRTLYFK
RASHPTTIVLNPKRGLMYWIDRPPARLTDGKIRSAWMDGTNVEIFIDTDIRWPSGLSLDLVGKRLY
WSDADLGKIESASLDNKNRKVIFTNLIYPCEVVYFNRSIYYTEYQKGTIISKNLDTNVTRTLVEDA
PISQLRLFNSKSQTGADSYLNDTHCVHLSLKIPNRVVCKCGDGYNFVDSTCVRQKNFSLCREDEFQ
CATYVRCIPKKLVCDGDRNCRDGSDELTADGGPCLRNCSSSEFTCASDGTCVMKHWLCDGEKDCAD
GSDEEPKNCPGLCLPMQFLCAKTKRCIPYAWKCDGDSDCGLLDDSDEGEDCKVKKCDITEFTCHNN
RCIPTDLYCDGADDCHDGSDEIDCAICDPTLHFFCEPSKSCLPNTLRCDNHTDCADGSDERDCKSL
CEKSEFECSNHICVPAVFRCDTFQDCLDGSDERNCNYTKDHPLVSSTEKSSQLESNCEHPSRLCDN
NTRCVPLEHLCDSKYDCLDESDEGLRCLEDLCSLGSICSHLCHNAPEGVVCSCPDDLHLQPDMTTC
LHVHPCESWGVCSQLCTRHGSRHKCDCLPGYVIEKDGFTCKSENASTPYVIFSNRHELRGVDLYSF
NVKALISSLKNTIALDFYHDANSDMIFWTDVIDDKIYRGTLVGNYLSNIEVVVHSGLSTAEGLAVD
WIGENLYWVESNLDQIEVARLNGSFRRTLVGGEMDSPRAIALDPRDGLLFWTDWDNSAPRIERCSM
AGLDRQIIVRVDQITDGAWPNGITLDYDMRRIYWIDARSDSIHTTKYDGTDLHEVMKHHDMLSHPF
AISLFENYVYWTDWRTNSVVRANKWNGGDVFVIQRTLTQPFDIQILHPSRQPRDGLSPCGNNNGGC
SHLCLLHTNHTYRCDCPHVMRLHTDNQTCIVNERVLLIARNNEIRGVDLLQPYYHTIPTISFQNAM
NNRHLEYLAENSTLYWIDSQQNEVKRTGLTSGPAHTLIDTGLQHPTGLALDWLAGLIFIGSSKGIT
VCNLDGEYAMTLIEHISVGSIAVDPTLGRLYWVMENNDNITIDSSAMDGSKHITLVEDLDRTTSGL
TVDRESQRLYWISDFNVYFYDFNAENVTKLNLSGAVSAATVYRGLLYYADDDTQSIHSTNKTTGED
SKILRNSTGVLTLRIYDREEQIGIGPCGKTKTGCQHLCLPISATERTCRCATGYQVDPQDPTKCIG
LSEFLFYSNGWELCGRSLDDSNSTALGPLSRVALASAIDFVAEEDLLFWADNDRGTITSIKRDGTG
RKLIIDQHDGMESVSADWLTGLAIDWSARNIYWSDPKHGVIQVAKLNGSSRYVVLSHEIGTPSALA
VDPEVGLLVWGGASKLETSGLDGSNRKLLVDQARSISDLTLDYVNHYIYWCDSGSDTIERMKYDGS
ERLTLLNHSLENPIALTVFNDVLYWIDSTHQHGSIKLAPVSNLSDYTVLINNTGDSPKDIQIFSKR
RQRGVNPCAQNNGGCEQLCLFNGNSPVCACLHGKVSENGKSCEEQGSVEGVSFEQLHQALYWTCNN

DATISRVNLTKDGKNASNVEVVVRLRSQDKPRGIAIDSCGSYLYWTNWNSQQPSIERVFLSGYGRE
NIISTEIRMPNAITLDHKAQKLYWSDARLDKIERCEYDGKDRQVLGGVTPHHPFALAVYGDYLYWT
DWVLHAVLRADKLTGSNVVSLRRDVAKPMGIIAVANDTDDCFSNPCLELNGGCEDTCNLSPMGEVR
CSCDENRILGEDARRCFGRTTQTCNEDSFRCSDGGCVPLQVTCDKIPHCTDASDEEAGYCAHRVCP
TGWFSCTNRRCLKSKDHCNGVDDCGDASDEANCACSEQNHFKCNSGRCILKQFRCDKDPDCEDASD
EIGCGERNCAEENKDMNFAQCPNTVACIHINWWCDGQNDCWDNADESNCTICQQDVQFKCTGNSKC
IDAEKQCDGIDDCGDGHGRLSSDEQSCHGNCTKDQFKCLSDLLCIESVRKCDGIENCQDGSDEHGC
SVECPYDRFRCHSGECIPQAWQCDGQGDCRDSSDESAHCMTRYCLSSEIRCNSTGGCITPHQLCDG
EDDCADGEDERPTGGCLGKRCSTQEFQCDDGRCINMEYYCDGDDECDDGSDEPEGCHERCEFKCKT
FEHCILKEQVCNGVADCIDGSDEGRHCNNEEEQCIKEGLFKCSNGVCINETLVCNRENDCGDFSDE
ISCNINECTATIKPCSQKCIDKPIGYQCACEIGYELDPNDAQNCVDVDECAEYPCSQICQNTMGSY
HCMCGSNYFLREDKKSCAADSHDIPKLIVANRYYIRELTLHGNATLLIHKLTNVVALDFDWKTQCY
FWSEVTGGKSNIAKQCNLTDDNANITILHSDTLQNPDGLAGLDTIEVSTLGGQNRRVLISTGLEEP
RGIAVHPLKGYLFWSDWGSNVHIGKAGMDGSNPHVIINKVLGWPNAIAIEFETNELFFADAKEDYI
GVSDLDGRNVRIIASRLKYPDLKLHHVFSITLWEDYVYWTDWEMKSVERVHKNHGNMTSSLLTTIH
RPMDLRMVHPFRQPQRDNPCEKANCSALCLLTPEAPFYKCACPENYILLPDNRTCKSNCTSMHFEC
PHSYKCIPFWWKCDGQDDCFDGSDEPADCRPFKCNPGQYQCDDGNCIHPSNICDGTSDCPDKSDEK
NCNDYACLVTHFRCKGNATTPPRCIPGNLRCDNKIDCPFGGEDEEGCPTASCPPQHFQCDNKKCIP
SVWVCDKDNDCGDKSDEFETCTTRTCQRGSFTCKSGRCIPLTWVCDGDGEDEPITCNQPGFHTCDP
SYFRCNNSKCIPGRWYCDYDDDCGDNSDEFNCEPRECSESEFKCKDGRCIQKIHVCDGEYHCKDKS
DEMNCSATLCEADQFKCNDSSNCIPVTWRCDNDLDCIDNSDELSCTESMSCQGTGQFHCDNGRCVD
GRWRCDGEDDCGDNSDEKISQCKRLKCPRGLHKCIENHRCVLRATLCDGHCGELGTEDAIYCRNVT
HCNDKNQFRCTNEHCIDRNLVCDNKNDCEDNSDEVNCICKWNTCSHICLLDKKGAPKCKCSLGFKH
NATTNSCEAHEQNATLILAIENELRLISPYQTNSEKALLGNTELGMGVGYKIGAVDVFYENQDIII
FWTGYRHKQLEKMLLRLNNSSRTSRDISSRTILVDDLIEPYDLAVDWVARRIYLTGTDKVLVTTFD
GKQKYTLINDGTRQRGDIVVAPTQGLLFWVDLGSPAVIETAYMDGNKRRVLVNDVIWPTGLAIDHP
AQRLYWVDQKMHRVESVKFDGSDRQIVHIFDDGKKPHKIDVFEDYLYISTSDTHDVLKMHKFGNGK
IHYLSEGLLTLHDIFVVHEHRQEHGIKNRCQDFCHSSEFCLLSSGGATCFCADGLKKNNLTCSAIM
ECPLNCNQGKCEWIEGIPTCTCPPEYSGTLCEHYRCSLYCHNGGICYVDKDAVGWPLRCHCPAQWT
GEKCEITVNMCEGPCYNGGTCYRYHRTGLGHRCKCKDGFTGVRCENCGNHLKCNNGGVCTHEDNHE
YCKCPLGYGGSNCERFECIEDVCNKRGTCYISSAGTTCRCNSGYYGEHCENESCVPSCQNGATCVM
GARQAECKCPPTFGGQSCEHDLCMNPTPPKECAANGRCYCRNNGTCRTMAGTTVCDCPHMWGGEIC
EKYIGENSACNIYCLNGGDCTLTINNEPTCVCKDGYSGKRCEIFRACPLDCQNGGSCAISDTGPYC
QCTHEYEGPNCEHSSLDESDVRHRMDKEDGGVVLPVLLAFGVILLVVGCIGIAYLILKKRRPFSHE
RLQENDFNNPMYQDRDAEPFSLDADKSTNFVNPVYETVYNGAGSAKDEKTGLLQSNPDEIPPVRNE
ET
>hom15717.t1_1
MKKLCWTNNTDETIQCIEYDGFETKNKAVVLTGVFSTEGLACDWFTNKLYWTESNLHHIEVVSMVD
KPNHKVLFWTDIDQPRAIALDPMMGLMFWTDWGEIPKIEKAGMNGDPSTRKIIVSEEIHWPNGLSL
DYEAKCIYWVDGKLHFLDKIDYNGENRKRVRSQDLLYPYALTRYDSKFFWTDWQNSSIYTLENSTG
ELKKLLHSEKVPCDIRVWESKVQPNNHSHPCEENNGNCSHLCLLSPNPPGYTCACPIGVKLSNNLT
CADGPQEILILARRADICVIYLDSPDYTYRHLNLTNLNYTIGVDYDPVEDFIYWTDDEIMKIQRAK
LKGTQQTDIITHEIQHPDGIAIDWVARNLYWTDPGTDKIQVASLDGKYRNIIINEDLFEPRAIAVA
PELGWLFWSDWNDKVPKIERANLDGTERTIIVSSNLTWPNGIALDLEKEKIYWCDAKTHTIEYANM
DGSERIVLLTDLKHPFGFTLMGDYLYWTDWTRRTVERVHKETGILRETILDQISDVMGLKAVKVGQ
IQGVNPCSLNNGGCSYFCLYRHDKTYVCSCPIEYDLDKDGRTCFIPEAYLFYTSNTSVGRVGIGNG
HVVTLPVRGIKSASSIDFDYINERIYWSDSKHKTIMRAYINGSDPQRIVELGLSAPEGVAIDWAAL
NIYWADPVANKIEVARLSGSSRRTLLWRNIEEPHSIALDPIKGYMYWSQWGQSNCIKKASMDGENP
KTLIMNIKYATGLTLDYEMMRLYWIETATYSIVSTNYDGLNKTVIVSQLDNRPIGLSLYKDLLFWG
NTIKDEVWRVNKRNRENAVKIAGLSEGVTDLSIFHASKQKGLNQCASNNGGCSHLCLALPSQNVEE
SDSYTCACPTHYTLQNNTCLYSSDCPEAVLSIQGLKAVKYIDFDPVQQYLYWIEARTQSIKRAEVT
GAHMAVFVAGGKNVRPFDLAVDALGRLLFWTCEASDVINVTRLENSSVMGVVLPKKDDKPRLIAIH
PTKRLLFYTDVQPTGTQLIRIRMDGTHRISIKKAANITAIAVDVENDLLVWVQGQNITMSNIDGDD
QHVLLSENKSRVTILVVHSGWLYWLDREINQLQRIELKTGQSRSPVLNHASHIVDLVSVIPTDRDH
SCSQITIKKCSHLCIINGTSAVCACPDGLKLQTDRKSCAPLPNCGDDYFSCSVQSQSNKDCIPLAW
RCDKQVDCHDGSDERDCPLCQSDQFRCKSSHCIEGNQLCDKVPQCPDGSDEETCCKEEEFRCPKTG
ACVEVSTLCDGVDDCADGADERKSVCQEANRYTSSQKSGSGIITIVILTTASVVIAFVALFYLLRR
KCNVIETPHEQTEDLLNPLRAQAQLKAQKFRKGMPDVIGMSMLNGSQTSSYDRDHITGASSSTNGS
SIGSYPRETLNPPPSPATTAASTRGSTPSSRYRPYRHYRSINQPPPPTPCSTDVCDESDYNYPTRS
RYDGGPFPPPPTPRSHCHSESCPPSPSSRSSTYFSPLPPPPSPLLGYAWLGSVFFQTGAELTEMSS

WAARMHRRAATFGNVVTSCGHPSGPYPRRLEKVKFGVPLEEVCKNDIPGPLLVLILKLNKEAPFRK
DVFRAPGHQGAMKKLTHFLQTGRLINMDNFSVYTIASVLKKFLRKIPGGVFGRDIEHQFFQIVEMT
DVKAQRDEIHRIIASLPVYTQRLLVLLFGTFRVIASNSETAATGMTSEALGVSVAPSFFHTCVSDG
KTAKMEDVMRFKVATRVMKHLIEEFASSDLFGRDNYEFYARVTGRVLRVQGEWICSFQYPPPQSKG
HSAQIYPGDYSALEQYLLAMSQVEECQSTPALAEAGLKNQVSNSNSLGMIAEHTLLESCTRLSISL
EQNGLFKGNGTSRSSSASHSSKTSQTHSGPRMTLEELRAVNRYAESTRSLSYLPQVHERQTERMRT
RSQWFLAPSVECTSCGGSLDIPLNDADVSALTSALLRRSSSGTIVGAVGLSLSAESIQKRPSLRRS
NSKDKRHYLHRSTSRRNKENGSRSNSFKGRSERKNSGSRSGSFKLKYENISRSCSFKNKNELCTCP
SNDKMEEEIDIKTPTVKTLETIHVTLTYKPRI

Juvenile hormone acid o-methyltransferase

>abi7111.t1_1
MHFVNNLKKYVIKIRSRLLAVQVRLFPLEKNPRNDILLSNGVTRNTKVGPKEKKNAMNEAQLYSRH
NGLQKTDSAYVLEKYFHLVKWRNTGEESVLDVGCGDGNVTIELLLPLLGKSFKKLIGVDLSGEMIE
YAKAYNNRDKVHYVQLDVSTKELHVDYKEKFDHIFSFYCLHWIQEQRQCIQNLFKLLKPNGDMLLT
FLATNPIYKVYENLSKYTKWSPYMKNVRKYLSPYHNSENPTKELRTTLIQTGFRVNVCKILKRTYT
FPDFITWKRSIIAVNPFLPKIPEHMREDYIFDFFDEVRKLKFVTPGDDNNNGEKIYTKHKLFVVHA
SRLK
>abi7916.t1_1
MIHNQRQFVENLYKMLKPGGQAFLTSLFRNPFFRSWLALSKCEKWAEHIGHFPEFCSEYFTCDEPV
KLFEKIFKEAGFAIEFITTVPMYEKFFNAEKFERAVTEVNLPVDSIPEALKKEFIKDNIEMMYKLC
NNTYVVENETAVAVVKKPTD
>abi8215.t1_1
XSITPKHASLFLTKYGNLLSWTDKKEIIADIGCANGQITTDILLPFCGDKVEKIIGMDISPEMLVL
ANERNKKSNVSFLEMDVAKKNLDSNMIGKFDRVFSFYCLHMINNQRQFAGNVYKMLKPGGEIFLTC
LFKNDLIESIFMMSKSGKWSTYLENFPNTFSSYFTTAQPQEDLDRVFRDVGFSIDSHKSGPLEWTV
DRGELEAMIKQVNLVVDYIPQSLREEFLNDNVELAKKLCKQRADSFVVNLNAIIAVAKKPVS
>abi10294.t1_1
MHLPTSYCDHFTFPSEHASSFLEKYGHLLTYKNDRKEIIADIGCADGKTTTKILLPYLGEKVNEIV
GIDISSAMLELAKKNNKNSMVSYVEMDAATKNLNPDMLEKFDHIFSFYCLHWIHDQRQCAENLYKM
LKPGGEAFLTFAVRNQHFQCWFDLSQCTKWIEYVGNFPQFCSEYFINDEPIKLFESVFKEAGFAIE
FIDSMKMSFTFTQDKFESAIRDVCVPLNSIPEELREEYIKDNIEMMYKLCNNTCTIEVLTVIAIVK
KPIFYVENV
>abi11257.t1_1
MHLSTEYCNYMIFAPAHAKAFLKNYGPLLTWKTDKEIIADVGCANGEITTEILLPFCEKNVDKIIG
IDISKAMLELANKNSKTSAVSFLELDVANKNLSPDMFEKFDHIFSFYCLHWIHDQRQCAENLYKML
KPGGEAFLTIMFRNSFIQSWFILSRCEKWSKYIGNFPKNCSQYFTHDEPQKLFETVFKDAGFAIKF
IEVVHMNTTYTEEKLKQAIVAVNVPIDLIPEALREDYLKDNNEIFYKLTNDTCEIKRQVIVAIVKK
PSS
>abi12772.t1_1
MATRLTTNFCEFIPITPEHASLFLTKYGNLLSWTGKKEIIADIGCANGQLTTDILLPFCGDKVEKI
IGIDISPEMLVLANERNKKSNVSFLEMDVAKKDLDSNMIGKFDHVFSFYCVHMINNQRQFAENVYK
MLKPGGEIFLTCHYKNDHIESAFMLSKSKKWSTYLENFPNTYSPYFTSAQPQEDLERVFKDVGFSI
ESHKSGPLEWEMDRDEFETMVKEVSLVIDYIPQRLREEFLNDSVELTTKLCKRGDSFVVKNNVIVA
VAKKPVS
>abi14226.t1_1
MITINSGNDTSPEYYHRCCANGAITTDILSPFCKNKFEEIIGIDISKDMIKLACERNKTQNNSFVE
MDIKTKDLNVDMFGKLQHIFSFCCLHMVTDKRQCAQNLFKMLKPDGELFLTFEMRNTIAENRHTMC
ENEKWSKYLEDFRKTYPTPTHPYIRTRHRKCFYGCWIFDIVLHVNIVVNYIPENQKEEFRKEQLGL
MKELCKNGDSYEISFQQLVVIARKPKT
>abi18458.t1_1
MLLLANERNRKSNVSFLEMDVTKKNLDSDVTGKFDRAFSFYCLDLINNQRQYAENVYKMLKPGGEI
FLTCLFTNEIIESAFTMSKSEKWSSYLENAPNNFSSYFTSAQPQEDLERIFKDAGFSIDSHKCGPL
EWTMDRGEFETLVKEVNLVLDYIPPSLREEFLNDNIELVTKLCERADSFVINYNAIIAVAKKPAS
>abi19961.t1_1
MATSLTTNYCKFFSIAPKHASLFLTKFGNLLSWTGKKEIIADIGCANGEITTDILLPFCGDKVEKI
IGIDISSEMLLLANERNKKSNVSFLEMDVAKKNLDSDMIGKFDRVFSFYCVHMINNQRQFAENIYK
MLKPGGEIFLTCLVKCEIIESAFTMSKSEKWSTYLENFPNNFSSYFTSAQPQEDLNRVFKDVGFSI
DSRKSEPLEWTMDRGEFKTVMKEVNLLVGYIPPSLREEFLNDNVELMKKLCKRADSFVVTYDVTIA
VAKKPTS
>abi24292.t1_1
XQFAENVYKMLKPGGEIFLTCNFKNDLIESALMISKSEKWSTYLENFPNTYSPYFTSAQPQEDLDR
VFKDVGFSIDSHKSGPLEWTVDRGELEAMVKEVNFAVDYVPQRLREEFLNDSVELTTKLCKRADSF
VVTINAIVAVAKKPVS
>hom6340.t1_1
MNEAQLYSRHNGLQKTDSAYVLEKYFHLVKWRNTGEESVLDVGCGDGNVTIELLLPLLGKSFKKLI
GVDLSGEMIEYAKAYNNRDKVHYVQLDVSTKELHVDYKEKFDHIFSFYCLHWIQEQRQCIQNLFKL
LKPNGDMLLTFLATNPIYKVYENLSKYTKWSPYMKNVRKYLSPYHNSENPTKELRTTLIQTGFRVN

```
VCKILKRTYTFPDFITWKRSIIAVNPFLPKIPEHMREDYIFDFFDEVRKLKFVTPGDDNNNGEKIY
TKHKLFVVHASRLK
>hom12593.t1_1
MIKLACERNKTQNNSFVEMDIKTKDLNVDMFGKLQHIFSFCCLHMVTDKRQCAQNLFKMLKPDGEL
FLTFEMRNTIAENRHTMCENEKWSKYLEDFRKTYPTPTHPYIRTRHRKCFYGCWIFDIVLHVNIVV
NYIPENQKEEFRKEQLGLMKELCKNGDSYEISFQQLVVIARKPKT
>hom16433.t1_1
MLLLANERNRKSNVSFLEMDVTKKNLDSDVTGKFDRAFSFYCLDLINNQRQYAENVYKMLKPGGEI
FLTCLFTNEIIESAFTMSKSEKWSSYLENAPNNFSSYFTSAQPQEDLERIFKDAGFSIDSHKCGPL
EWTMDRGEFESMWRFFSNEKTNFKLFAALVKEVNLVLDYIPPSLREEFLNDNIELVTKLCERADSF
VINYNAIIAVAKKPAS
```

Malvolio

>abi2268.t1_1
MSGDTPTTPEDVVEPSAIARSNQIPSASEDTLLTPSQTYFTDEKVPIPEIESRKFSFRKLWAFTGP
GFLMSIAYLDPGNIESDLQSGTIAEYRLLWVLFSATVLGLLMQRLAARLGVVTGLHLAEMCYRQYR
TFPRLVLWIMIEIAIIGSDMQEVIGTAIAIYLLSDKTIPLWGGVLITIVDTFTFLFLDKYGLRKLE
LFFGTLITIMGVTFGYEYIVSKPDQVEVVKGMFIPWCKGCDSQALLQAVGIVGAVIMPHNLYLHSA
LVKSRDVDRTDRNKVKDANMYFFIEAAIALFVSFIINVFVVAVFAKGLHHTTNNQILERCSHYPSI
NSSIFPQNDEYVTADLFKGGIFLGCTFGLAAMYIWAVGILAAGQSSTMTX
>abi18260.t1_1
MADVTRPLRLLLKKDTERYWNNQQEPILLSVDASSYACGGVLIQKGKPIAYCAKSFTTTEIGYSQL
EKEANAILVACKKFHSYIWGCKNLTIESDHKPLETIFKKPLTEAPPRLQRILYQILPYNPKIIYKK
GTEMHVADTLSRDCQNLSCEEMHNGTLQICAVVPFSKPRTYAGQFTMEGFLNLQWARWRRVLLTRT
IAIVPTFCLAFFTRIEDLTGMNDSLNAIMSLQLPFATIPTIAFTSNKKIMGEFVNGWANKIIASLL
SVLVIAINTYFVIQTVNDYEFHWVALTLVVVIGIIYLIFCAYLVIHMAVSMGNTSLLRYDFVHKYV
MRPNEMEYVISPANYSR
>abi19119.t1_1
MTVIETKNNTVKKSEEEEFETYFSNERVKIPEDETKGFSFRKLWAFTGPGFLMSIAYLDPGNIESD
LQSGTVAGYKLLWVLLTATALGLVAQTLATKLGVTTGLHLAEMCYRQYKTFPRLVVWVMIEIAVIG
SDMQEVIGTGIAIYLLSNKVIPLWAGCLITIIDTFTFLFLDKYGLRKLELLFGVLIVVMSVTFGYE
YIVAAPDQGSVMAGMFTPWCKDCDSGALLQAVGIVGAVIMPHNLYLHSALVKSRDIDRKKPEKIKE
ANFYYLIESSIALLCSFIINVFVVAVFAKYNLQLEQCAANDIDAIDVFPNDDEYVSADIYKGGIFL
GCMFGATALYIWGVGILAAGQSSTMTGTYAGQFAMEGFLNLRWARWKRVLFTRTIAIIPTFCTAFF
SNIDQLTNLNDILNAVMSLQLPFAIIPTIAFTSNIKIMGQFVNGIIIKIVSIVLAVGVIAINIYFV
YSTLEDLELSAPALAGVYVGAVLYFCLCIYLIIHMTASILVDTLGQKPFIKKYVFNDVHNLDLKI
>hom16241.t1_1
MEGFLNLQWARWRRVLLTRTIAIVPTFCLAFFTRIEDLTGMNDSLNAIMSLQLPFATIPTIAFTSN
KKIMGEFVNGWANKIIASLLSVLVIAINTYFVIQTVNDYEFHWVALTLVVVIGIIYLIFCAYLVIH
MAVSMGNTSLLRYDFVHKYVMRPNEMEYVISPANYSR
>hom17008.t1_1
MTVIETKNNTVKKSEEEEFETYFSNERVKIPEDETKGFSFRKLWAFTGPGFLMSIAYLDPGNIESD
LQSGTVAGYKLLWVLLTATALGLVAQTLATKLGVTTGLHLAEMCYRQYKTFPRLVVWVMIEIAVIG
SDMQEVIGTGIAIYLLSNKVIPLWAGCLITIIDTFTFLFLDKYGLRKLELLFGVLIVVMSVTFGYE
YIVAAPDQGSVMAGMFTPWCKDCDSGALLQAVGIVGAVIMPHNLYLHSALVKSRDIDRKKPEKIKE
ANFYYLIESSIALLCSFIINVFVVAVFAYGLHHTTNNELNDDEYVSADIYKGGIFLGCMFGATALY
IWGVGILAAGQSSTMTGTYAGQFAMEGFLNLRWARWKRVLFTRTIAIIPTFCTAFFSNIDQLTNLN
DILNAVMSLQLPFAIIPTIAFTSNIKIMGQFVNGIIIKIVSIVLAVGVIAINIYFVYSTLEDLELS
APALAGVYVGAVLYFCLCIYLIIHMTASILVDTLGQKPFIKKYVFNDVHNLDLKI

Neuropeptide F

>abi3124.t1_1
MDRGSSRSKKRKKWFIIEKVLPNVLCDVMWLCVVFDAELTKSAVRDIEACSKNAWGVRQHVKIERD
RSTIHMTRTQNPRNLCRNFGTMDFKKLLILIENRDSIRDLYELLMQKEALEDRIGAHQVSRKSSRT
PSLRLRFGRRSDPSLVPDIKLCMSVSFHSKALMVLRRIFWHRKPATIKPSTLQDKNNYQNVCKRYF
R
>hom2776.t1_1
MWLCVVFDAELTKSAVRDIEACSKNAWGVRQHVKIERDRSTIHMTRTQNPRNLCRNFGTMDFKKLL
ILIENRDSIRDLYELLMQKEALEDRIGAHQVSRKSSRTPSLRLRFGRRSDPSLVPASPYLLAQETG
DN

Odorant receptor

>abi498.t1_1
MQYQIKDCALKKKKKTRIDERILVGWYKRLLCSFEGLHLKFNYGHDDSSTLEEIVEGVYVTTCFTN
LLFNSVCLIITGRESYKMFEKLKNFEKFGVPNNVAFVDKMLHYYNMGAFGFTFFGPFFLQLFMMMD
TKRCKAHYAKLGRDRLCGFVIRLAYPYDIKESPRWELHVMAVTFFSTFACSTGAFSTTWSCVAIEF
IILRIEHLKRLLRKVFDEDGYEKRKKRLHRCITYHIHIIE
>abi1173.t1_1
MIDDFESMFSVGMLFQFLSSVSSDVGEACYMSNWYECDKKNLKMVFIIMERAKRPVLLTAGHFFTL
SLVTLVMVVTYRNFEMLSSNSKLLDNAYCLFALEMRILRLIERVTRKEIIENNYLRWFHLVRMPES
GIVKKLWEMGVSGKRYGGRPKITWNESVAKTVSAKRLTRRKTLDRNK
>abi1702.t1_1
MDIENNDFFHVNIVILKWAGIWIPTKNISTKLKAWYIVYNSFWITYSCIIFTPFELLSFQNTASDL
NELIRNVNMGMTHFLALIKISIWLYRRNEILNIIGVLGEYSRIYETFDVFDVDKIIAKERRFKDIA
TKGFLISALSVSVTACLAALKNLFLASVETDSYESNNVTDSFLVSRNLPYHSLIPFNYTTSKLRFV
MAIFYQCFGVFNFGWIIVGLDTLYVAMLSYICAHLQIIQFAFKTIRPRCLKKMGLPHVEVLHDSDF
VNNEMTLEMNKITRHLQIILNICQHVEDIYTYVTLAQVLISLVVYCTCLYMVSTIPITSADFSTHF
IYLLAIQCQLGIYCFFGNKVALSSEGIPSALYKGDWLSANDSFKRSMLITMSRMLIPIHFTMGKFS
PLTLSTFVT
>abi2265.t1_1
MTVNCGIINFFKKRLFDVSEKWAYPYVPITLIDTTYSPVFEIACMYQISCVFTYAGIIGTIDVLLS
VIMAHLSVQLKILQNAFRHVRSRINTMRKNDVVKQPYGHYAHIVLGDNIKHHLQTLELVNEMEDIC
YVMFLVILLASVLQLCFLLYQTSLLPVKSVVFIQNFFYYWIVFLQVGMNCYWGNEVTLEAARMADA
ATDFDWTDLPTYLSKALVLVVARSQKPLYITAGKFVPLSLPAFMSILKGSFSYFMVMRRSQEEEE
>abi2266.t1_1
MASLVKAFKDPKLPENFFRLQTEVKEQKGELKHAEERKGSTTQNRRVDERDEKLITRDNKNDKEMD
QARKMEKRKEHREKKVNNGRTSSNKTENTKQENKLQILETRNNDESGKEQELLYEFNKVGLPDYNR
NLKKGQREIKLEGGLILVYSSVTKEKRAAARVGCTNHKIHKWIVEMVTILRMLGIELVKEESLGYK
IYSRTILFYLIYSYTFVEALEIYSQWGDMNAIIGVCSFLVTHITGVIKTTLLFANRKKIGNMLKTL
HKKPFLPDENRGGEHEEIYVRNVIRSTELMLVVYFAIVSVTLFTGVITFGWTRIFGKKDEWEYPYV
KITIIDTSYSPTFEIACIYQISCVMFAAPIIASIYCLLSTMIAHLSVQMKILQNAFRNTRKQAQGM
QDKNNKFTEIDYMTSLLGEYVEQHLQVFEFAKELEDLCNIMILLIVLASGIMLCFNLYQSSKGGQA
WEKSGSNFRAWPQPRARSSHTEIGSITFIHYSSYYLIIVNQIYMYCHWGDKVTVESANVAQAVAEV
DWVDAPISVKKALILVIARSQRPLRMTAGKFVPLSIETFMKIVKASFSYYLVLKNRQQIKFENDTF
CVDEC
>abi3052.t1_1
MCSEVSLAKVGAMIARNMNEYQYLRFNVKILKLLNLWAGPKGDGVLKWKQWRSYSLFIATLPTFLP
ILIECVLGFYDDDISLIIQMQSFSSAITIYGMLHMTFCFLRHSEQIKEIVNHVDIFKIYSALDVRK
VDRQATLYSKVLVLYTVFGTIFFSSIPVITRNHCEKNKFASMIKHGIPCGVITRFRLPFRYDKSPL
YEIFALHAVLVSGTASVLIINTTMLICGLILHVVNQLKQVRRMLMEIAYYSDNEIERAMKFAIKYH
IAIIEINEAFGSQLVVFATLSSSALGILGFETLMLTDFAEVLRFGLWLGGVFSIFFLICYFGQLLI
DESVGVANDVYSSRWYNTPLKTQKDVKLIIMRAQKPLTLNALNIGTFSAGTFLN
>abi3143.t1_1
MLSPISGSTDGTRPLPIQTWLPIDTSQSPTYEIVTFLLTVDTFFIVYTDAMFDYCLLFLAQHLLIQ
FLILKYMLTNIAEDGNLDHKFHSDSFQKNLFEKIRDCVQHHCLLLKFGKKLSKFGSLSLGIQLMFI
ILIMVVIEFLIYWDIENKSRCFKLILMFLAIFGQLVLYAYVGSELQATSVDIATAAFHSRWYSGRK
QVGDALVLMQLNAQKELKITASIFTVVNYGTIISVSKTALSIFTLLRSIQERDSS
>abi3425.t1_1
XSRESHRAKMTKSHCKEVLIVVNRMLSITGMWPLRNPSIFYRTRRFISCTLFITWYTSLIIAAFSV
NQDISNLNEILCTLVPATPYFAKGIALLFQEDAYISIVDDLYSEKFNNHYEKHDESLKKTIKVIDA
LSKIIRMSAFFTVIFYAFFPVIDGKPLPIPFSYDLGRFHYVMYAFQVVGITNAAWNNISFDTLIAS
FLGLAAAQIDILKNRLSDLYESGEEDVETIKKCVEFHISIISLVQRVEDIFSFGMLVQYLASLIVF
CNNGFQLLLIVEAFSFQFYLLIVFYVVLLSELSLYCWFGNEIYVKSVEIGQAYYMSYWYKCNLSTR
KYMFLVMERSKRPLKVTAQKFTTVSLESFVVATVSVQRAKYSIDKKMGRMTFHNAINPLHIICKIL
GLISFQITNKGYRTSKFGIVYVVTLIIIYIVLVVNAVIDGLPYKSSSLLSTTIDLMQISSSSVQVV
VSWTISVLYQSKFISFLTRMANLDAKFLKLGVWIYYEKIYKNIKIRLTVHFICNTLSILVQILMYD
DDLSLQMVTFYIAYYFPIFINMGVIELMSSYNNIIKSRFIILNQHLKNLKNENNDNLEKEKIFCVK
LTNVLGSKLSILRIICPIHHELTKITKLVNETFGIMVLMSFTVSFVTITTSLYFCNVFLLNLEENT
IRQVCALLTMCFTYIFQCMCLCYNCHSTVEAANEAGRLLHQIDTDDEDVQDQIEMFSLQIANEGLE
FSAAGFFPVNYSLAFSDNVPDHRGHYHIFGHFDSVLEKFE

>abi3542.t1_1
MLTEVIHVYKHFDKVEELTLTLCYLLAHMIGIGKVIVLTVKRKSIGKFLKSLEEDLFLPNVDRGGI
EEVNIIKSAVYQTNTQARIFGVAVVSMLSTRVVSCLMHSTTKRVNKNSPNETEVIPVFPYPSTIPF
IDDAGKSPYYEFEFFYQTFSIALFGWYLGNSDTIITGLMIHVTAQFRIAINAILTVTERAERITKI
DGNKSNKYFTYKDKNDYSYKIEKIEKYEEKLMENLRICINSCVRHHHEIIKLLDNMENTFSLLLLI
QFLGSLMALCVGLYQTSLLPMTNPSFVSMSSFTIAMLFQLLIYCWNGNEISLISAEVATASYNCKW
LQSDKNIKTSLLLIMMRSQRASFLTAGKFSKLSLETFITIVRGSVSYFMVLRKMNEQQVLNMENHV
ENN
>abi3607.t1_1
MKKLHLNFHDFLKTNVLILHYFSYWPLRDRSEWSKKLYVVYSILVVSITIHLYEISETINMIMVLG
DKDKMIESSLVLITNYAQLVKLFYLVTRNVRFQVMINRMNDEVFQSKTQRQWDIAVKNMKSCRRNT
MTLLLLGICTVSTWSVAPLIKYPREKKILFSGFYPYDTTKSPQFELTHLFQATSIIVHCITNLSMD
TIAVTVMAHICGQLDILKDFLINMNSYAADAIKNDELVTEKMNKLLIECIHRHWNILSLADELNSL
YTYAALAQFTGSVLIICIVMYQIQTYADATSALLLVRKRNYIEGKSTALMRMGITNTYATASSERN
LLSEDLSQSAYMCDWTECSIEFKRNLLFFMMGVRKPLKIYAGNFFILSLESYVSILRLSWSYLAVL
QSQV
>abi3700.t1_1
MMVISLGIIGTLIRRLMYSDSSKWELPFMPFSLIDTKVSPNYEIVWFYQGASRILYCMIVSSTDTL
IGAVLAHICAQCQLLQNSIRKLVENAYNESIEENEIFQQNIKNPEEIDPSLIPWHIMEKHTKQTIK
YHLAILSIANEFEEIFTILILCEFLATLFILCFCMYHASLFDLFSMRAAQDFSYVGAISVQIFLCC
FWGNEVTLQSQAIADACYEANFVGADLRFQKALIVIIRRCQRPIVLTAAKFTELSLKTYVWYGDLP
KAPSGDYLITDIRKLVSDMIINHEKIVPIANDSN
>abi4443.t1_1
MGTAFEHTTDVTDCKAILKAQKMSFNRRIVSNGGKNALICNYKLSTQLAQKKTMGDVEESYQYLAF
NIKILRLLNLWPEEGELDELKWKRWKTYIIFIATLPSTIPLLCEFAIVFKEVDLDIVGRIQTVLAT
FCVSGMFYMAVCFLQNSGKITEIVDRIKTFRKYTDLDMIQIDKKATLYSKIFFAYSCIGMLIYTVM
PLISSEYCEEHKSDRMKKYGIPCGVITRVRFPYKYDVSPMYEITALHQMFLAGTTTLIIIITITMMI
CGLLIHAINQLKELSGMLHDLGNADDVTESLRFAVAYHNEIISYIESINEAFGTQLILYITLTSMV
ISVLGFEILMVENFAESLRFGMHMLGWFVLFYLLCHYGQLLIDENSHDLKLSAITNISNDDGQIFK
FRVHF
>abi4791.t1_1
MDNIEALTTEIHAFAKYSIYIYYKRDFARFLDEMQYFWKVDNFETKLQIELNGIYKFTNMPLTSEE
NMLPLESWLADILIIDTLVLAMQFYFFYLALISIMGFDCIFAALSTHLVVQLKLLNCEFRSLYMDT
NGDLMSDRKAQQKLVKCVRILRRMTTMYSPVLLGQYFVTLSTTCMEMYILSESSEHINYIQILKAV
VYISALSWEFGVYCVSGEYIKHEISDLSNGIYHSKWYESKLKYQKDILFLMMATQKEVQFTASGFM
EVNVQTFASVFKAAFTFCTLMRSVLT
>abi5910.t1_1
XALDTSDPLISSLRLNTRVHQRKRLTLPPEVKELLYCEDIGETCATENLENMDDDEDDYPEELEEE
NTFLDNEFEIDKIFEHILFHQFGAAIVAICCGIFVINLAGFPPQLLILGKYIPILMAYHLQLFVVC
ISGEVLINQALSVSDVAYNANWHIKHQPKLTKGLMLVIQRTTRINKLKIGGIWDLNLATFVVVSTG
FLL
>abi6182.t1_1
MMRFSNMWPYENESTRTFYIKTFVKYSICVYVHIGIIGNLISSIVGMLKIGGIFLIYALSDFSLFL
IDNKNPGPDWIGSTGTDVTIFPITDDSLTLEEIIEGVYMTTCFTNLLFNSVCLVITGRESYKMFEQ
LKNFEKFGVPDNVAYVDKMVHYFIMGAFGFTFCAPLFLQLFVLMDTKRCKAHYAKLGRDRQCGFIV
RLAYPYDIKESPRWELHVMAVTFFSTFSCSTGAFLTTCCCVAIEFIILRIEHLKRLLRKVFDEDGY
EKRRQRLYHCITYHIHIIELSEELNKCFTLLLTPAVFNYSINIGVCLFHILLKTNSFKNDPRRCVK
LNHSIKEFHVADLSVNSVDRKKPDFNPELFWMX
>abi6753.t1_1
MPVHRYNPLKVKPKCFGKKGFSTIRQRCLENLNLPESYAILHDEDNWRLDVEMNSEMNKLTRRLQI
IISICDELEDIFLYAVFSEVFFVMIMIISVLYIISSKAVFSSVFISLMMYFITIMSQLIAYCVFGN
DVTLTFGTLTFSLYDSDWFSASKSLKKSLLINMCRLQKPVYFTIGKFSKLSLDSLVAKAPVLNCEL
VSTDERITKMSLPLITVHFHKRTTSPPFKRFSNFPPEHKLHVWEKRLHHCAMTSHHENFFFTNVRV
LKLSGLWTLLNLKQATFSALLYTWFTIIVCQFYHHPTEYYMLKFTYARLDDFIEQIGMLITHLLGS
LKVLFFYIHRKKIDALMDEMQNHRFQYEKTENFDPKLTVDKEKRLIHRATLIFLCLATGVPISKSI
PALYHLASLDHDDLLAKNVTCYDVLPYFSWIPFPTSTTFSCTLAILFQNVPMIYFAFQITAFDAMF
ASFLTCTKSHLFVLRGAFESIRSRCLKKLGLPGDYDDLYDENNPELEREMIAEMRIASVHLQTIIK
FAEDFEVIYTFVTLVQVLLSAFVLMSCLYMASTVPAFSGTFNAEVEYFSAVITQLTMYCWFGNEIT
LASASLPQGIYHGDWLSSSENFKKSMLINMLRMNKPIYITIGKFSPLTLSTLVSILRGSYSYFAVL
RRSFRETSFLKTADSTNAFSICARFASTSSKTVHDRFQFLTEGRRNDENFERRTKSGKCGQLCGWK

SPGCLGMFQKNTNGFGYADGFVKDPKLMPGGNYIKPGRDSTKSTHLIFSPPNEGVGMLARYLKVFE
KHQINLLHIESRPSARVSNKYEFVVECAPSGDLGAAITEIKDQSEYFSIISRNYADNLGILGLHFQ
CCKLQKKKQSTQR
>abi7056.t1_1
STSIAEAAYDTNWYEVETLLRKDVLFMMMRAQQPLTLNAGKFGCLSLPLLKQIISGAFTYITMRSA
SD
>abi7329.t1_1
MFYLLKAFRQGELPCDYFRMQVNVLRFFGIELFEDETLLYQICSKFALFVTVYLYTCVEVYEVYYQ
WGNMDDVLQILIYAFTHTLGLLKVTMLYTNRKKIGKMLKQFYEAPFAPSLDRDLNEEKRMIRNVIK
VTEIQIGRIPTSKSPQFEITCSYQIFCVLSMACIILSIDLLLGTILAHLSIQLKILQNAFKNMRKR
AQDVAAERNIKTGEYMATILRDYIEHHQKIFDLANDMETLCNKMFLVMMLGSVGILCFNLYLSTMI
GYICYWGNEVTFQSEGIASAVVEVDWPGAPLSFSKALVLIIARSQKTLHLTAGKFVPLSVTTFMGV
RLHILMRRRKMFWFLKAFRQEDLPGNYFRLQVGILRVFGIELFDEESALYKIYSKLALFVIVYLFT
SCEAYEFYYQWGNVEGMVQILSYLLTHALVYLNRKKIRKILRQLHEVPFAPSSERDWTHEKELIRN
VIRTTEIQSVTLYVIVLILLISGIVSSLKARFFKNRDQWHYAFASMDAISTTESPQFEIACFYQIF
CGITFAYIITSIDLLLGTILAHLSIQLKVLQHAFRNIKKAHALQNMEELGKYIGHHQKIFDLTDNM
ETLCNKMFLVIMVASVGVLCFILYRQSTFPIASMEFAQYFCYYWTVFCEIGCICYWGNEVTFQSEG
VATAVVEADWVGAPLTFSKALVLIIARSQMTLEVTAGKFVPLSLKAFMAILKGSFSYYMILRQSQE
KERH
>abi7945.t1_1
MFPLFHSPEGTPANYFNLQITVLKLLGISFTGDETWKYFICSSLWLGSIVITYTVIELYEVYTYIN
DLDTVVNILSYSGTDILGVIKISVLLWYRIKIGNTLNNLEKGYFAPNVERGGPIEETLIKKCILIC
NRQTAIYYFAVIMVVMFGGLGTLYHRFIVTKESDTWEMPFVPFSWFDVKKSPNYEIVWLYQVVWRL
LFAMIVSSTDSLIGCTLAHMSTQCQILQNAIRQMVVNAYKDMFKIHGHVDEKQLQDIDPSLISSTI
LQKHLKNIVDYHLAIINLAEKFEDLFSMLIFALFGATLFILCFIMYHASMFDLISVRAAQDFSYVG
VVMIQVFLYCFWGNEVTFQSQEVANACWETNFVGTDIRFQKGLALVIRRCQKPIVLTGGKFATLSV
ETYAW
>abi8074.t1_1
MEGNAFFHVNEVILKCAGLWIPNERAKSNVKFWYFSYNLVVVTFSCLIFTPSELLCFSDTASDLEA
LVANANTGMTHLLSNVKVCIWFYFRKEILGIIEILSDYAKIYESCEEFYPQEIITKEKKIKDIATK
SFLSACISVSVTACLASIKSLFMAPQMGMEGNGTNGTTTHLENFKLPYYSRIPFDYKSSSFGFAMA
IFYQCLGGFSFAWIIVGFDTLYVAILCYICAHFLVIQGAFKTIYQRCIQRLKIPEQLVIDDSSYLN
REMILEMNKTTRHLQVVLKICTRIEEIYTYIILAQMLISLIVFCTCLYLVSSAEDIPVKLYEGNWV
LANTSFKRSMMINMSRMLRPIYFTMGKFSPLTLATFVNISKGSYSFFAVLKNTNEAT
>abi9785.t1_1
MASLIKAIWQKELPENYFRVQVGILRTFGIELTKEETFYYKVYSRIALVVICYVFTASEAYELYSQ
SDDLNTTVTVLSYLVTHILGSIKTTLVLKNRKKIGGILRELHESPFKPDIKRGGQIEEKYVRDIVR
VSEIQAVIFLFIASATLSSGVVVFLKTRLVNTKDEWQYPFTPVTLFDTSYTPMFEIVSLYQSINMT
VFAFIICNMDILLATIMAHLSIQLKILQNALRNLRSKAGEMQKENNGKHDVDYYMKNLLGAYIQLH
KQIYTLVDDMENLCHLLFLFIMSASIFLLCFLLYQMSRFPFGSIEFIKNFCYYWVVFCEIGITCYW
GNEVTLESANVAQAAAEMDWPGAPPEVEKALVLVIARSQMPLQLTAGKFVPLSLETFMGMGKRGKC
LTIMCKNNILDELDKGAAITTLAIKYGVAKSTICALKKKKSAIRQSTMNAVGGLGNRKTLQSSHYV
AMETNDATAGCNGLRKGTAYASLKLSAKNFHLDQTX
>abi9811.t1_1
MNTSNTKKRPSPFDAFINERTIITFFGFPMGRKETNFKMVVKGTITLIISFSLIFSMGGNIIDKKD
NVPVILETLYFGLTQTTFLCKMVNFLMRKENYYKLEESLRKPIFNAFTVEQDIYIREAIDLSNLFA
KSFRNVVALTIMFYATFPFLEKGLPLPGWFPIDNDKYHYVLYIYQMVAIIINGFVHSSLDCVTAAH
ISLASAQFEILKHNLEHLKRDDDENLSESEIDELIQTRVKVSVDHHNEIIKCMFCEEGNDGREMKW
SKVGIGEDKGRSRNFTANEAEVGTGSVMSTTRLLINRYMSSATLRKLNIENVSTINQFRLKLENGD
YATIKYNERNDIYDLIHTDVPPALQGKGIGAIIAENNIYYLVESFVPDSYRGKGVGQKLAKVQINC
CFDSIKMDNYDVKPKIFYLLRSRLYDSMLKTCKEVVNNQLPDINVHFYHAVALVLNKRYQESIREL
NLLKLDTSTRLASFVTLMYVLKFMSTDDKELFINLDTDMRDARKNAEPVDFYNTSFALFALGKLEK
SLDYIERAINLDSNNGDFWTLKGWINIYLFQSGLKKNIIISDIFKNGFKDNLRNLDGYIGLSESLN
LEGNNEEAISEINKAVVRFPSNVLPLIYKLKLMLSMQDWEQTIELMNRVTDLEINELEALKTNILI
LLCRDGNYEEAAVCIKRFAQELEIKESKNSLPFIENAQLFSRVCGQNLSVLSEAFKLVEKGVQISP
NVPQFITEMGYQCILQGDIKEAIRHFKFATKIDDTSIDALMGMTLCEYMENGTSHNLEQQLDFLLE
LQENNVTPLLCFMKAKSLNDCNEKFKMLDQTYKEHIKLLERYKYGATYLRCLDPNFLLSVVKEYLQ
FVPFSMDSLNFKAKKTNDIVTTSLNILKLITKACPGLHEALYLLAKILYLTGDTVEAVSTLEHIIK
DLDDSSSEAHLLMAQIQIGTGMYGRAAQSLEAGLSHNFKVRENPLYHLISGSIEKNQNNLESAIKY
YTTALSLIGLKSELNRLNSPLDITLADKAFVYTELINAHMSLKQVHEATKLLQDATEEFKGTPEEA

KISLVSADHLVGNKDAQAAIDLLSKVKPNDPYYLAAKTKLANIFLKYRKDTHAYLKCYQEMVKNNP
GPESFVLLGDAYLCILEHEKALESYEKALKMNPTDPFLTSKMGKALVTTHFYRRAISYYKDTINLT
DDPELKLQLADLYIQLKQTDNAENLLKNELETEKNKNINDVTSLNYRTKLIMLLAQIYEKSGNLKD
ASVVYKNAIENHFRIHKLYSIEQKATPEDLMNTAVSINMKLAELASTLRDNQQAVTYYKDALNISP
NNCKVLAALAKMYMQMNIMDMCQHTCTTLLSVDPDNEDGTVLMADVAFRKQIDFDMAIYHFTQLLS
KQPTNWRALVRFIEIMRRTGYLQDVFKYIQQAEKLCPFPSKDPGFCFCTALYQWYSSNINCALRNF
NIARQHPDWSHQAIYNMIEICLNPEDEVLGEQFIDVDDTEYKDSRSMALKTAERLLKELKQKFDGT
GEDDLKCKLFKNFLLLATKEKANIEQGLEDFVALASQDRYKEQVGPILGVATAYTMLKQSQRAKNQ
LKRVVKTVWSFEDADYLERCWLLLADYYVQSTKFEMASDLLKRILQYNKACSKAHEYSGHIAEKEQ
RYKDAIVHYENAWKYSGRNNPVIGYKLAYNYMKCKKYADGIDVCHQVLKQHPEYPKIKKDILDKCM
NNLRT
>abi10062.t1_1
MVEAKENEPRTSLDLCLKVLRIFGYWPPKKGNADVLKRYKMYAIFAIGFWFATFVVTETIKMITVL
DDLEKITNGSFLLLTHFAHSVKLFYFIVFNQRIKKLVVGLNRQVFKPKNHRQIRMVANEMNYFVKF
CIFFLGMAIMTCALWAIFPFIESGDNGIELPISAWYPFDTEKHFKLIYLYQVAGATINAATNVSMD
CIVGGLMSQICLQLDLLNDSFRRLGNDFDQELDVWQSQMDEALINCIIHHKNIIEMTNEVDSVFSI
SLMSQFIVSVIILCITMFQLTV
>abi10321.t1_1
MNRPNCHQLFDRLIRMSKQSGIFPLKNPSMFYLTRRIFTMVVNFAFITTLFLEMINSREDVERMNE
VMCLFVPIVGHIGKVISFIWNKRSFEFIVDTLHGDEFNTRPEKLDKPLKETVYIVNFISNALISFV
IFTITFYVLSPLFEDGSLPIPFSYNLGRFHYLMYAFQILSLMVVGWNAYLVDLIYASTVSMAAAQI
DVLNGRISATVSEAMDVIDVTYKSGIGMQRDVDGKIRSVLRYCIGHHDIIAGSRKMYTHCQDIFQT
IKPMMNMTGIWPNSSKISFLKRSVMLTLSIVFFGTLCTEMFVANFEQLNEVVIFILLMMSYFGKLF
FFLCKSGVLLSLLDQLKSDIFNVHPYNFNLNVKRAVLNVKNLSKVFMISVMCTMFFYVASPILSQK
DLALPFSFELGKYTWYMHVFQSTGLAIFAWNMLFIDLSYVGFMSISIAQIDILKGRISEMVQEVRK
DRTLLMKHKQVYNKIIDVKLNVIIIHCVDHHVAIMDFVKQVEQIYSGPLLMQYTTTIIVICNVASQ
LLLNKESTSQLSIFCLFGAILIELSLFHWFGNELIIKSSQLGEACYNSQWYDCSISVRKSLLLIME
RAKRPLAISVFKFTTVSLESFVTAPDLHEFRKLIILLSSLRDSLIVDLGDCSSLPSTLIFKRISSH
IKIEQINLKVILSRANADPLSTKISNKRLSIFSGQEKGSSRLTLADTVEDVF
>abi10322.t1_1
MVQINETYKIQRTMMSITGVWHVENASFFYRSRCVFTWSIFMIAFVGVIMEFFNDLTNLEKLCQIL
YMMLSYTSYFCKMVGFTYNKKTFLAMLNHLNDPLFVLCPREMEKHVSRVMNTSILLTKMYRFACGL
TVMLFTVYPVFDNKSLPMPFPFELGRYTPLMYAIQSVALMIAAWNNFCLDTLCVSLMGLAYSEFDV
LKYKIINVGESTLKKLRNEMRNEFFDDTNPKINEIVNKMLNNCIQHHCAIITLHHQTTRTTKLDTT
REENKEIVEEAKKDDVQTEPKINKNDIDIAKIVSKLEECTKEQKFFENRRANFKNRNQKPRYGDVH
QSGFKQYGPRRTFDKKSLLIEENDITKDENPIKETKIEETSKNTTTQTYKQSTFNNSSPKKYYQNN
LTLNYSEILKKSATRNRHVKDFFALSKKPKLWRSKPNRLRNKRRLQNPPTNGFPFLNAKQNLHKNI
DLSAIMKNATYGRNRSIDIDLSKFDVPENEP
>abi10705.t1_1
MIKCFNLALTILMGSAKSLFWFFKGKHMENVMDSIESEEFHYEKLGNFDPGSLTKEAKRTGLKYGL
AFFILGQLTLDMLYVPAYSLSFWHLIKNKPITNVTTFQKFPYYTYIPFRHDTPFMYMLALTHQCIP
FYMFSFSLIGIDTLFMNIMNFVGVQFVILQGAFRTIRLRSLKMIKSPPLTKDGLFNSHELEGKMLS
ELKKSIKHLQKMIWVCEELERNYTYITLGQVLSTLFILCSSLLQISTASDIPQALWESDWGRPKKP
WNKTIQTILEQTGENWNSDKEKAKEWSKFYNVEELEVRVKEDWIII
>abi13094.t1_1
MPDFFAINVRILKWSGLWVPSKTDATYKPRIFYNTVCVFYSIIFFTIAEFVALRESSKNLDDLIKN
LNMSLSFLLTFFKVCVWFYHRNDIMDIMETLQKNSARFERIEDFDPAIIVAKEKKIKDMLTMCFFT
LSFMVPTSACLATFTDLFSSTKVDVYNGTEEANLPKLPYYSWVPFEYKTSKTLYGIAVVYQCLALL
ICGTITVGLDMLYTALVSFMSSHFKIIRGAFKSIRPTCIRRLNIPNKTVLRDSYELSHEMEMEMKN
CVRYLQTLFRLCEMLERIYSNLILMQVLISLVVLCTCLFLVSSIPVGKEMLGNELAYLLAIENQLA
VYCYVGNKVTVSADGIPFAIYEGDWFSTTKSFKTSMMITMMRMRRPVYLTIGKFTPLTLNTFIGRG
SYSLLAVLKTE
>abi14688.t1_1
MDLKLYDDLKLSEPYVAGFKVLQILGHWPPENGSLKTVRLYRIYSIFAVCFWFGCSFLTEMIKMIV
VMDDLEQATSTSFLFLTHVSQFVKLTYFIVYKERIKKLVVSLDRPVFKPKTHRQFKIATDRMNFFD
KGFKLTLAMPMVTCVFWAIFPFLESDDRVQLPISAWYPFDTDKFFKWVFLYQIVGVTINAATNCCF
DTTISGMMNHICGQLDMLNDALEYLGHDFTKDETVNWQHQMDEALKACIIHHKNIIEMKNEVSFLF
NFPIMGQFTVSVIIICLTMFQISLVSIASIKFLTLVLYQGCMLLEITLFCWFGNEIILKSSQLSTS
VYNANWTDCSTSFKKNLLFLMMHTKDPLEIRAGNFVGVSLQTLSGIVRTSWSYFVVLQQVHDE
>abi14834.t1_1

MSKIPITKILDDRAKWIMIMPKYILESVNLWPEQKNIFTNVTFFMMILICILMEIGQFVYFIFHIK
DIPALTKIMSTLSVTFQAITKMVTIFINADELNSMIKKIWNEFWPSDLLGKSANLPRVPKLLYANL
FFVYISGMTFGVGFLLFPFIKGNFDLPYEAWYPFDYRKSPYYELIYVVQFFMNIHIVMNTICGHDY
LFVAMCNNCVAQFELLKEVFGKIGTGTEKELNDKLCKIPGFFCGNSALEEKKLLVICVAHHQKLIT
>abi15256.t1_1
MEDEPKPEEFKIRKLSFEFTRAIYHDKVKNTLLLSKWLLQGLCAWPDNNEKYIKFINILSLVNFFI
LETLHVLFVLSVYDDIATAADATTTVTAVVEGIVRTLIIVIKKKEINNMLCKVWFDFWPIKLIEPI
YAKKLVTKMRMTFMLPGGYLLLSIVCNTQIVGDPFVHHSGLIFQSIYPFKWNETYVYEALFFWQYI
GDWFTLFMINAFDFFMVTLVGIVIIQYMMLHEVIREILTEKSKRHRAVIFGEGWENVTDLEVLLKC
LEQHQTLIDQLESTFTFVILIQFFNSTSAICMSALVLKVNVNQFFKMLTFVTAHILQLFYYCHVGN
ELKFQSSALADAIYECNWNHQYNRKFRKALVLMIQRAQNAQCLTAAGITELDFAGFLRVMRISFSF
YTFLDTMMQEK
>abi15610.t1_1
MKKHVHLKINMMFMYIFGVFKFKFKNVFYTHLNWSYSNFVKIYLITFVCTQYTQFFQILDEDLFKI
VSNLSVSLLYTVAILKMLACDGKKIQLLVNEIYDTERKILSHGNEEAKEIFFRYVRWNYFIDKLLI
WVGVCTLLPYYIGPVLEEMTIETEIFNQTINNVTRQFLKRPLPFSSWFPFNRYRYYYVCYGYQMLA
GFIGGSMTVATDIFFVGLMIFAKGQIKILQHCLRSSKILAVRLAKVDKNISLEQAIQHVVKHCIIM
HQIIIKYVEDLNTSLKTLMLCDFGITSIQMATVGLQIITVGVGFNMMFAVEFLGAMFIQLFLFYWH
ANEILLESLEISTAIWESAWFEYNQSMKKSLALVMMRSQRPLVLSVGPFYNMSTDTMISVS
>abi16946.t1_1
MFYMAFCFLRNGEQIKEIVDELDKFKSYSDLDITEVDKQATFYSKVLFVYAIIGVLFYTCTPLLTL
DYCERHKFISMQKYGIPCGLITRFRQPFPYNTSPLFEVYVFHSIFVAGTTTIIIVTITMLICGLLL
HVISQLKQVRILMLEVSNLPRAESVKSLNYAVQYHIVIIKYLDKVNTAFGSQLIMYITLTSFVISV
LGFEVLMVKDFAESIRFGMHLAGWFALLYLICHFGQRLIDESIGIAQDIYYTPWYDSPLVVQKDIK
LILLRAQKPLTLNAINLGIFSAATFLRVRV
>abi18302.t1_1
MANLIYNFWTLAFMHIVYFDDGQSRRHRPPKKMDRPDHDALDRFFEVNVFILKTGGVWIPSKNRPF
RERFLRYFYNFSLLSYCLLIYQPAETGVLFTDVSLIVFIRSLRDQFNHFICVYKIFRWFGKRTEIL
DIMRVLQSKEFTYEDYGDFRPAMIIKDHKEKADRWMKMFLYGVNGICFNMCVSVFYVFLFKHEDFY
RKDDNGNLIYDQKLPVTLTTPFRQDTRLGFLLHFIFEIFPLDIYGWIIIGLDTLFTSIMSCISAHL
CILQGAFSTIRLRCLRRLGLPDDEVLHNTTEIDVEMLNEINKISERLERVYNIQTCGQVLISLFEM
CFCLYLLSLAFNESFGNELTYLFSTAFELLLYCWFGNEVTEASAAIPEALFKSDWLSADVKFKKNM
LFTMTRMSKPIYLTIAKFTPMAFTTFLSIARVAYSFYTVLRSGVIE
>abi18303.t1_1
MDVLTSDDFQVSYSKLIDMNKRKNELVCKFLFATGMGISVSRPFLAFLAWKDRTDEDFKIGNVTCE
EILPYSSFVPFSTGTTFSCGAGIFLQFICTHFFILIQESLDSFFASLLFCVETHLQIIGKGFSTIK
ERCLEKLNSSSEIHDKDNWKLDAEMNWEMRKSTRSLQTLIRICQELEDIYTYVVFCQVFIMMTSAL
YIMSKIIVYSGQFVSLMTYFLGVTSELGAYCWFGNEVTFASEILPNSLYDCEWLSASNSFKRSLLI
NMCRLQRPIHFTIGKFSKLSFVLFVSAIRTSYSSQI
>abi19036.t1_1
MAIESNDFFYANVVILKWAGLWIPHKNVKPSLKRWYYIYNAFWITFSCVIFTQLELLSFEKTASNL
EDLVKNVNMGMTHFLANIKVLLWFYKRKEILEILEILENYSGFYEDCGDFEPKKILLKEKKLKDIA
TKGFLGTALSVSTTACLATLALLLLDNDVHYVQNAKNETVLNLSFDTFYVAILSYICAHLLIIQGA
FKTIRARCLQKFNLPEELDIYNSDILNKYMNEEMNIITKHLQVILNVCSRLENIYTYITLAQILIS
LVVFCTCLFMVSSIPITSAKFGTELFYLIAIENQLAIYCVFGNKVTLSVRKPISLFLNFC
>abi19141.t1_1
MRFLDDRALFSELIDGLYTVQCAANLQFNYFGMVYRHVPAMRLFEELQNFDNFGVPKQLARTARKV
TYVNRGLFTYCAGGPFGVCLFNFVVRQACLAQNEKTGREETCGYTTYAVYPYSIAASPFFEIHLFI
STFYVVYMCGGGTTFACACYTAVEYINVRIEHLKSMLRKLSQVKSHQRDEDFRFCVRYHQYIISLT
EQTNKCFSIILTPALFLYSVNIGLSLFHFLLDHSCKGLFNCIGWLSLTAVVCIIGDELIDKSMSLA
FEAYGTNWYSFDIRLKKDIMFMIRRAQEPLTLRVGSFGHLSLPLLKQVDVIIDVLKYSQKSKVREY
GQKRATKNLQKYIDDLPTDEILNVIKTGINRQDFSSFLNYLLLAFTDTTESHEKRFRVFETVLEEL
FKRNVSTSNSNTITTRLSMELEKLKSEHLVALCSVCTERIQKGNIADTSWQELLPKLLQVLIEREC
VEYNDITVTGLEYKTQYVNSLCMLQWSPNIVIPLTAMFIEIPLMKDEHLQVVSKLGQYMEKLTCQE
IPSFVYQFLKLCQYQNGRSIFLLLQNYFGIRIYNRVQKDNNFTSSETIDLIDDTLDDQAIQAESTV
LFHIYQSALHGYDSIKDYLSSLKNLTKSPEFVLHPFQLTALLTISTVTFYEDRVFTIIRTCISRNF
HEEIKKSESLWFKDMIPLPVNMNAVIKQIIEFSVQERDLVLQGLVNLGFILLGVGLQLGKNTLVEK
QWTLGKFILLSLIKQKPHIAHAILQRLCNCIISGQNASQCIDCFHSLTRKFALITLENKSCVVELM
EALLQASGSVATSILDAVIPLTKLSPTIRDHLILLLRKALYSRITETRQMAVKGFLKMLKNLKISN
MAALSQMSSSNSSTSHSILTQISLTRAGQSATSVFTNEALCFELLGILRRCFMQQAEVRARLYDGL

YDAVCMNPDIGEPVLNLLWSHFGDYYVIDQDVLPPLQFSKITLTKDVDVTLQEPLGKLIHTFGIIV
TKVLESDEENASAAKFAKILGSLCDRMSKCELVHLELDDGTNLLDVLPECQQKLIILKEAMCVYEA
LIGFKLSSWRKTSENYAQDVNRLFQGYSRLLQFFKNLSKPRKGGRKKKDVNKTTTQQSESSSKHDS
QLKAKSIKLSNTLLDMRVLIKFLHLLHGNVNWTTVSQANLLKPKHGIHRHMMQATIQQFQAIKNEK
FPGHHDSKLYYNYCTDVGSILYERCIVRFEDFLDFDCTTAILTLDCFHIILQLICSNYHDDLKLFL
SLISGNESEEDFLVQLKSFIEIYQKLFELDEDDAPTDPEIKKLPQIILSTLTLLVEQIPTEINDLS
IQVYWMKQFACRKTVNNKLITTNFATLWFNTYLKFKSGTMIFEKIVDNLHEVAGTMGDVSFTRGLE
NKTITNFKAVEVREKFNLVNELSAHSLIQCLTHVLKVMLDDAEWIISRLRSEYLMFTYPGEQGSDR
RREYLKNKERGTCCHMCLLISLVNSLCNVLIPTGVLSENVLKVLALLYNTLNSLAKYFLLRSTKVN
VAFQPARFEAVVKMVGKQLSPNVHKFILNIEDMQKEEMGNPDVKKKSVDSLKTKVLRETKLIPKVV
FEIEQLSKTVIQLSNKTKVDLSKFIGQGTTRDFRIKDLKSVMEKNQGLGDVSLSTDVPCSNQTVDD
NSNSESDECPPSKKSKI
>abi19497.t1_1
XWTSITFVGDSVHTRKDPDNENETITEEIPRLLIKSFYPWDAMSGTAHYLSLAYQIYYVLFSMLHA
NLMDVMFCSWLIFACEQLQHLKEIMKPLMELSATLDTYVPKSADLFRASSANSQDNLIENDYNNMK
NEELMLKGVYSTHQEMGGHFRSGALQQFGQGTGIGPNGLTKKQELMVRSAIKYWVERHKHVVRLVT
AIGDAYGIALLLHMLTSTITLTLLAYQATKIDGVNKYAATVLGYLFYALSQVFLFCIFGNRLIEES
SSVMEAAYSCHWYDGSEEAKTFVQIVCQQCQKAMSISGAKFFTISLDLFASSLAFWKTMHNNQQDG
RWALHLEEFNYTTVHTLGSRVKHVDTLSLYSIVTISTENSLLAKIHNDQEINAEFRTINY
>abi19794.t1_1
MGVCKITLTLFYKGRIHNYCQSLEVGNFLPNMSRGGQKEFEIVKNAIQIYNRQACFFYVIVLSIVT
QSTLHAFLDPGHDEISIDSLNNITNIKHIRLTPFSIWLPFKPVKTPLYEIASLYQIACGLFFGFVI
GAIDAIICGMYLHIKAQFLILKNVLDNYLKIAANLIENVDIDVSIYENSKHLSNGIHIVEKIPPIL
QKYVDIVVKGCVEHHQDIIDLSEGVEDTFCNLX
>abi21034.t1_1
MVDNFTESLRFGMHLAGWFVLLFLICHYGQLLIDESIGVANDAYELPWYNQSTRIQKDISMVIMRA
QKPLTLKAIDLGTISATTFLGVLSSAYSYFTLLLNLKK
>abi24016.t1_1
XNQNEPRTSLDLCLKVLRIFGYWPPEKGNADILYKMYTIFAIGFWFVTFVVTETIKMIMVLDDLEK
ITNGSFLLLTHFAQSVKIFYFIVFNRRIKKLVVGLNRQVFKPKNHRQIRMVANEMNYFVKFCIFFL
GVTIMTCGTWAIYPFIESGDNGIELPISAWYPFDTEKHFKLIYLYQVAGGTINGVTNISMDCIVGG
LMSQICLQLDLLNDSFRRLGNDFDQELDVWQSQMDEALINCIIHHKNIIE
>hom1520.t1_1
MDIENNDFFHVNIVILKWAGIWIPTKNISTKLKAWYIVYNSFWITYSCIIFTPFELLSFQNTASDL
NELIRNVNMGMTHFLALIKISIWLYRRNEILNIIGVLGEYSRIYETFDVFDVDKIIAKERRFKDIA
TKGFLISALSVSVTACLAALKNLFLASVETDSYESNNVTDSFLVSRNLPYHSLIPFNYTTSKLRFV
MAIFYQCFGVFNFGWIIVGLDTLYVAMLSYICAHLQIIQFAFKTIRPRCLKKMGLPHVEVLHDSDF
VNNEMTLEMNKITRHLQIILK
>hom2022.t1_1
XAFIDETLPENYFRIEVNILRFLGIELLKEESWSYAIYSKLALFYIVYSYTCGETYELYSQWGDLN
AVVNVLNYLFTHMLGAIKVTLLYLNRKKIGNIIKVLHKVPFTPDVTRGGQFEENYIRKIVRTTEIQ
YVVYFSIISMTVNCGIINFFKKRLFDVSEKWAYPYVPITLIDTTYSPVFEIACMYQISCVFTYAGI
IGTIDVLLSVIMAHLSVQLKILQNAFRHVRSRINTMRKNDVVKQPYGHYAHIVLGDNIKHHLQTLE
LVNEMEDICYVMFLVILLASVLQLCFLLYQTSLLPVKSVVFIQNFFYYWIVFLQVGMNCYWGNEVT
LEAARMADAATDFDWTDLPTYLSKALVLVVARSQKPLYITAGKFVPLSLPAFMSILKGSFSYFMVM
RRSQEEEE
>hom2023.t1_1
MPKKEKEAPHKIEEWMKGQNNKNDKEMDQARKMEKRKEHREKKVNNGRTSSNKTENTKQENKLQIL
ETRNNDESGKEQELLYEFNKVGLPDYNRNLKKGQREIKLEGGLILVYSSVTKEKRAAARVGCTNHK
IHKWIVEMVTILRMLGIELVKEESLGYKIYSRTILFYLIYSYTFVEALEIYSQWGDMNAIIGVCSF
LVTHITGVIKTTLLFANRKKIGNMLKTLHKKPFLPDENRGGEHEEIYVRNVIRSTELMLVVYFAIV
SVTLFTGVITFGWTRIFGKKDEWEYPYVKITIIDTSYSPTFEIACIYQISCVMFAAPIIASIYCLL
STMIAHLSVQMKILQNAFRNTRKQAQGMQDKNNKFTEIDYMTSLLGEYVEQHLQVFEFAKELEDLC
NIMILLIVLASGIMLCFNLYQSSKTEIGSITFIHYSSYYLIIVNQIYMYCHWGDKVTVESANVAQA
VAEVDWVDAPISVKKALILVIARSQRPLRMTAGKFVPLSIETFMKVNTRLNFRREIN
>hom2711.t1_1
MIARNMNEYQYLRFNVKILKLLNLWAGPKGDGVLKWKQWRSYSLFIATLPTFLPILIECVLGFYDD
DISLIIQMQSFSSAITIYGMLHMTFCFLRHSEQIKEIVNHVDIFKIYSALDVRKVDRQATLYSKVL
VLYTVFGTIFFSSIPVITRNHCEKNKFASMIKHGIPCGVITRFRLPFRYDKSPLYEIFALHAVLVS

GTASVLIINTTMLICGLILHVVNQLKQVRRMLMEIAYYSDNEIERAMKFAIKYHIAIIEINEAFGS
QLVVFATLSSSALGILGFETLMLTDFAEVLRFGLWLGX
>hom3159.t1_1
MGMKSWNRHMRLCSRHFAPTAYTNTSCTTLNRIAVPSLYPSVPRENQSHSPSPSCSPSVNSPPTSP
SEHTPKQTCLTFREKCYINVIKTKQEHLQKLKSLCRRGGNNLKNFLKISKCKIIRDIFQGMSSRCF
RFMLSKMRRVRAPESKKWSIDDKAVALDVYKQNPRLYSLLRRILLLPSKHTLFCLLQQISFKPGIN
EPLIRHLAACLNNGCDADRICILMFDKIKIKRHIQFNHSADCIVGFEEFGKQRRTDQTATHALVFM
AQGVHRNWKQPIAYYFVNNISSSYLEHILSNVLDALAGAKFRVVATVCDMATVNIKTLSSMGSSLT
SNYILHNGIRIHTIFDPPHLLKATRNLFQKYPVRITIPFLRSEQQLEARWQDIRTAFEVDKQNQFR
SLYKIKDVHMLPRGRFKRQVKLAAQVFSSSVASFINVLTSCGKVPQRALATATYVHQLNELFDSVN
GQSPVIPDGKYIGGWLKNKSPHLSYWDKVQETINNWQFVKDPESTRRRWRPRTQIGWIRSIKALKQ
IWHSVKKQGITYLRTRTFNQDSLENLFAGIRSSYDNKGNPTTLQFVNFLKIQIINDLPYQRFQGIS
CEDDDAKLLSNLRIFLNKIDCNTCNSKSVNENYDLISQFANDVYLSMSKSNYKMLSIAYFSCVIAT
RLLVNNNCDICHSNLTSSPTLPCDQFISLNEFSDQRNKLTFPSEKLVVTVGIGKVIVLTVKRKSIG
KFLKSLEEDLFLPNVDRGGIEEVNIIKSAVYQTNTQARIFGVAVVSMLSTRVVSCLMHSTTKRVNK
NSPNETEVIPVFPYPSTIPFIDDAGKSPYYEFEFFYQTFSIALFGWYLGNSDTIITGLMIHVTAQF
RIAINAILTVTERAERITKIDGNKSNKYFTYKDKNDYSYKIEKIEKYEEKLMENLRICINSCVRHH
HEIIKLLDNMENTFSLLLLIQFLGSLMALCVGLYQTSLLPMTNPSFVSMSSFTIAMLFQLLIYCWN
GNEISLISAEVATASYNCKWLQSDKNIKTSLLLIMMRSQRASFLTAGKFSKLSLETFITIVRGSVS
YFMVLRKMNEQQVLNMENHVENN
>hom3945.t1_1
MGTAFEHTTDVTDCKAILKAQKMSFNRRIVSNGGKNALICNYKLSTQLAQKKTMGDVEESYQYLAF
NIKILRLLNLWPEEGELDELKWKRWKTYIIFIATLPSTIPLLCEFAIVFKEVDLDIVGRIQTVLAT
FCVSGMFYMAVCFLQNSGKITEIVDRIKTFRKYTDLDMIQIDKKATLYSKIFFAYSCIGMLIYTVM
PLISSEYCEEHKSDRMKKYGIPCGVITRVRFPYKYDVSPMYEITALHQMFLAGTTTLIIITITMMI
CGLLIHAINQLKELSGMLHDLGNADDVTESLRFAVAYHNEIISYIESINEAFGTQLILYITLTSMV
ISVLGFEILMVENFAESLRFGMHMLGWFVLFYLLCHYGQLLIDE
>hom5498.t1_1
MMRFSNMWPYENESTRTFYIKTFVKYSICVYVHIGIIGNLISSIVGMLKIGGIFLIYALSDFSLFL
IDNKNPGPDWIGSTGTDVTIFPITDDSLTLEEIIEGVYMTTCFTNLLFNSVCLVITGRESYKMFEQ
LKNFEKFGVPDNVAYVDKMVHYFIMGAFGFTFCAPLFLQLFVLMDTKRCKAHYAKLGRDRQCGFIV
RLAYPYDIKESPRWELHVMAVTFFSTFSCSTGAFLTTCCCVAIEFIILRIEHLKRLLRKVFDEDGY
EKRRQRLYHCITYHIHIIELSEELNKCFTLLLTPAVFNYSINIGVCLFHILL
>hom6019.t1_1
MPVHRYNPLKVKPKCFGKKGFSTIRQRCLENLNLPESYAILHDEDNWRLDVEMNSEMNKLTRRLQI
IISICDELEDIFLYAVFSEVFFVMIMIISVLYIISSKAVFSSVFISLMMYFITIMSQLIAYCVFGN
DVTLTFGTLTFSLYDSDWFSASKSLKKSLLINMCRLQKPVYFTIGKFSKLSLDSLVATTSPPFKRF
SNFPPEHKLHVWEKRLHHCAMTSHHENFFFTNVRVLKLSGLWTLLNLKQATFSALLYTWFTIIVCQ
FYHHPTEYYMLKFTYARLDDFIEQIGMLITHLLGSLKVLFFYIHRKKIDALMDEMQNHRFQYEKTE
NFDPKLTVDKEKRLIHRATLIFLCLATGVPISKSIPALYHLASLDHDDLLAKNVTCYDVLPYFSWI
PFPTSTTFSCTLAILFQNVPMIYFAFQITAFDAMFASFLTCTKSHLFVLRGAFESIRSRCLKKLGL
PGDYDDLYDENNPELEREMIAEMRIASVHLQTIIKFAEDFEVIYTFVTLVQVLLSAFVLMSCLYMA
STVPAFSGTFNAEVEYFSAVITQLTMYCWFGNEITLASASLPQGIYHGDWLSSSENFKKSMLINML
RMNKPIYITIGKFSPLTLSTLVSILRGSYSYFAVLRSNQEE
>hom6529.t1_1
MFYLLKAFRQGELPCDYFRMQVNVLRFFGIELFEDETLLYQICSKFALFVTVYLYTCVEVYEVYYQ
WGNMDDVLQILIYAFTHTLGLLKVTMLYTNRKKIGKMLKQFYEAPFAPSLDRDLNEEKRMIRNVIK
VTEIQIGRIPTSKSPQFEITCSYQIFCVLSMACIILSIDLLLGTILAHLSIQLKILQNAFKNMRKR
AQDVAAERNIKTGEYMATILRDYIEHHQKIFDLANDMETLCNKMFLVMMLGSVGILCFNLYLSTMI
GYICYWGNEVTFQSEGIASAVVEVDWPGAPLSFSKALVLIIARSQKTLHLTAGKFVPLSVTTFMGV
GILRVFGIELFDEESALYKIYSKLALFVIVYLFTSCEAYEFYYQWGNVEGMVQILSYLLTHALVYL
NRKKIRKILRQLHEVPFAPSSERDWTHEKELIRNVIRTTEIQSVTLYVIVLILLISGIVSSLKARF
FKNRDQWHYAFASMDAISTTESPQFEIACFYQIFCGITFAYIITSIDLLLGTILAHLSIQLKVLQH
AFRNIKKAHALQNMEELGKYIGHHQKIFDLTDNMETLCNKMFLVIMVASVGVLCFILYRQSTFPIA
SMEFAQYFCYYWTVFCEIGCICYWGNEVTFQSEGVATAVVEADWVGAPLTFSKALVLIIARSQMTL
EVTAGKFVPLSLKAFMAVSIT
>hom7088.t1_1
MFGGLGTLYHRFIVTKESDTWEMPFVPFSWFDVKKSPNYEIVWLYQVVWRLLFAMIVSSTDSLIGC
TLAHMSTQCQILQNAIRQMVVNAYKDMFKIHGHVDEKQLQDIDPSLISSTILQKHLKNIVDYHLAI

INLAEKFEDLFSMLIFALFGATLFILCFIMYHASMFDLISVRAAQDFSYVGVVMIQVFLYCFWGNE
VTFQSQEVANACWETNFVGTDIRFQKGLALVIRRCQKPIVLTGGKFATLSVETYAW
>hom7206.t1_1
MEGNAFFHVNEVILKCAGLWIPNERAKSNVKFWYFSYNLVVVTFSCLIFTPSELLCFSDTASDLEA
LVANANTGMTHLLSNVKVCIWFYFRKEILGIIEILSDYAKIYESCEEFYPQEIITKEKKIKDIATK
SFLSACISVSVTACLASIKSLFMAPQMGMEGNGTNGTTTHLENFKLPYYSRIPFDYKSSSFGFAMA
IFYQCLGGFSFAWIIVGFDTLYVAILCYICAHFLVIQGAFKTIYQRCIQRLKIPEQLVIDDSSYLN
REMILEMNKTTRHLQVVLKYVVPYL
>hom8684.t1_1
MASLIKAIWQKELPENYFRVQVSPNIFPNGNNSKFQVGILRTFGIELTKEETFYYKVYSRIALVVI
CYVFTASEAYELYSQSDDLNTTVTVLSYLVTHILGSIKTTLVLKNRKKIGGILRELHESPFKPDIK
RGGQIEEKYVRDIVRVSEIQAVIFLFIASATLSSGVVVFLKTRLVNTKDEWQYPFTPVTLFDTSYT
PMFEIVSLYQSINMTVFAFIICNMDILLATIMAHLSIQLKILQNALRNLRSKAGEMQKENNGKHDV
DYYMKNLLGAYIQLHKQIYTLVDDMENLCHLLFLFIMSASIFLLCFLLYQMSRFPFGSIEFIKNFC
YYWVVFCEIGITCYWGNEVTLESANVAQAAAEMDWPGAPPEVEKALVLVIARSQMPLQLTAGKFVP
LSLETFMGETEKLCKAPITLQWKQSYTNGFLNNETNICQFREKYLMKAKEFYSTTYMNDKFTASDA
TAGCNGLRKGTAYASLKLSAKNFHLDQTX
>hom8706.t1_1
MNTSNTKKRPSPFDAFINERTIITFFGFPMGRKETNFKMVVKGTITLIISFSLIFSMGGNIIDKKD
NVPVILETLYFGLTQTTFLCKMVNFLMRKENYYKLEESLRKPIFNAFTVEQDIYIREAIDLSNLFA
KSFRNVVALTIMFYATFPFLEKGLPLPGWFPIDNDKYHYVLYIYQMVAIIINGFVHSSLDCVTAAH
ISLASAQFEILKHNLEHLKRDDDENLSESEIDELIQTRVKVSVDHHNEIIKYTKNV
>hom8912.t1_1
MVEAKENEPRTSLDLCLKVLRIFGYWPPKKGNADVLKRYKMYAIFAIGFWFATFVVTETIKMITVL
DDLEKITNGSFLLLTHFAHSVKLFYFIVFNQRIKKLVVGLNRQVFKPKNHRQIRMVANEMNYFVKF
CIFFLGMAIMTCALWAIFPFIESGDNGIELPISAWYPFDTEKHFKLIYLYQVAGATINAATNVSMD
CIVGGLMSQICLQLDLLNDSFRRLGNDFDQELDVWQSQMDEALINX
>hom9153.t1_1
MNRPNCHQLFDRLIRMSKQSGIFPLKNPSMFYLTRRIFTMVVNFAFITTLFLEMINSREDVERMNE
VMCLFVPIVGHIGKVISFIWNKRSFEFIVDTLHGDEFNTRPEKLDKPLKETVYIVNFISNALISFV
IFTITFYVLSPLFEDGSLPIPFSYNLGRFHYLMYAFQILSLMVVGWNAYLVDLIYASTVSMAAAQI
DVLNGRISATVSEAMDVIDVTYKSGIGMQRDVDGKIRSVLRYCIGHHDIIAGSRKMYTHCQDIFQT
IKPMMNMTGIWPNSSKISFLKRSVMLTLSIVFFGTLCTEMFVANFEQLNEVVIFILLMMSYFGKLF
FFLCKSGVLLSLLDQLKSDIFNVHPYNFNLNVKRAVLNVKNLSKVFMISVMCTMFFYVASPILSQK
DLALPFSFELGKYTWYMHVFQSTGLAIFAWNMLFIDLSYVGFMSISIAQIDILKGRISEMVQEVRK
DRTLLMKHKQVYNKIIDVKLNVIIIHCVDHHVAIMDFVKQVEQIYSGPLLMQYTTTIIVICNVASQ
LLLNKESTSQLSIFCLFGAILIELSLFHWFGNELIIKSSQLGEACYNSQWYDCSISVRKSLLLIME
RAKRPLAISVFKFTTVSLESFVTVR
>hom9487.t1_1
MIKCFNLALTILMGSAKSLFWFFKGKHMENVMDSIESEEFHYEKLGNFDPGSLTKEAKRTGLKYGL
AFFILGQLTLDMLYVPAYSLSFWHLIKNKPITNVTTFQKFPYYTYIPFRHDTPFMYMLALTHQCIP
FYMFSFSLIGIDTLFMNIMNFVGVQFVILQGAFRTIRLRSLKMIKSPPLTKDGLFNSHELEGKMLS
ELKKSIKHLQKMIWGRPKKPWNKTIQTILEQTGENWNSDKEKAKEWSKFYNVEELEVRVKEDWIII
>hom12362.t1_1
VLAKLLFAFVLMPKYVSLLKKTESLWPDNIFGNALKEELDKEKRLFEKLFRMYRVTIMSTAFIYTT
KPLVLFTKTLPTVWYVPCDTEKLACYVVVVVTSQCLWVYLLGMSIVCYDAFFFGILYQAYKELEKIK
YGFKNFEVNENKEKIDEAQMQKKFASIVEYHNYILE
>hom12980.t1_1
MDLKLYDDLKLSEPYVAGFKVLQILGHWPPENGSLKTVRLYRIYSIFAVCFWFGCSFLTEMIKMIV
VMDDLEQATSTSFLFLTHVSQFVKLTYFIVYKERIKKLVVSLDRPVFKPKTHRQFKIATDRMNFFD
KGFKLTLAMPMVTCVFWAIFPFLESDDRVQLPISAWYPFDTDKFFKWVFLYQIVGVTINAATNCCF
DTTISGMMNHICGQLDMLNDALEYLGHDFTKDETVNWQHQMDEALKACIIHHKNIIEMKNEVSFLF
NFPIMGQFTVSVIIICLTMFQISLVSIASIKFLTLVLYQGCMLLEITLFCWFGNEIILKVSDSKLK
FSNPIFSCVLHGCYKCLQSSQLSTSVYNANWTDCSTSFKKNLLFLMMHTKDPLEIRAGNFVGVSLQ
TLSGIVRTSWSYFVVLQQVHDE
>hom13502.t1_1
MEDEPKPEEFKIRKLSFEFTRAIYHDKVKNTLLLSKWLLQGLCAWPDNNEKYIKFINILSLVNFFI
LETLHVLFVLSVYDDIATAADATTTVTAVVEGIVRTLIIVIKKKEINNMLCKVWFDFWPIKLIEPI
YAKKLVTKMRMTFMLPGGYLLLSIVCNTQIVGDPFVHHSGLIFQSIYPFKWNETYVYEALFFWQYI

GDWFTLFMINAFDFFMVTLVGIVIIQYMMLHEVIREILTEKSKRHRAVIFGEGWENVTDLEVLLKC
LEQHQTLIE
>hom13558.t1_1
MSTGMTKSSYAQDYFFINRRLLLYGGLTTLENPSALVYRLHKIYQVVVCISTIFLCPISIFIGAAE
NRDQIAKTIKCINAGLTILLGSIKSVFWLFKHKNIEDIMNTIESGEFDYEKVEDFDPGLLTKEAKV
TGFRYGLFFFIIGEMSLLMLYIPAYVSSLRYIINNGLVTNATFFKDTPYYTYIPFQHATPLTYITA
LTYECIPINMIAINIIGIDTLFMNILNFIGVHLVILRGAFRSIRARSLKKINGPPLTEDGLNNSNE
LEEQMLREMKKSIKHLQKIIX
>hom16287.t1_1
MDRPDHDALDRFFEVNVFILKTGGVWIPSKNRPFRERFLRYFYNFSLLSYCLLIYQPAETGVLFTD
VSLIVFIRSLRDQFNHFICVYKIFRWFGKRTEILDIMRVLQSKEFTYEDYGDFRPAMIIKDHKEKA
DRWMKMFLYGVNGICFNMCVSVFYVFLFKHEDFYRKDDNGNLIYDQKLPVTLTTPFRQDTRLGFLL
HFIFEIFPLDIYGWIIIGLDTLFTSIMSCISAHLCILQGAFSTIRLRCLRRLGLPDDEVLHNTTEI
DVEMLNEINKCIRHLQSLIK
>hom17026.t1_1
MRFLDDRALFSELIDGLYTVQCAANLQFNYFGMVYRHVPAMRLFEELQNFDNFGVPKQLARTARKV
TYVNRGLFTYCAGGPFGVCLFNFVVRQACLAQNEKTGREETCGYTTYAVYPYSIAASPFFEIHLFI
STFYVVYMCGGGTTFACACYTAVEYINVRIEHLKSMLRKLSQVKSHQRDEDFRFCVRYHQYIISFD
IRLKKDIMFMIRRAQEPLTLRVGSFGHLSLPLLKQVDVIIDVLKYSQKSKVREYGQKRATKNLQKY
IDDLPTDEILNVIKTGINRQDFSSFLNYLLLAFTDTTESHEKRFRVFETVLEELFKRNVSTSNSNT
ITTRLSMELEKLKSEHLVALCSVCTERIQKGNIADTSWQELLPKLLQVLIERECVEYNDITVTGLE
YKTQYVNSLCMLQWSPNIVIPLTAMFIEIPLMKDEHLQVVSKLGQYMEKLTCQEIPSFVYQFLKLC
QYQNGRSIFLLLQNYFGIRIYNRVQKDNNFTSSETIDLIDDTLDDQAIQAESTVLFHIYQSALHGY
DSIKDYLSSLKNLTKSPEFVLHPFQLTALLTISTVTFYEDRVFTIIRTCISRNFHEEIKKSESLWF
KDMIPLPVNMNAVIKQIIEFSVQERDLVLQGLVNLGFILLGVGLQLGKNTLVEKQWTLGKFILLSL
IKQKPHIAHAILQRLCNCIISGQNASQCIDCFHSLTRKFALITLENKSCVVELMEALLQASGSVAT
SILDAVIPLTKLSPTIRDHLILLLRKALYSRITETRQMAVKGFLKMLKNLKISNMAALSQMSSSNS
STSHSILTQISLTRAGQSATSVFTNEALCFELLGILRRCFMQQAEVRARLYDGLYDAVCMNPDIGE
PVLNLLWSHFGDYYVIDQDVLPPLQFSKITLTKDVDVTLQEPLGKLIHTFGIIVTKVLESDEENAS
AAKFAKILGSLCDRMSKCELVHLELDDGTNLLDVLPECQQKLIILKEAMCVYEALIGFKLSSWRKT
SENYAQDVNRLFQGYSRLLQFFKNLSKPRKGGRKKKDVNKTTTQQSESSSKHDSQLKAKSIKLSNT
LLDMRVLIKFLHLLHGNVNWTTVSQANLLKPKHGIHRHMMQATIQQFQAIKNEKFPGHHDSKLYYN
YCTDVGSILYERCIVRFEDFLDFDCTTAILTLDCFHIILQLICSNYHDDLKLFLSLISGNESEEDF
LVQLKSFIEIYQKLFELDEDDAPTDPEIKKLPQIILSTLTLLVEQIPTEINDLSIQVYWMKQFACR
KTVNNKLITTNFATLWFNTYLKFKSGTMIFEKIVDNLHEVAGTMGDVSFTRGLENKTITNFKAVEV
REKFNLVNELSAHSLIQCLTHVLKVMLDDAEWIISRLRSEYLMFTYPGEQGSDRRREYLKNKERGT
CCHMCLLISLVNSLCNVLIPTGVLSENVLKVLALLYNTLNSLAKYFLLRSTKVNVAFQPARFEAVV
KMVGKQLSPNVHKFILNIEDMQKEEMGNPDVKKKSVDSLKTKVLRETKLIPKVVFEIEQLSKTVIQ
LSNKTKVDLSKFIGQGTTRDFRIKDLKSVMEKNQGLGDVSLSTDVPCSNQTVDDNSNSESDECPPS
KKSKI
>hom17585.t1_1
MSRGGQKEFEIVKNAIQIYNRQACFFYVIVLSIVTQSTLHAFLDPGHDEISIDSLNNITNIKHIRL
TPFSIWLPFKPVKTPLYEIASLYQIACGLFFGFVIGAIDAIICGMYLHIKAQFLILKNVLDNYLKI
AANLIENVDIDVSIYENSKHLSNGIHIVEKIPPILQKYVDIVVKGCVEHHQDIIDLSEGVEDTFCN
LX

Pheromone-binding protein

>abi3665.t1_1
MALTDEQKEKVKQHYKECVAETGVAEELVQKGRKGEFADDPKLKQFVFCFFKKVGFQNEQGDLQLD
VIRAKIPTDVNKNEAEDVIKACKDITGTDAADKAFSVYKCYWNSTPNHIALV
>abi4098.t1_1
MQGNDQILAGELEELEEENNLYPTNPRHRRDGENSVKEEKCRRKDVKSCCEEEFLQSLKDADRVLK
KECYREITGKNHSSHLADPFRCENIEEHKRDITVDDKDNFKIEEMTKYLNEKLSSHEWLKQQMDVI
INKCVTEANETDAKRENDDKGACSPAAIKLGHCFWREIQIACPTNLIKDERKCNKIREEIKKKDNL
GFDV
>abi7033.t1_1
MEELGRQLHTECQEQTGVAEELIKTIQDKKGFPDDNKLKCYIKCLMAEMAVIDDDGVIDVEAAVAV
IPEEMKDNVEPTMRKCGVKIGSDPCDSAYLTHKCYYESNPEVSLKIA
>abi7666.t1_1
MQTLLSTLVVFVALFTVTIGYNFDDAVYNQILANNFDDFAPDAQNFISNHRFRRDAEHKCHGRHSS
CCNEDVFQKMHEDGKETFKECFKTVTGKEFGEGPPKDPFSCENMEKRKKEIIVDDDGNVKPDDAKQ
FLKEKFADIEWIQSHIDEYVDKCATEAKEEAAKFDASDKDACNPSGLKFKHCLWKSVQLGCPADQI
TDENKCKKIREYIDKHGDVFPPPPLPHIH
>abi7667.t1_1
MIYHLNILFCHFADEILTSELQELLSGNVYGNVRVRRDDEAAKCQSKHPLSGLKSCCSNDIVDHYH
EADRPIMLECRNELYGKENQSDVPRECGNVHANKDLYLCLGQCVGQKLGYIDSNGKLLPDKLRNSI
KEKLSKIQWLQPELDTVINKCIGEVESANKESSEEGKCKPGASTIGHCVWREIQFACPAEEITDVE
NCEKIRNYLNTKPQIP
>abi7668.t1_1
MRIFIICVLLGVNRAYDFEGDYHNQNLNKNGNHPFTRIRRDDLADECQEEIKSCCNEEIFKRVHDA
DYEIHKECYKLMISNDTEVEPLNIYACKHPLTQKEITCVTQCVVQKKGLIDSKGNLIVEEFLARIP
EIYSDVEWLMPKIEDFFQKCNAQLNEFLEDHDTNDGCNPSAIKMQSCFWKEIQMNCPADEIQDEEK
CTKIRENIMNDATE
>abi15452.t1_1
MKQRANIEQKNNEEQEIGFLAVLCVTTASKADQQRAKEIVAKLLNECKTKVGATDADIQAIRDKQI
PKTKTGMCLIDCIFDKTHITKDGMLDKEGTIQAFTTVLQGNKMKLKTLSDLLDVCVKETSSNNMDR
CTNAAKIVECSSKHGKVFGLDV
>abi19709.t1_1
MPKTVFIRTILYCGESLDARKERIRKYREECIAETKVDPQLIDKADGGEFSDTRELQCFAKCFYTK
AGFITKDGELLLDVVKAKIPPEQDRETALGIIEKCKSLKGKDVCETAYAIHKCYFQNAHAAGKKE
>abi21550.t1_1
ALTDEQKEKLKQHYKECFAETGVAEEVVQKGRKGEFIDDPKLKQFVFCFFKKVGFQNEQGDLQLDV
IKAKVPTDVNKNEADDVIKACKDTTGTDAADKAFNVYKCYWKSTPNHVSLI
>abi22385.t1_1
MKYLLIFIAVAVVVVSGVPQSQKDKIKAFSRECISVTGVDKELLKQAKEGHFVDDEKLEDFTFCLF
KKMGLQDENGEILINVARSQLPAELSEADKDDIVNKCFSKKGVTNX
>hom6835.t1_1
MIYHLNILFCHFADEILTSELQELLSGNVYGNVRVRRDDEAAKCQSKHPLSGLKSCCSNDIVDHYH
EADRPIMLECRNELYGKENQSDVPRECGNVHANKDLYLIDSNGKLLPDKLRNSIKEKLSKIQWLQP
ELDTVINKCIGEVESANKESSEEGKCKPGASTIGHCVWREIQFACPAEEITDVENCEKIRNYLNTK
PQIP
>hom15093.t1_1
MKAAAKLIRNTCRQKTKATDEQIDNMHKGIWDEDQNAKMKKNGAFDLELANKQLPQLPENRKEPTV
NSMNKCKDAGGDKTEKCDLSYAFFKCMYFDNPEVTKDGHMDVESANRQILTLPEPRQAPSKRSLDE
CKDVGSMLSDKCDIGYEIAKCLYFSNPKVSKWFYP
>hom18384.t1_1
MEPQSIVTVLFLCAFILTEQVLGENSSEKLHKKVCMREANVNPSLIEKAYSGDFANNRELKCFFRC
YYLESGFINEAGEIQIDEIKSKIPRKVNGKLAQEAIDKCKNTKGMDSCETAFEMQKCLHANRLTV

Cryptochrome

```
>abi2631.t1_1
MSGSLVAPQLADGGGPGPDKHVVHWFRKGLRLHDNPSLREGLKGAATFRCVFVLDPWFAGSSNVGI
NKWRFLLQCLEDLDRSLRKLNSRLFVIRGQPADALPKLFKEWGTTVLTFEEDPEPFGRVRDHNITT
LCKELGINVVQKVSHTLYHLQNIIDRNGGRAPLTYHQFLAIIACMGAPPTAEPPVITKTLCGAHTP
LSEDHDEKFGVPSLEELGFDTEGLLPPVWQGGESEALARLERHLERKAWVASFGRPKMTPQSLLPS
QTGLSPYLRFGCLSTRLFYYQLTDLYKKIKKTFPPLSLHGQLLWREFFYCAATKNPNFDKMLGNPI
CVQIPWDKNAAAIAKWASGQTGFPWIDAIMTQLREEGWIHHLARHAVACFLTRGDLWISWEEGMKV
FEELLLDADWSVNAGMWMWLSCSSFFQQFFHCYCPIKFGRKADPNGDYIRKYLPVLKNMPVRYIHE
PWTAPESVQRATKCIIGKDYPLPMVNHALASKINIQRMKQVYQQLAKYRTLESEHCKLPIHKDGYQ
AQVVTVGNPNNMNDH
```

Accessory gland protein

```
>abi3689.t1_1
MTSQASNKMHPLSAVGYITTVHHDKPPETKVSVMASKMKLHELQLNATLAPGERQKRLLPYVNFYS
QHNFNYPNMQPHVSPQRYQLVAPKSTNFQSFHHKYNEPTGTNFQPKLVDSRRVIQPNVRLTPFTDS
NALPGPFIPIRTTPQSVNYDAVQQIINLPNYGAMYEKLSQLKLPQQNGQTGPQVYNQNPRYDTPQF
IVNYIRKPQINYIPIREFQQHQDAPGIKPQQQTTKTIIQIYTPTNVDVSPSARPQYEQSSQRNPVK
SYNEQQTKTQNAIHIETPTQNTNDERSQHTQEIPIRPIIIFRMKSNEQKLKDERRKIFEEQLKIYE
DQQRISANQQNLYNEKLQIDHPKQYATIQKVIPQIDQHYHLVPIEKYHDIVTSNYKTDHLRNKIVT
EQNLEPIRIQESLGNPKLEENSATEVYGYVTEIPHNEYQGIISKYEPIPDSVSEQKLTQTLESENA
IITTPKSDIHLNKETIEYPINNYHVSNPTTPENDNSLGEILKNLQETNALPQSLTPENIDNSIKTL
VKILNVLKKQQRLSKKPIIVAEQQKDEYESDIDNETEVGLGGYDEETITEFFPADTPEGGTPGKPG
IDYPALSTIPRTSFTCKTQRYKGFFGDPETHCQVREVIV
```

Insulin-like peptide

```
>abi12684.t1_1
XINGGFLNMYKRDNFKVCGKQLSGLLSVVCQSRYAGKRAGIDDLDVFDYDTHEEEDNEYKVNRPFK
SRLETFGIIPYQYIKNSGQKRGIVDECCHASCIYRQEKVRVCGKQLRKLLGEMCKSRYYGKRNDLF
QENIYDDNEMKDVGDYINYPVRSRMLAFALIPNKFRKNYADKPIQTEKICRTCLSEEGDMRSVFSM
DESVGETTRLFEMLMSCSSVQVNEGDGLPSQVCLQCVHYITRAYSFKQLCERSDSTLRQLLGFPTQ
TFVELKPYPQSMVELIMPVQSQIELKPLDPKIELCLDTEYPKIDASDSDTGNTNSQLESKPKKEPK
KRKIKDDTEQPIYPCEECTQCFTILSDLKAKFIRKLHDTFVKFVIRDLQVLVHDGAKRHLCSECGK
GFSRSDDLTRHLRTHTGEKPFPCTLCGKSFAQSFRLLEHRRAHANEKSFICSACGKAFSRYTSLAA
HNKTHSGIKSHECGICGKRLCGSGSLTMHMKTHSGVKDHICPYCGKGFTTPSNLIIHKRTHTGERP
YVCNVCGKGFPDPSRLTVHTRSHSGEKPYVCSVCGRGCVSSSQLKKHLRIHTGEKPYQCTLCPKAF
PRSEDLRIHVKTHTGNRNHVCVICKKGFYQASTLKVHMRIHTGEKPYTCNICDKAFSQTGPLSTHM
KTHTDIEKIPAPVVTLR
>abi17550.t1_1
MHCREIILLNIWRICNKIRAAIFRVGINLWDFYLPLDPNKNCLISESQFVSVLSGPLRGTIGLSDE
EISELADYFRVQDGRVFYTQLCEVIHDSVPDFAKNAPLVTGLEWEDPLHVNRLSTTEERRLSLLIT
KIAALVNLRKLVLRPYFQDYELIAKNNGTVTIAHFARVLAYLGIMVSADDFNLLVKKFLKDSYTLN
YVAFVSAVDSAVQFMEQHGMLDLGGNILNQFPGRIIDAELPKLPRPEIGKILASSIFGKQSIFHPA
LNAPPEIQDLITVIRRIQRHVLENRLRVSEYFRDFDPLNCGKISISQFHRGLDLLGISGIQRLYLS
LPEIQSVMVQYRDPVDPSRVCWKTFEDDIEQVFTTKELEKSPCLCVDPPPLEVAEMSKPGAKLWQN
VNGSMRDLCEDAVEKVKHKVMRRRILLKPVFRDCDKHNNGHVSRSQMRQCLLSNGILLSDEELYAL
EERFNDDVGFNYFWFLTEAEPKPYEEPLYTGFLADMQKINAPKPRKPVDRREKDIVLILAKIKGKV
VRERIRVIEFLIDFDRCNEQLIKREDFKRGLSVCRFDLTENEVDTIMDVFASPMRPDCVDYRRFSE
VIEESFTQSCLERAPLIVPLQHVPTKDCERNFLNFEERLTLSVAMQKLSKKPELQMNLMSIFQDYD
RTNCGTISQEHFLKALSVRGMYNLISRTEFDMICKCFSYERGLRDEVDYRAFIKALDILHATDNFV
RILDMVAIFQMWFPVSTVAALCVLLDNADATVSGKELDLIFKDRSKSQWEEAWHRERYSRCRETLL
RHLYWACEKDIYRITRRSGGNDYKLFPSESEMLDFPWITEKRAKHLLRYKRSSRRSGSSITAECCR
SSGCTWEEYAEYCPTNKRYVSYV
>abi21226.t1_1
MCLIVLLFEESGAQQENMVQVCGKHLRSLLSIICDSKYAGKRSNNLSIRPEYDYVDEQQKDYSEEQ
PAMSYMPYPHKTKMQAYSLIPYKYKRSAGQKRGPGIVEECCHKPCHINELSGYCEF
>hom11213.t1_1
XINGGFLNMYKRDNFKVCGKQLSGLLSVVCQSRYAGKRAGIDDLDVFDYDTHEEEDNEYKVNRPFK
SRLETFGIIPYQYIKNSGQKRGIVDECCHASCSMKQLRAYCEF
```
