# Supplementary figures and images for: Draft genome of a biparental beetle species, Lethrus apterus
Source: BMC Genomics. 2021 Apr 26;22:301. doi: 10.1186/s12864-021-07627-w (PMC8074431; doi:10.1186/s12864-021-07627-w)

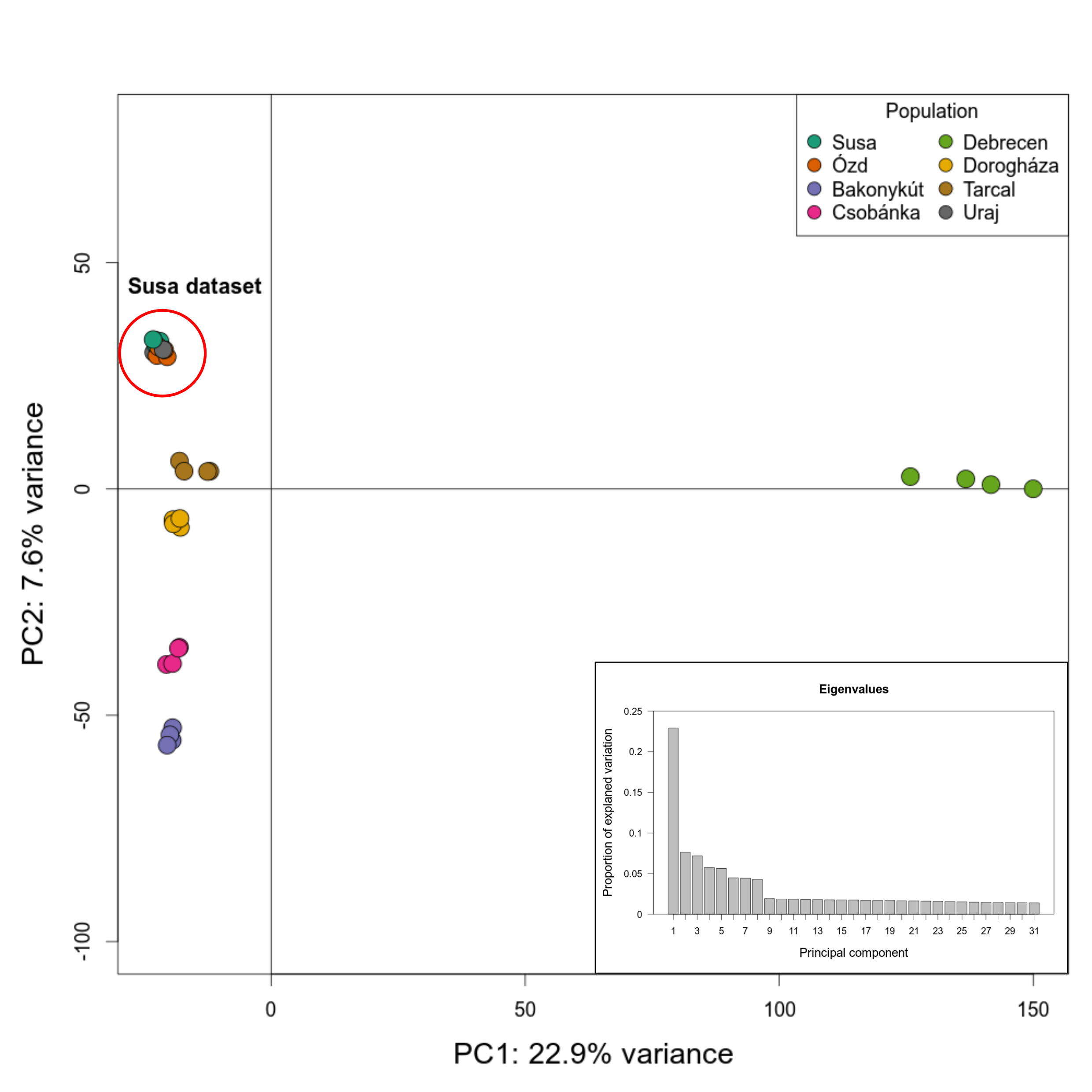

Supplement: Supplementary file 2 — Additional file 2: Figure S1. Principal component analysis plot of samples from eight populations of Lethrus apterus. The first two components (PCs) are plotted and the sample names are included. Eigenvalues are shown in the bottom right corner. The red ellipse includes the samples forming the Susa dataset. [file 12864_2021_7627_MOESM2_ESM.png]
