# Supplementary material for: Draft genome of a biparental beetle species, Lethrus apterus
Source: BMC Genomics. 2021 Apr 26;22:301. doi: 10.1186/s12864-021-07627-w (PMC8074431; doi:10.1186/s12864-021-07627-w)
Supplement: Supplementary file 3 — Additional file 3: Table S1. Information on the NCBI accession numbers of the raw reads for each sample. [file 12864_2021_7627_MOESM3_ESM.pdf]

Table S1. Information on the NCBI accession numbers of the raw reads for each sample.

Sample name	SRA accession number	BioSample ID	BioProject ID
B150	SRR13594341	SAMN17614387	PRJNA695433
B168	SRR13594340	SAMN17614388	PRJNA695433
B145	SRR13594329	SAMN17614389	PRJNA695433
B158	SRR13594318	SAMN17614390	PRJNA695433
B35	SRR13594315	SAMN17614391	PRJNA695433
B45	SRR13594314	SAMN17614392	PRJNA695433
B33	SRR13594313	SAMN17614393	PRJNA695433
B39	SRR13594312	SAMN17614394	PRJNA695433
BK14	SRR13594311	SAMN17614395	PRJNA695433
BK9	SRR13594310	SAMN17614396	PRJNA695433
BK1	SRR13594339	SAMN17614397	PRJNA695433
BK3	SRR13594338	SAMN17614398	PRJNA695433
CO13	SRR13594337	SAMN17614399	PRJNA695433
CO15	SRR13594336	SAMN17614400	PRJNA695433
CO5	SRR13594335	SAMN17614401	PRJNA695433
CO6	SRR13594334	SAMN17614402	PRJNA695433
DB14-16	SRR13594333	SAMN17614403	PRJNA695433
DB54	SRR13594332	SAMN17614404	PRJNA695433
DB13-8	SRR13594331	SAMN17614405	PRJNA695433
DB39	SRR13594330	SAMN17614406	PRJNA695433
DP1	SRR13594328	SAMN17614407	PRJNA695433
DP2	SRR13594327	SAMN17614408	PRJNA695433
DP20	SRR13594326	SAMN17614409	PRJNA695433
DP32	SRR13594325	SAMN17614410	PRJNA695433
TA13	SRR13594324	SAMN17614411	PRJNA695433
TA3	SRR13594323	SAMN17614412	PRJNA695433
TA10	SRR13594322	SAMN17614413	PRJNA695433
TA11	SRR13594321	SAMN17614414	PRJNA695433
UR1	SRR13594320	SAMN17614415	PRJNA695433
UR16	SRR13594319	SAMN17614416	PRJNA695433
UR13	SRR13594317	SAMN17614417	PRJNA695433
UR15	SRR13594316	SAMN17614418	PRJNA695433
